# Immunogenomic characterization in gastric cancer identifies microenvironmental and immunotherapeutically relevant gene signatures

**DOI:** 10.1002/iid3.539

**Published:** 2021-09-28

**Authors:** Xiao Han, Heyue Lu, Xiaojun Tang, Yao Zhao, Hongxue Liu

**Affiliations:** ^1^ Department of Gastrointestinal Surgery Affiliated Huaian No. 1 People's Hospital of Nanjing Medical University Huaian Jiangsu Province P. R. China; ^2^ Clinical Medicine Affiliated Huaian No. 1 People's Hospital of Nanjing Medical University Huaian Jiangsu Province P. R. China; ^3^ Department of Obstetrics Affiliated Huaian No. 1 People's Hospital of Nanjing Medical University Huaian Jiangsu Province P. R. China

**Keywords:** immune checkpoint, immunogenomic phenotype, immunotherapy, prognosis, tumor microenvironment

## Abstract

**Background:**

Multiple molecular subtypes with distinct clinical outcomes in gastric cancer have been identified. Nonetheless, the immunogenomic subtypes of gastric cancer and its mediated tumor microenvironment (TME) characterizations have not been fully understood.

**Methods:**

Six gastric cancer cohorts with 1506 samples were obtained. Unsupervised methods were used to perform immunogenomic phenotype clustering. The least absolute shrinkage and selection operator regression method was used to construct immunogenomic characterization score (IGCS).

**Results:**

Three distinct immunogenomic phenotypes were determined. We observed a prominent survival difference between three phenotypes. The TME cell‐infiltrating characteristics under these three phenotypes were highly consistent with three immune subtypes of tumors. Cluster 1, was characterized by the “immune‐desert” phenotype, with relatively lower cell infiltration level (type 1 “cold tumor”); Cluster 2, characterized by “immune‐inflamed” phenotype, with abundant innate and adaptive immune cell infiltration (“hot tumor”); Cluster 3, characterized by “immune‐excluded” phenotype, with significant stromal activation and inactivated immune cell infiltration (type 2 “cold tumor”). We demonstrated IGCS signature was significantly correlated with TME inflammation and stroma activity, molecular subtypes, genetic variation, microsatellite instability, immune checkpoint molecules, and patient prognosis. High IGCS subtype, with poorer survival and enhanced stromal activity, presented an immune‐exclusion and non‐inflamed TME characterization. Low IGCS, related to increased mutation/neoantigen load and microsatellite instability, showed enhanced responses to anti‐checkpoint immunotherapy. Four immunotherapy cohorts confirmed patients with low IGCS exhibited prominently enhanced clinical responses and treatment advantages.

**Conclusions:**

This study demonstrated the immunogenomic characterizations could play a crucial role in shaping the complexity and diversity of tumor microenvironment. Targeting tumor immunogenomic characteristic in order for changing adverse phenotypes may contribute to exploiting the novel immunotherapy combination strategies or novel immunotherapeutic drugs, and promoting the advance of tumor personalized immunotherapy.

## Graphical abstract

Three immunogenomic subtypes with distinct clinical, microenvironment, and immunotherapeutic characteristics were determined; The IGCS signature was significantly correlated with TME inflammation and stroma activity, genetic variation, microsatellite instability, immune checkpoint molecules, and efficacy of immunotherapy; the immunogenomic characterizations could play a crucial role in shaping the complexity and diversity of tumor microenvironment.

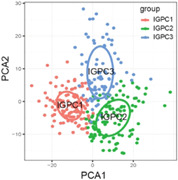

## 1 INTRODUCTION

In the previous decade, although technological advances and novel mechanistic insights have altered strategies for gastric cancer treatment, only a limited number of patients could benefit from these improvements in overall survival, with less than 20% 5‐year survival.^1^, ^2^ However, anti‐checkpoint immunotherapy represented by the anti‐PD‐1/PD‐L1 antibodies, offered outstanding survival benefits to a small group of patients with robust responses. Unfortunately, the benefits are either minimal or non‐existent to the majority of patients, far from meeting a clinical necessity. Additionally, both within and across the tumor types, there is an existing disparity in clinical response rates, suggesting the presence of intrinsic and adaptive immune resistance to immune checkpoint blockade.^3^, ^4^, ^5^

Recently, with the development of cancer genomics, the classification of gastric cancer has changed from the traditional histological subtype to the molecular subtype. The Asian Cancer Research Group described four molecular subtypes of gastric cancer using gene expression data including the subtypes of epithelial–mesenchymal transition (EMT), microsatellite instability (MSI), microsatellite stability (MSS)/TP53+ and MSS/TP53−.^6^, ^7^, ^8^ TCGA project has also categorized gastric cancer into four subtypes based on the comprehensive depiction for its molecular landscape, encompassing chromosomal instability (CIN), MSI, genome stable (GS), and EBV.^6^ Despite our deepened understanding of the molecular subtypes of gastric cancer, the immunogenomic phenotypes as well as their induced tumor microenvironment (TME) cell‐infiltrating characterizations of gastric cancer remain poorly known. Investigating the distinct immunogenomic phenotypes in the role of TME cell‐infiltrating complexity and heterogeneity formation will advance the existing knowledge on the antitumor immune response of TME, which could guide and exploit more effective immunotherapeutic strategies.^9^, ^10^ However, owing to technical limitations, most research focuses on one or two immune cells, which may lead to the biases cognition of the overall cell infiltration characteristics of TME.^11^, ^12^, ^13^, ^14^ The recent progress in next‐generation sequencing creates opportunities to identify the immunogenomic changes in gastric cancer effectively. Additionally, founded on the immunogenomic profiling from the tumor tissue RNA‐Seq, numerous computational methods estimating TME cell infiltration abundance has been developed. Dissecting the TME cell infiltration patterns induced by distinct immunogenomic phenotypes could be possible by utilizing immunogenomic data and calculation methodology. Also, the capabilities for predicting patients' responsiveness to immunotherapy are likely to improve from this process.^4^, ^5^, ^9^, ^15^, ^16^, ^17^ In this study, we integrated the immunogenomic profiling to comprehensively evaluate the immunogenomic phenotypes of gastric cancer, and correlated the immunogenomic phenotypes with the TME cell‐infiltrating characteristics. We successfully defined three immunogenomic phenotypes with distinct TME cell infiltration patterns and immune escape mechanisms in gastric cancer based on 1506 samples. We demonstrated that the tumor immunogenomic characterization played a crucial role in the TME heterogeneity and complexity formation as well as immunotherapeutic response differences between individuals.

## 2 METHODS

### 2.1 Gastric cancer cohorts and preprocessing

We included six gastric cancer cohorts with complete clinical annotation information including the TCGA‐STAD, GSE84437, GSE62254/ACRG, GSE57303, GSE34942, GSE15459.^7^, ^18^, ^19^, ^20^ For Affymetrix platforms, the affy R package was utilized to data preprocessing.^21^ For Illumina platform, we directly download the normalized matrix files. For TCGA‐STAD cohort, we downloaded the FPKM values of gene expression and then converted FPKM values into transcripts per kilobase million (TPM) values.^22^ The sva package was used to correct the batch effects. All data analyses were based on the Bioconductor packages and the R software (version 3.6.1). The detailed information of each cohorts was listed in Table S1.

### 2.2 Immunogenomic phenotypes clustering

We performed unsupervised clustering for 730 immune‐related genes to identify the immunogenomic characterizations of gastric cancer. The immune genes were derived from GPL19965 immune profiling platform. We used the consensus clustering algorithm of ConsensuClusterPlus package to determine the optimal number of stable immunogenomic phenotypes. To guarantee classification stability, we repetitively conducted this analysis for 1000 consecutive times.

### 2.3 Functional annotation and gene set variation analysis

We conducted GSVA enrichment analysis to explore the characteristics of biological functions in these three immunogenomic phenotypes^22^, ^23^ based on “c2.cp.kegg.v6.2.symbols” gene set.^24^ The functional annotation for differentially expressed genes (DEGs) was carried out by the clusterProfiler R package.^25^ We used the limma package for identifying DEGs between distinct phenotypes with *p* < .001.^26^

### 2.4 Inference of TME cell abundance

We performed the single sample gene set enrichment analysis (ssGSEA) for the gene expression profiles to estimate the relative abundance of each TME cell subtype.^27^ (Table S2) We sourced the gene set to mark each type of TME infiltration cell from the study of Charoentong.^5^, ^28^

### 2.5 Generation of immunogenomic signatures

We used the Least Absolute Shrinkage and Selection Operator (LASSO) Cox regression to perform dimensionality reduction and select the most robust immune signature genes.^29^ The immunogenomic characterization score (IGCS) was then defined as follows:

$$\text{IGCS=}\sum_{\text{i=}1}^{n}\mathrm{Coefi}\times \mathrm{Expri},$$

where *Coefi* was the Cox Regression coefficient, and *Expri* was the immune signature gene expression.

### 2.6 Acquisition of the transcriptome data of immunotherapeutic cohorts

We analyzed the correlation between IGCS signature and the known signatures to further investigate the biological characteristics of IGCS.^30^ We eventually included four immunotherapeutic cohorts with complete corresponding clinical information and transcriptome data after systematically searching the public databases. Of these, the IMvigor210 cohort analyzed the PD‐L1 blockade effectiveness in patients with metastatic urothelial malignancies.^30^ GSE78220 cohort investigated the efficacy of anti‐PD‐1 antibody in metastatic melanoma.^31^ Patients in GSE93157 cohort were also intervened by PD‐1 inhibitors.^32^

### 2.7 Statistical analysis

We used the Kruskal‐Wallis and one‐way analysis of variance methods to perform a difference significance test for groups of three or more.^33^ The Wilcoxon test was utilized to perform the difference analyses between the two groups. The survminer R package was used to plot the survival curves, and the log‐rank tests was to perform the difference test. The optimal cut‐off point to classify patients into low and high IGCS group was determined by the MaxStat R package.^34^ The hazard ratios (HR) for survival analyses was calculated through the univariate Cox regression model. All statistical *p*‐values were two‐sided. The *p* < .05 was considered as a statistical significance. All data was processed through the software R 3.6.1.

## 3 RESULTS

### 3.1 Landscape of immune genome in gastric cancer

**Figure 1:**
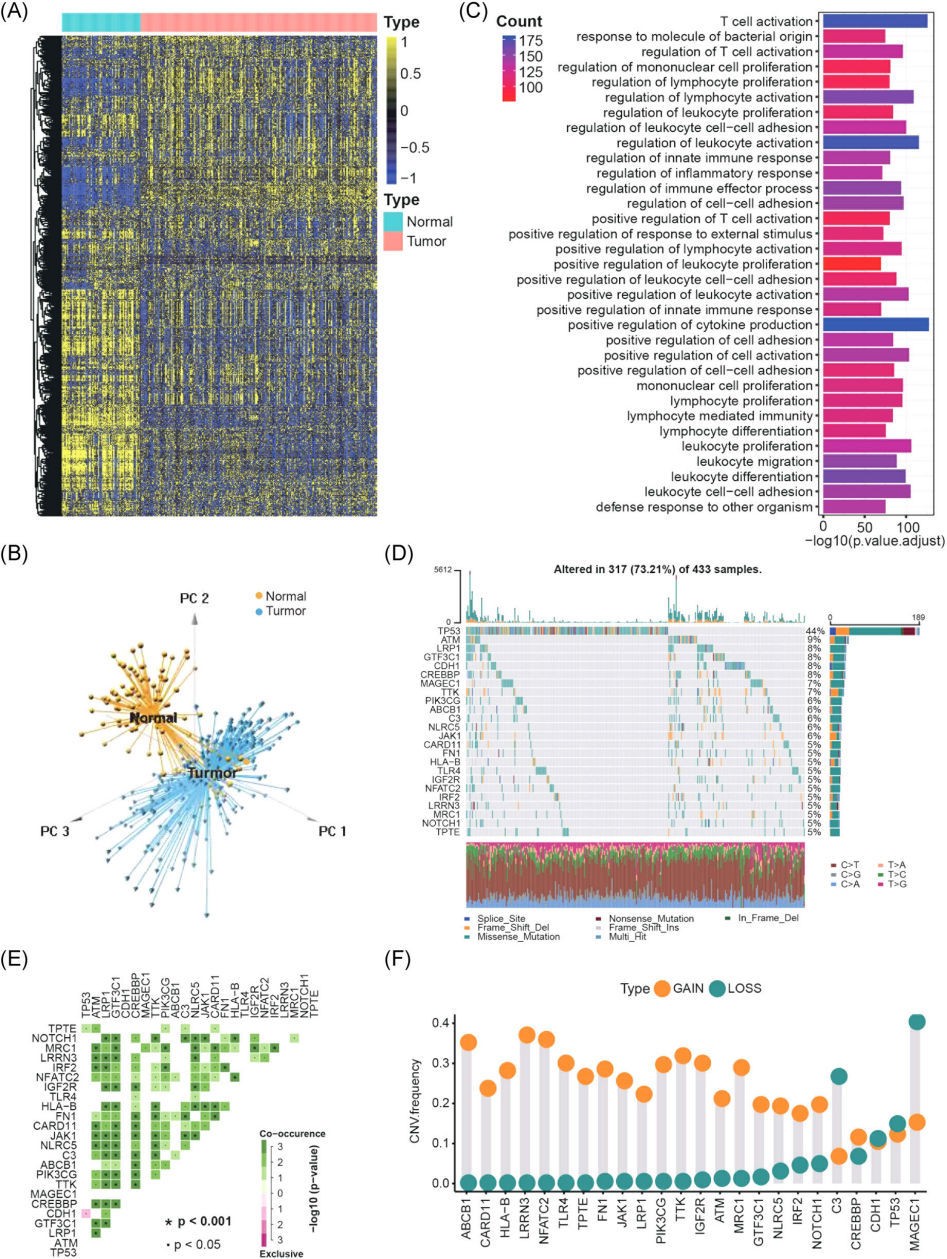
Landscape of immune genome in gastric cancer. (A) Expression of 730 immune‐related genes in tumor and normal samples based on hierarchical clustering. (B) Reducing dimension for the immune‐related genes by principal component analysis revealed two entirely disjoint populations. (C) Functional annotation for immune‐related genes via GO enrichment analyses. (D) The mutation landscape of immune‐related genes with high mutation frequency in TCGA‐STAD cohort. (E) The mutation co‐occurrence and exclusion analyses for 24 immune‐related genes with high mutation frequency

The scheme of gastric cancer immunogenomic phenotype identification and immunogenomic prognostic signature construction was shown in Figure S1A. In total, 730 immune‐related genes were extracted from five gastric cancer datasets (Table S3). We found there existed significant difference on the immune genome between normal gastric tissues and tumor tissues (Figure 1A and Table S4). Dimension reduction for these immune‐related genes using principal component analysis methods showed tumor samples and normal samples presented two completely disjoint populations, suggesting that the immunogenomic characterizations have undergone significant variation with the progression of gastric cancer (Figure 1B). Functional annotations showed these genes were significantly associated with immune regulation pathways (Figure 1C and Table S5). Then, we analyzed the mutational landscape of the immune genome in gastric cancer. Out of 433 samples, we observed mutations in at least one immune gene in 92.84% of the patients (data not shown). The results suggested that TP53, followed by ATM, demonstrated the highest mutation frequency. Here, we presented the mutation landscape of 24 immune genes with a mutation rate greater than 5% (Figure 1D). Further research discovered the marked co‐occurring mutation relationship between these highly mutated immune genes (Figure 1E). The copy number variation (CNV) alteration frequency study also revealed a widespread CNV alteration in highly mutated immune genes that mainly concentrated on copy number amplifications, while C3 and MAGEC1 had prevalent CNV deletion frequency (Figure 1F). We investigated the messenger RNA (mRNA) expression levels between normal and tumor samples to determine the potential influence of these genetic variations on the expression of highly mutated immune genes in tumors. The results showed that CNV alterations could be the prominent factors causing perturbations on the expression of immune‐related genes. The amplificated CNV of immune‐related genes in gastric cancer tissues was accompanied by the increase of their mRNA level (e.g., LRRN3 and NFATC2), and vice versa (e.g., MAGEC1) (Figures 1F and S1B). Survival analyses for highly mutated genes revealed their crucial effects on the prognosis of patients (Figure S1C and Table S3). The correlation analyses revealed the potential association among these genes (Figure S1D).

### 3.2 Identification of immunogenomic phenotypes in gastric cancer

**Figure 2:**
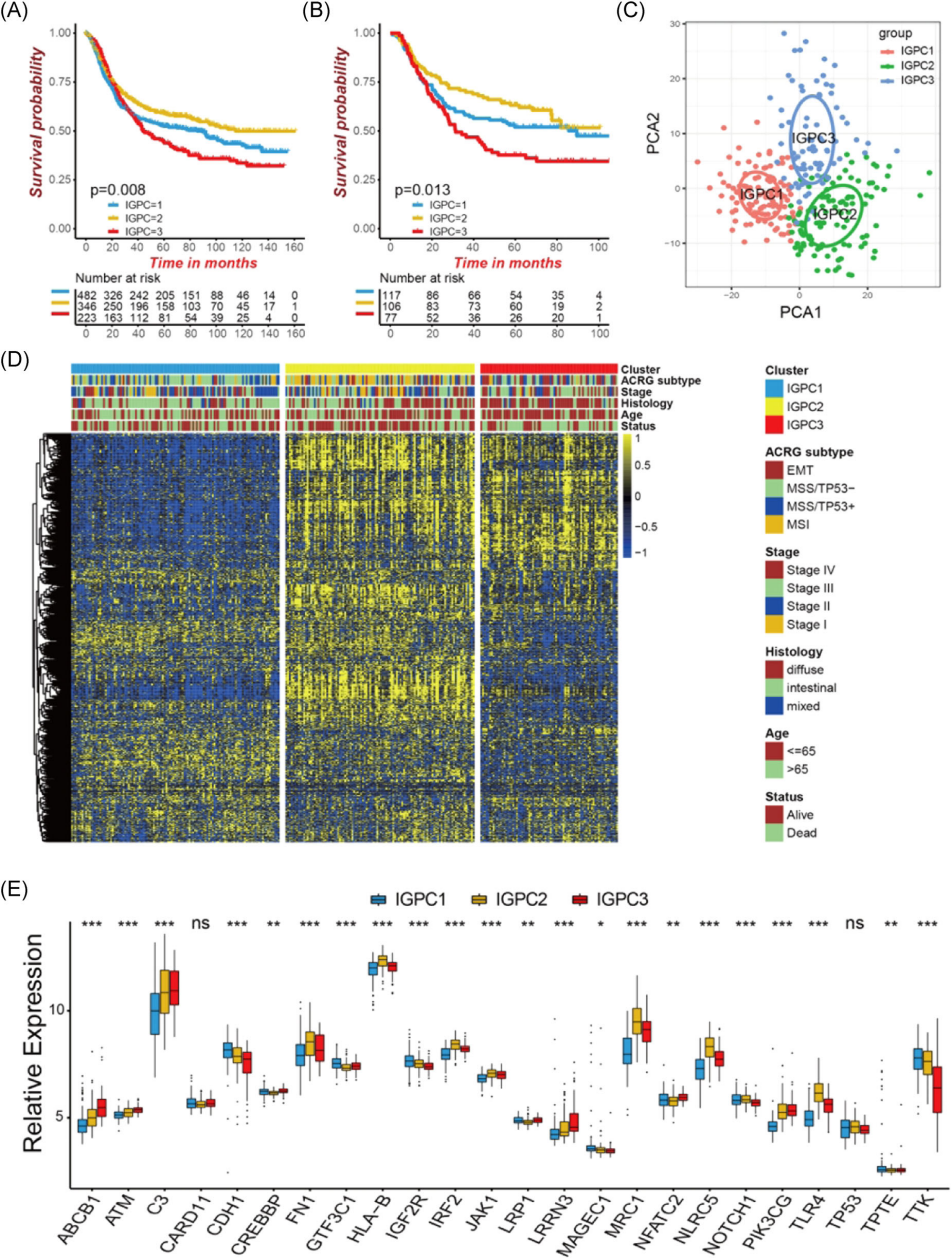
Immunogenomic phenotype clustering in gastric cancer. (A) Survival analyses for three distinct immunogenomic phenotypes based on 1051 patients from five gastric cancer cohorts. (B) Survival analyses for the three immunogenomic phenotypes in ACRG cohort. (C) Principal component analysis for the immune‐related genes in the three immunogenomic phenotypes. (D) Immunogenomic phenotype clustering in the ACRG gastric cancer cohort and hierarchical clustering of immune‐related genes among three phenotypes. (E) Immune‐related genes with high mutation rates expressed in the three immunogenomic phenotypes

We enrolled five GEO cohorts including GSE84437, GSE62254/ACRG, GSE57303, GSE34942, and GSE15459 into one meta‐cohort for further analyses. The unsupervised clustering was used to characterize the immunogenomic phenotypes in gastric cancer. We successfully classified all tumors into three distinct clusters based on expression of 730 immune‐related genes. We termed these clusters as IGPC1‐3 (immunogenomic phenotype cluster), respectively (Figure S2A–S2E). Prognostic analysis for the three distinct immunogenomic phenotypes revealed the particularly prominent survival advantage in IGPC2 subtype, and the survival inferiority in IGPC3 subtype (Figure 2A). The ACRG cohort, which included 300 patients with gastric cancer, integrated the most comprehensive clinical information. We, therefore, focused on the ACRG cohort to further investigate the features of these immunogenomic phenotypes in various biological behaviors and clinicopathology. Consistent with all patients clustering, we also discovered three fully distinct immunogenomic phenotype clusters in ACRG cohort based on the immune gene expression, including 117 samples in IGPC1 subtype, 106 samples in IGPC2 subtype, and 77 samples in IGPC3 (Figures S3A–S3D, 2B‐D, and Table S6). The significant distinction in immunogenomic profile was observed among the three clusters, confirming that there were indeed three different immunogenomic phenotypes in gastric cancer (Figure 2C,D). Survival analysis also revealed significant survival differences among the three phenotypes (Figure 2B). To note, patients with the diffuse histological subtypes were significantly clustered in IGPC3 phenotype, suggesting tumors in this phenotype presented a poorer differentiation. While patients in IGPC1 and IGPC2 phenotypes were characterized by the intestinal histological subtype, which were markedly correlated with a better tumor differentiation (Figure 2D). We then investigated the expression of immune‐related genes with high mutation frequency among the three immunogenomic clusters. We observed the highly mutated immune genes presented significant difference in expression between the three immunogenomic phenotypes (Figure 2E).

### 3.3 TME cell infiltration characteristics in distinct immunogenomic phenotypes

**Figure 3:**
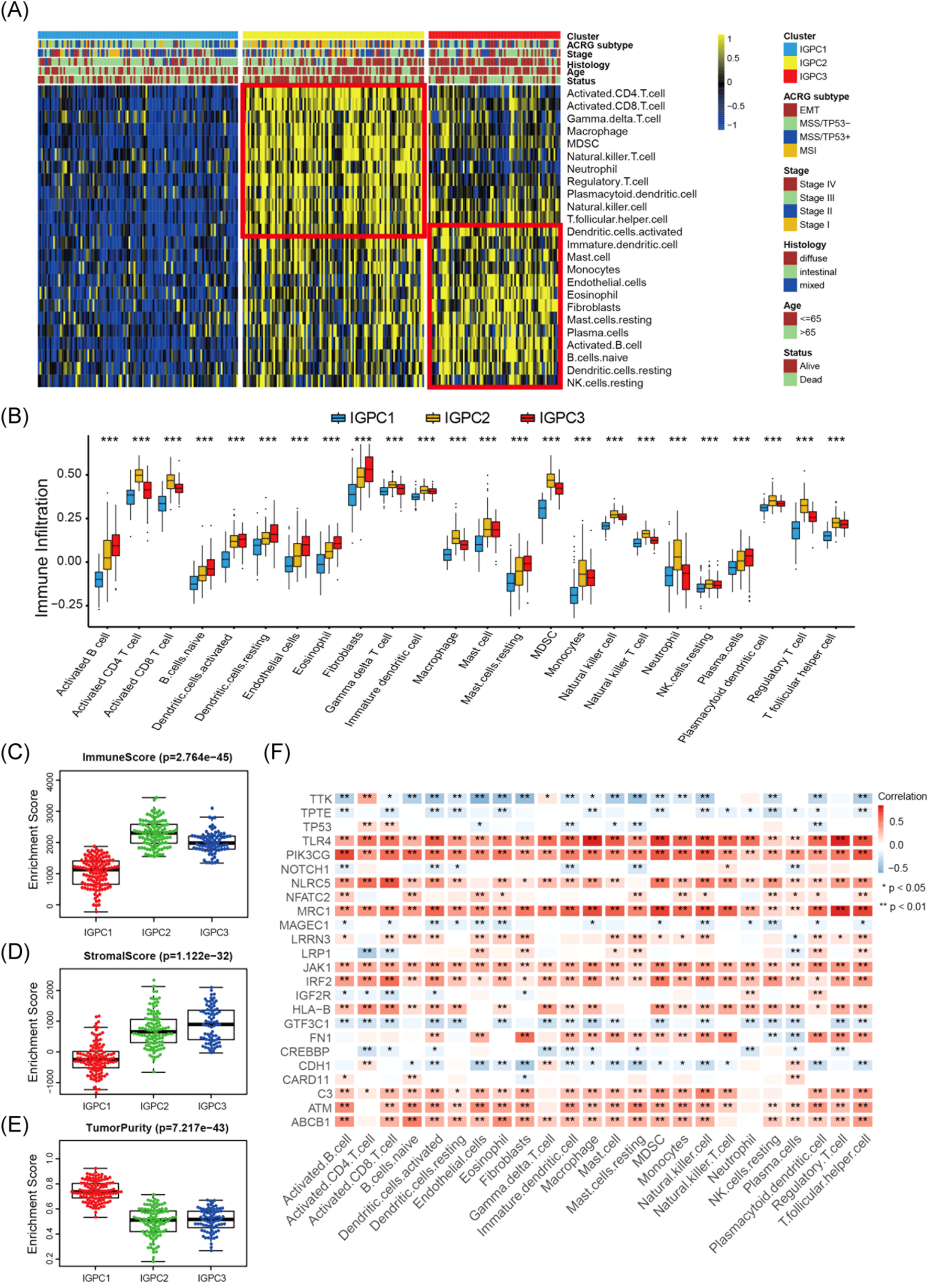
Characteristics of TME cell infiltration among the three distinct immunogenomic phenotypes. (A) The abundance of 24 TME cell infiltration among three immunogenomic phenotypes visualized by heatmap. (B) Differences of 24 TME cell infiltration abundance between three immunogenomic phenotypes. (C–E) ESTIMATE algorithm analyses revealing the overall TME cell infiltration landscape among three immunogenomic phenotype. (F) The correlation between highly mutated immune genes and TME cell subtypes. TME, tumor microenvironment

**Figure 4:**
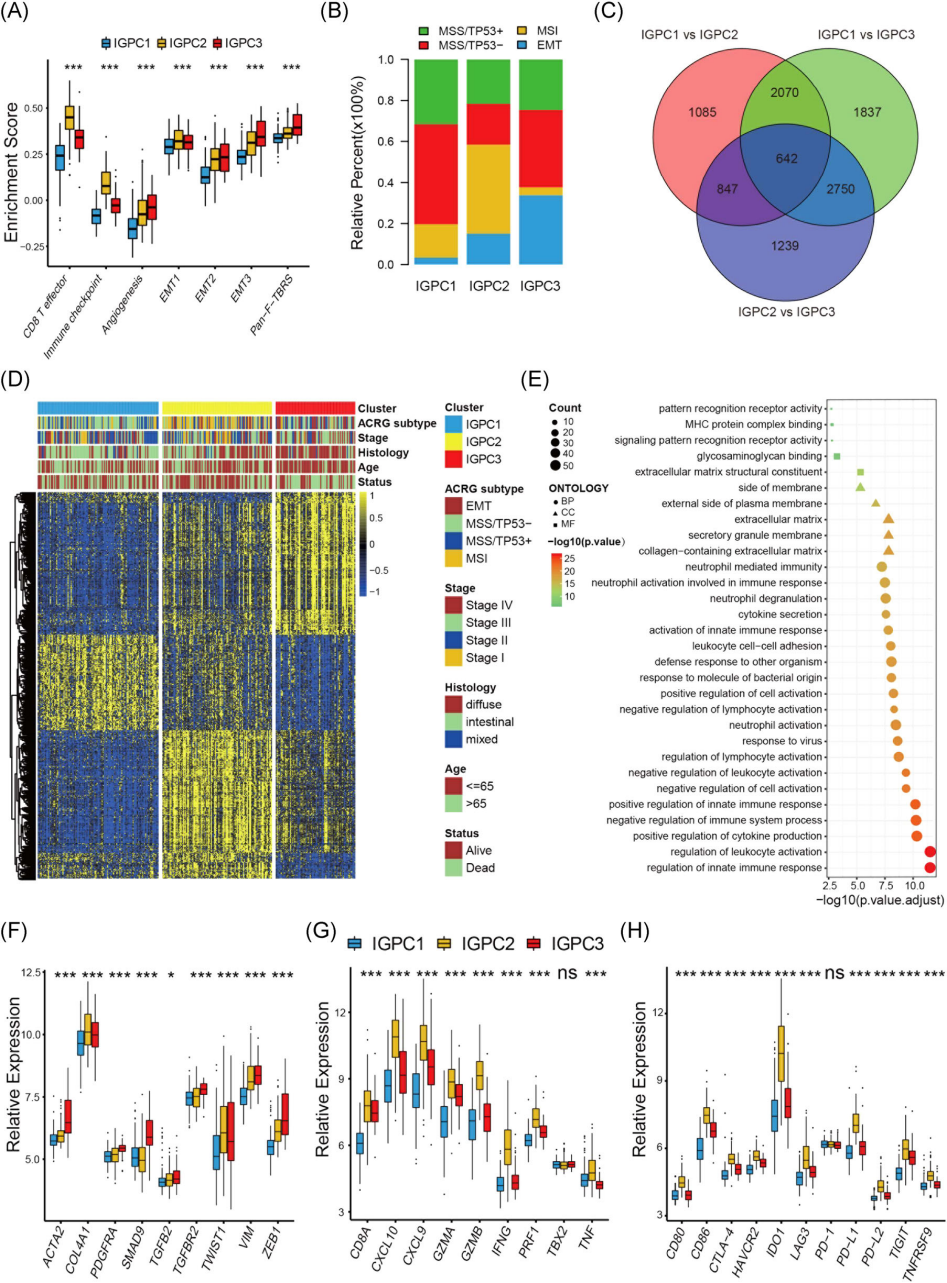
Transcriptome traits of distinct immunogenomic phenotypes. (A) Variations between three distinct immunogenomic phenotypes in pathways with stroma activation. (B) The proportion of ACRG molecular subtypes in the three immunogenomic phenotypes. (C) The venn diagram showing 642 immunogenomic phenotype‐related genes. (D) Hierarchical clustering of phenotype‐related genes among three immunogenomic phenotypes. (E) GO functional enrichment analyses for immunogenomic phenotype‐related genes. (F) Difference in the TGF‐β‐EMT pathway‐related gene expression among three immunogenomic phenotypes. (G) Difference in the immune‐activation‐related gene expression among three phenotypes. (H) Difference in the immune‐checkpoint related gene expression among three phenotypes. EMT, epithelial‐mesenchymal transition; TGF‐β, transforming growth factor β

We executed the GSVA enrichment analysis to investigate the biological behaviors amongst distinct immunogenomic phenotypes. As shown in Figure S4A–S4B and Table S7, we observed pathways correlated with immune activation were significantly upregulated in the IGPC2 phenotype including T‐cell receptor signaling pathway, Toll‐like receptor signaling pathway, cytokine–cytokine receptor interaction, and antigen processing and presentation pathways (Figure S4A). The stromal‐related pathways were prominently activated in the IGPC3 phenotype such as vascular endothelial growth factor, transforming growth factor‐β signaling pathways as well as the extracellular matrix receptor interaction (Figure S4A). While IGPC1 phenotype presented a significant enrichment in biological pathways associated with immune suppression (Figure S4B). According to the succeeding TME cell infiltration analyses, IGPC3 had unusually high levels of innate immune cell infiltration, including resting dendritic cells (DCs), mast cells, activated dendritic cells, and natural killer cells (Figure 3A,B and Table S6). However, we did not observe a matching survival advantage with the immune infiltration levels in this phenotype (Figure 2B). To further investigate the overall TME cell infiltration levels in each immunogenomic phenotype, we utilized the ESTIMATE algorithm. We found that despite the higher immune level in tumors with IGPC3 phenotype, the TME stromal activity of their microenvironment was significantly higher than other two phenotypes (Figure 3C–E). The stromal cells especially fibroblasts and endothelial cells were also markedly activated in IGPC3 phenotype, which could result in the loss of innate immune cell infiltration ability (Figure 3B). Based on the signature genes of stromal‐related pathways from Mariathasan's study, we quantified the activity of specific stromal pathways in the three phenotypes. Consistent with the above findings, the activity of stromal pathways was significantly enhanced in IGPC3 phenotype including the activation of pan‐fibroblast TGF‐β response (Pan‐F‐TBRS), EMT, and angiogenesis pathways (Figure 4A). Subsequent analysis revealed the clustering of most patients with EMT molecular subtypes into the IGPC3 phenotype, and patients with MSI molecular subtypes were mainly clustered into IGPC2 phenotype (Figure 4B). Based on the above results, it was found that the three immunogenomic phenotypes of gastric cancer had significantly distinct TME cell infiltration patterns. IGPC1 phenotype, characterized by relatively lower TME cell infiltration, was classified as immune‐desert cluster (type 1 “cold tumor”). IGPC2 phenotype, characterized by relatively higher innate and adaptive TME cell infiltration, was classified as immune‐inflamed cluster (“hot tumor”). IGPC3 phenotype, characterized by stromal activation and false innate immune cell infiltration, was classified as immune‐excluded cluster (type 2 “cold tumor”).

We then examined the correlation between each highly mutated immune gene and each TME infiltration cell type. We noted there existed a significantly tight relation between TME infiltration cell levels and highly mutated immune gene expression (Figure 3F and Table S8).

### 3.4 Transcriptome traits of distinct immunogenomic phenotypes

To further explore the potential biological characteristics of the three immunogenomic phenotype, we investigated the transcriptome differences between the three phenotypes and a total of 642 phenotype‐related DEGs were determined by using limma package (Figure 4C and Table S9). The univariate Cox regression model revealed their prognostic values in gastric cancer (Table S9). It was found the three immunogenomic phenotypes exhibited specific genomic characterization, respectively (Figure 4D). The enrichment analysis revealed these DEGs were significantly enriched in immune‐related biological processes, confirming again that tumor immunogenomic characterizations played a crucial role in shaping the TME cell infiltration complexity and diversity (Figure 4E and Table S10).

We studied cytokine and chemokine expressions that characterize these three phenotypes to explore further the function of tumor immunogenomic phenotypes in the TME immunoregulation.^29^, ^35^ The results revealed the significant upregulation of the mRNAs pertinent to the TGFb/EMT pathway in IGPC3 phenotypes, which indicated this cluster could be classified as stromal‐activated subtype (Figure 4F). IGPC2 phenotypes demonstrated increased expression of mRNAs associated with immune‐activation transcripts, making this cluster an immune‐activation subtype (Figure 4G). Immune checkpoint molecules that had an upregulated expression in IGPC2 phenotypes could lead to the immune escape of this phenotype (Figure 4H). The above outcomes reconfirmed the significant relevance of IGPC3 phenotypes to stromal activation as well as IGPC2 phenotypes to immune activation (Figure 4F–H).

DCs, a bridge that connect innate and adaptive immune responses, are accountable for antigen presentation and activating naive T cells. And, their activation relies on the increased expression level of major histocompatibility complex molecules, as well as adhesion and costimulatory factors.^36^ Here, we found significant differences in the transcripts of DCs activation molecules between the three immunogenomic phenotypes of gastric cancer. Consistent with the TME cell infiltration patterns, the expression of DCs activation molecules was prominently upregulated in tumors with IGPC2 phenotypes, suggesting a relatively higher TME cell infiltration level in this phenotype (Figure S3E).

### 3.5 Construction of immunogenomic signatures

**Figure 5:**
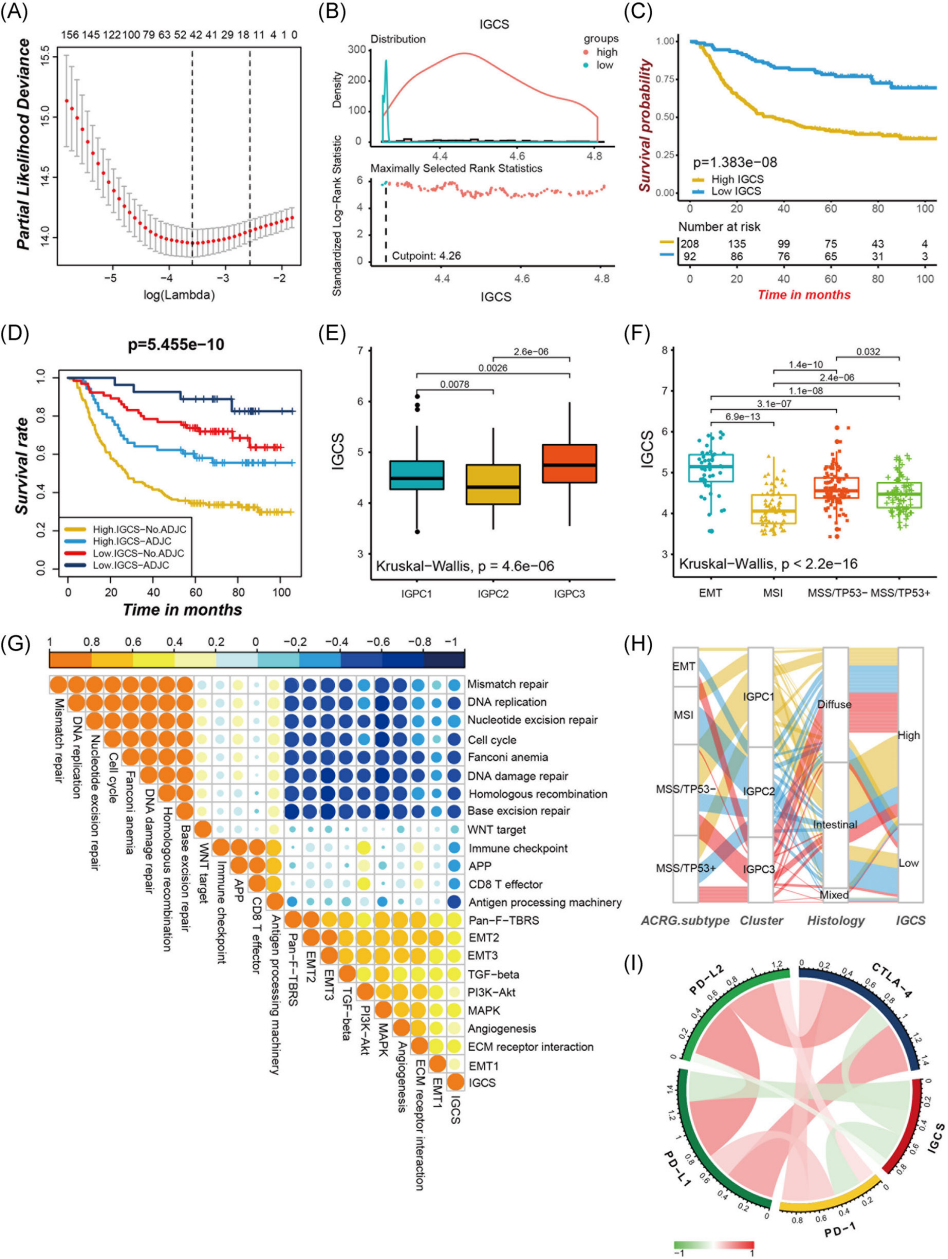
Construction of immunogenomic signatures. (A) The tenfold cross‐validation in the LASSO model was used to tuning parameter selection. (B) The MaxStat R package identified the optimal cut‐off point to dichotomize IGCS. (C) Kaplan‐Meier curves showing the survival difference between the lowand high IGCS subtypes. (D) Survival analyses of four subgroups, where patients were stratified according to adjuvant chemotherapy (ADJC) and IGCS signature. (E) Differences in IGCS across three immunogenomic phenotypes in the ACRG cohort. (F) Differences in IGCS across distinct ACRG molecular subtypes. (G) Spearman correlation between the known signatures and IGCS in the ACRG cohort. Orange marks depicted positive correlation and blue for the negative correlation. (H) The changes of clusters, ACRG molecular subtypes, histology and IGCS, visualized by alluvial diagram. (I) The correlation chord chart showing the mutual correlation between IGCS and immune checkpoint molecules. EMT, epithelial‐mesenchymal transition; IGCS, immunogenomic characterization score

To evaluate the immunogenomic characterization of individual tumor, we constructed the immunogenomic signature using the LASSO regression model. The IGCS was utilized to quantify the immunogenomic characterization of individual tumor as well as evaluate patient clinical outcomes. The IGCS was determined by the expression of 42 immune‐related genes, which were obtained from the LASSO Cox regression (Figures 5A and S5A). The coefficient was summarized in Table S11. Patients were classified as high and low IGCS subtype with the optimal cutpoint at 4.262 generated by the MaxStat algorithm (Figure 5B). A remarkable survival benefit was observed in the low IGCS subtype (hazard ratio [HR] 3.30 [2.13–5.11]; Figure 5C), with a 5‐year survival two times higher than the high IGCS subtype (77.0% vs. 40.8%). Our investigation for the IGCS signature in predicting the effectiveness of adjuvant chemotherapy in gastric cancer patients revealed that those with low IGCS experienced substantial treatment advantages (Figure 5D). The another outcome suggested that adjuvant chemotherapy did not interfere with the predictive power of the IGCS signature. Patients in the low IGCS group consistently presented significant survival advantage among those who are undergoing chemotherapy or not. Furthermore, the Kruskal‐Wallis test found significant variations on IGCS between immunogenomic phenotypes. IGPC2 phenotypes displayed the lowest median IGCS, while the IGPC3 phenotypes showed the highest median IGCS (Figure 5E). In contrast with the other three ACRG molecular subtypes, patients with EMT molecular subtypes exhibited a highest IGCS (Figure 5F). We then explored the potential relationships between IGCS and the known biological signature in gastric cancer for an improved illustration of IGCS biological features. The IGCS presented a significant positive correlation with the signatures related to stromal activated and a negative correlation with immune activated signatures (Figure 5G). These results firmly manifested the significant correlation of low IGCS with immune‐activation as well as high IGCS with stromal‐activation. Using IGCS signature may better assess the immunogenomic characterization of an individual tumor, and accurately identify its patterns of TME cell infiltration. The alluvial diagram also revealed tumors with poorer differentiation exhibited a higher IGCS, and better differentiation was associated with lower IGCS (Figure 5H and Table S12). Also, we found the significant negative correlation of the IGCS with the expression of immune checkpoint molecules, which suggested that the tumor immunogenomic characterization could have a potential influence on the immunotherapeutic clinical responses (Figure 5I and Table S13).

We then investigated the relationships between IGCS and clinicopathological features. The MSI subtype, which had a better prognosis, was significantly associated with a lower IGCS, while the MSS subtype with a poorer prognosis had a higher IGCS (Figure S5B). Multivariate Cox regression model analysis demonstrated that IGCS could function as an independent prognostic biomarker to assess patient outcomes (Figure S5C). The TCGA‐STAD cohort also confirmed the value of IGCS in evaluating patients' prognosis (Figure S5D). We also tested whether IGCS signature can be used to classify other types of cancer. We took colon cancer as an example and found that IGCS could also be used to predict the outcome of colon cancer patients. Patients with low IGCS have significantly prolonged survival compared to patients with high IGCS (Figure S5E).

### 3.6 Immunogenomic characteristics of TCGA molecular subtypes and tumor somatic mutation

**Figure 6:**
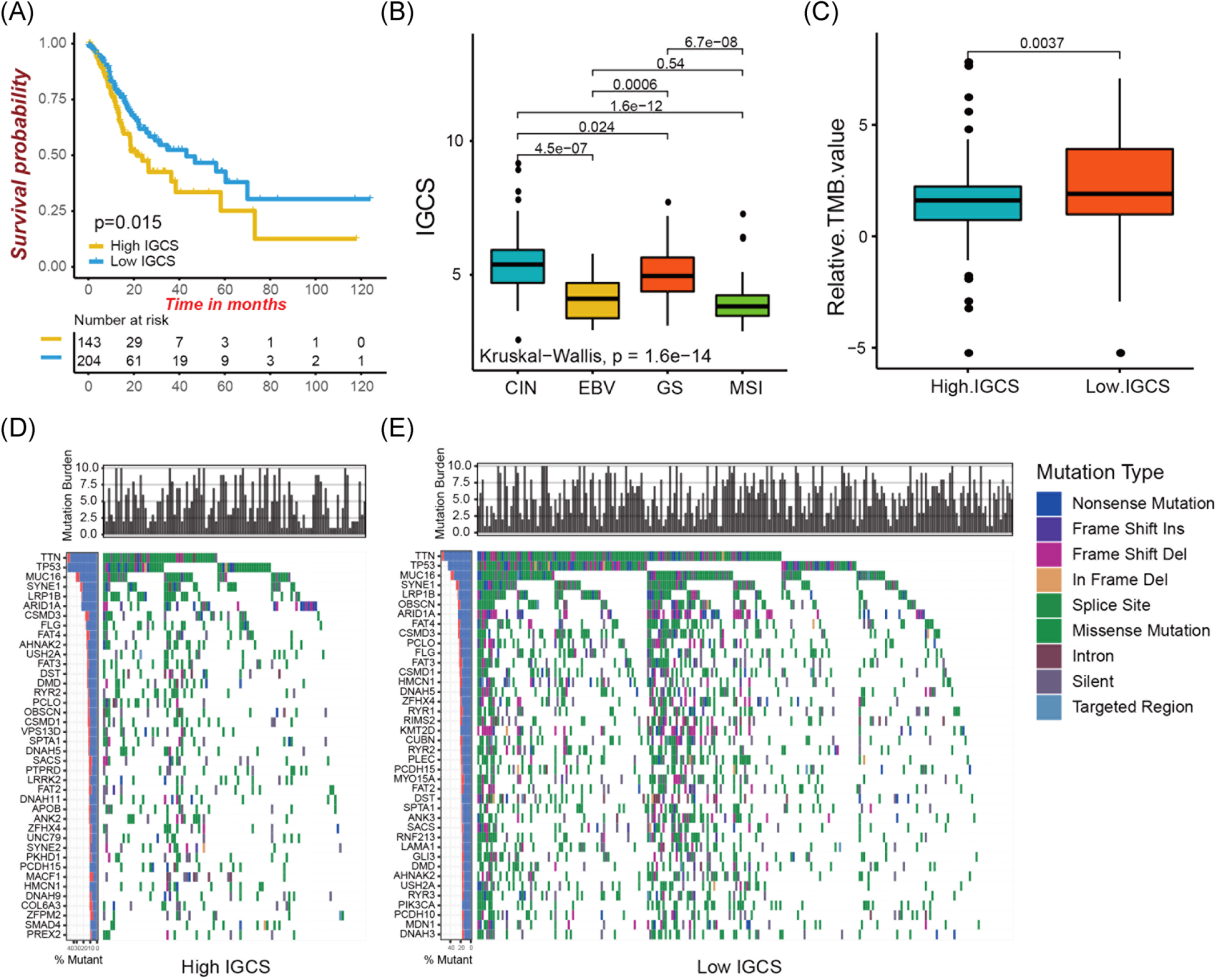
Immunogenomic characteristics of TCGA molecular subtypes and tumor somatic mutation. (A) Kaplan‐Meier curves showing the survival analyses of high and low lGCS subtypes in TCGA‐STAD cohort. (B) Differences in IGCS between distinct TCGA gastric cancer molecular subtypes. (C) Difference in tumor mutation burden between low and high IGCS groups. (D‐E) The waterfall plot showing the differences of TMB landscape between low and high IGCS groups. (D) High IGCS group. (E) Low IGCS group. CIN, chromosomal instability; GS, genome stable; IGCS, immunogenomic characterization score; MSI, microsatellite instability

TCGA project evaluated the exhaustive molecular characterization in gastric cancer, classified into four molecular subtypes that included CIN, EBV, GS, and MSI. We analyzed how IGCS differed across these molecular subtypes. The results highlight the substantial concentration of relatively higher IGCS on subtype CIN, with a worse patient survival. The better survival was evident in lower IGCS concentrated on subtypes MSI and EBV (HR 1.54 [1.09–2.18]; 5‐year OS rate, 25.1% vs. 38.0%; Figure 6A,B). Then, we studied the differences in the tumor somatic mutation distributions between low and high IGCS groups. Tumors with low IGCS were markedly correlated with a higher mutation burden (Figure 6C). However, we did not observed an obvious difference in mutation types between high and low IGCS groups (Figure 6D,E). We summarized the differences of TMB landscape between high and low IGCS subtypes using the waterfall plot (Figure 6D,E). For the same genes in each group, low IGCS always presented a significantly increased mutation rates compared to high IGCS.

### 3.7 Role of tumor immunogenomic characteristics in immunotherapeutic responses

**Figure 7:**
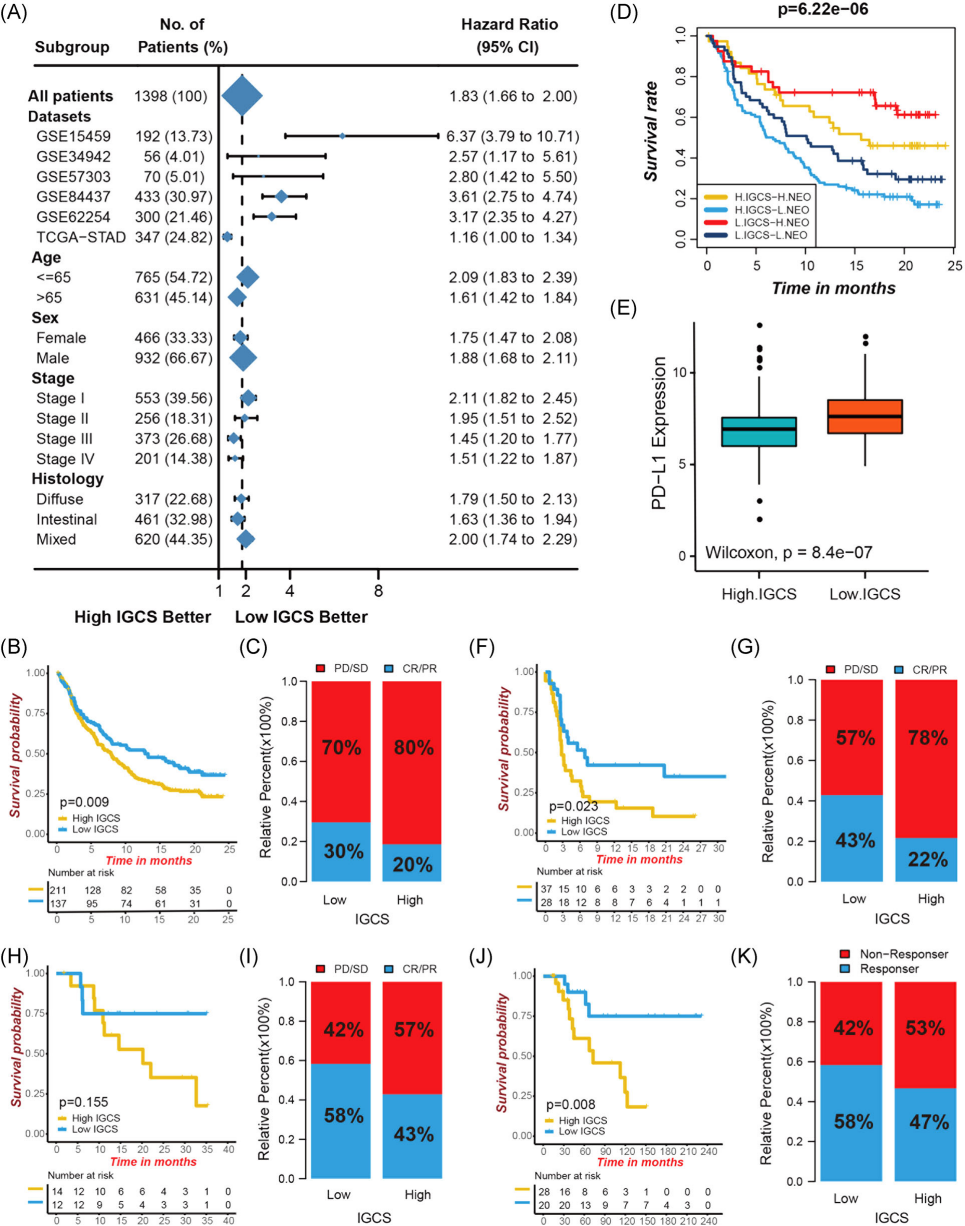
Role of tumor immunogenomic characteristics in immunotherapeutic responses. (A) Subgroup analyses revealed the prognostic value of IGCS as a continuous variable by using a univariate Cox regression model. (B) Kaplan‐Meier curves showing the survival analyses of high and low IGCS groups in the IMvigor210 cohort. (C) The ratio of clinical response types in high IGCS and low IGCS groups in the IMvigor210 cohort when treated with anti‐PD‐L1 immunotherapy. (D) Survival analyses for patients receiving anti‐PD‐L1 immunotherapy stratified by both neoantigen burden and IGCS signature. (E) Differences in the PD‐L1 expression in high and low IGCS subtypes in IMvigor210 cohort. (F) Kaplan‐Meier curves displaying the survival difference of high and low IGCS groups in GSE93157 cohort. (G) The ratio of clinical response types in high IGCS and low IGCS groups in the GSE93157 cohort when treated with anti‐PD‐1 immunotherapy. (H) Survival analyses for IGCS in GSE78220 cohort. (I) The ratio of clinical response types in each group in the GSE78220 cohort. (J) Survival analyses for IGCS in TCGA‐SKCM cohort. (K) The ratio of clinical response types in each group in TCGA‐SKCM cohort. IGCS, immunogenomic characterization score

To further examine the IGCS stability and substantiate its prognostic value, we applied the IGCS signature into other independent cohorts of gastric cancer (GSE15459, HR 4.27 [2.81–6.49]; GSE34942, HR 2.34 [1.08–5.08]; GSE57303, HR 3.11 [1.53–6.35]; GSE84437, HR 3.09 [2.34–4.08]; Figure S6A–S6D). The prognostic value of IGCS signature was well validated in a combined set of all GEO cohorts (HR 2.88 [2.42–3.43]; Figure 7E). The IGCS could be also utilized to predict relapse‐free survival in gastric cancer patients (GSE62254, HR 2.86 [1.91–4.27]; Figure S6F). Then, our study extended the IGCS signature to all gastrointestinal tumors to evaluate the patient outcomes comprising hepatocellular carcinoma, pancreatic adenocarcinoma, colon adenocarcinoma, cholangiocarcinoma, and esophageal carcinoma (HR 1.60 [1.30–1.98]; Figure S6G). The significance of IGCS signature for predicting outcomes in all gastric cancer patients especially in patients with distal metastasis were confirmed via the receiver operating characteristic curves (Figure S6H–S6I). In addition, we investigated the ability of IGCS as a continuous variable to predict patient outcomes using the univariate Cox regression analyses. The subgroup analyses revealed the significant prognostic predictive values of IGCS signature among all clinical stages and histological subtypes (Figure 7A).

The emergence of immunotherapies represented by immune checkpoint blockade has become a major breakthrough in curative therapeutic strategies for cancer. Using four immunotherapy cohorts, we explored the values of IGCS signature in predicting patient response to immune checkpoint blockade therapy. In the IMvigor210 cohort intervened by anti‐PD‐L1 regimens, patients in low IGCS group, compared to those in the high IGCS group, experienced a remarkable treatment advantages and clinical benefits as well as a markedly prolonged survival (HR 1.43 [1.09–1.87], Figure 7B; response 30% vs. 20%, Figure 7C). Furthermore, there presented a notable survival advantage among patients possessing both high neoantigen burden and low IGCS (Figure 7D). Patients with low IGCS also exhibited an increased PD‐L1 expression, which demonstrated a potentially enhanced response to anti‐PD‐L1 immunotherapy in this group (Figure 7E). These results indicted the quantification for tumor immunogenomic characteristics was a promising and robust biomarker for evaluating clinical responses and survival outcomes of patients when treated with immunotherapy. Subsequent analyses for other three cohorts with anti‐PD‐1 or anti‐CTLA‐4 immunotherapy regimens well confirmed the crucial role of IGCS signature in predicting patient response to immunotherapy (GSE93157 cohort, HR 2.01 [1.09–3.72], Figure 7F,G; GSE78220, HR 1.29 [0.38–4.40], Figure 7H,I; TCGA‐SKCM cohort, HR 4.10 [1.32–12.79], Figure 7J,K).

## 4 DISCUSSION

As insights on the tumor microenvironment advance heterogeneity and complexity, growing evidence underlines the crucial role of TME in mediating immune escape and treatment resistance to immunotherapy.^1^, ^3^ However, since most studies concentrated on only one immune gene or one TME infiltration cell subtype, the overall TME cell‐infiltrating patterns driven by distinct immunogenomic characterizations remain unknown. Comprehensively dissecting the immunogenomic characterizations of gastric cancer will help enhance our understanding of different TME cell‐infiltrating patterns as well as guide more precise immunotherapeutic strategies.^4^, ^5^, ^12^ More importantly, identifying the immunogenomic phenotypes could contribute to revealing the potential biomarkers significantly asscociated with clinical response to immunotherapy, and the novel immunotherapeutic targets may be found.^6^, ^9^

Here, we integrated the genomic profiling of 730 immune‐related genes and revealed three distinct immunogenomic phenotypes in gastric cancer, which had substantially different TME cell infiltration characteristics. IGPC1 phenotype was characterized by the immunosuppression, classified as immune‐desert cluster (type 1 “cold tumor”). IGPC2 phenotype was characterized by activated innate and adaptive immunity, classified as immune‐inflamed cluster (“hot tumor”). IGPC3 phenotype was characterized by stromal activation and inactivated innate immune cell infiltration, classified as immune‐excluded cluster (type 2 “cold tumor”). Although IGPC1 and IGPC3 were both classified into so‐called “cold tumors,” they had obviously distinct TME cell‐infiltrating patterns. We emphasized the TME activated stroma in the role of tumor immune escape. The type 2 cold tumors, similar to inflamed tumors, was also characterized by the presence of abundant immune cells. However, unlike the immune‐inflamed tumors and instead of infiltrating the tumor parenchyma, these immune cells remained preserved in the stroma surrounding the tumor cell nests. The stroma could penetrate the tumor parenchyma or be limited to the tumor capsule, making it seem that the immune cells are actually inside the tumor. The anti‐PD‐1/L1 agents can also stimulate the activation and proliferation of stroma‐associated T cells but not infiltration, and clinical responses are uncommon.^35^, ^37^, ^38^, ^39^, ^40^ Recent studies have revealed a negative correlation between oncogenic pathway activation and immune infiltration levels.^41^ However, few studies paid special attention to the mechanisms of impaired immune penetration. We showed that the stroma signaling pathways of angiogenesis, EMT and TGF‐β were significantly activated in tumors with IGPC3 phenotype characterized by type 2 cold tumor cell infiltration, suggesting the loss of ability of immune cells to penetrate into tumor parenchyma may be mediated by these pathways.

Currently, the transformation from “cold tumor” to “hot tumor” has become a significant direction in cancer research. Previous studies showed the decreased expression of PD‐L1, CD47, IL1β, CCL23, and CCL5 mediated by MYC amplification could induce the inactivation of macrophages and DCs, as well as limit the recruitment of natural killer cells, T cells, and B cells.^42^, ^43^, ^44^ Compared to the complex formation mechanism of type 1 cold tumor, changing the TME cell infiltration patterns of type 2 cold tumors mediated by IGPC3 immunogenomic phenotypes may be more clinically practical. In this study, we demonstrated that the tumor immunogenomic phenotypes could mediate the formation of TME cell infiltration diversity, suggesting targeting the immune genome of gastric cancer in order for transforming its immunogenomic phenotype could be potential strategies to change TME cell infiltration patterns. Based on the obtained signature immune‐related genes from LASSO, we then constructed IGCS signature to further evaluate the immunogenomic phenotype in individual tumor as well as its TME cell infiltration characterization. IGPC3 phenotype was characterized by high IGCS and IGPC2 phenotype characterized by low IGGS. We also revealed IGCS as a robust and independent prognostic biomarker. We revealed that IGCS was significantly correlated with microsatellite status, tumor mutation burden, and immune checkpoint molecule expression, suggesting that the immunogenomic characterizations could play a crucial role in mediating patient clinical responses to checkpoint immunotherapy. Four immunotherapy cohorts confirmed that in patients receiving anti‐checkpoint immunotherapy, low IGCS group was associated with a prominently improved clinical benefits and a markedly prolonged survival compared to high IGCS group. Generally, the clinical response to immunotherapy was approximately 12% higher among low IGCS groups than high IGCS groups.

Our research has several translational clinical values. Our results could help guide the screening of suitable patients for immunotherapy. We discovered an immunogenomic phenotype distinguished by “hot tumors,” accounting for about 33% of all gastric cancer patients while revealing notable therapeutic benefits and clinical advantages of immunotherapy in this cluster. More importantly, we also found an immunogenomic phenotype with type 2 cold tumor cell infiltration characterization (approximately 21% of all cases) and revealed its potential mechanisms of impaired immune penetration. Repairing the impaired immune penetration and releasing the microenvironmental antitumor immunity of such patients should probably be a more clinically practical direction for expanding the benefit population of immunotherapy.

There were several limitations in our study. Due to the lack of patient body fluid sample data such as serum, plasma, and so forth, we could not test whether IGCS signature was used for early diagnosis of gastric cancer. At present, we are actively collecting patient's body fluid samples to further verify the early diagnosis value of ICGS signature. In addition, for patients with distal metastasis, the prediction efficiency of IGCS is excellent, and its AUC value reaches 0.984. However, for patients without distal metastasis, the prediction efficiency of IGCS can be further improved, and larger samples are still needed to test the prediction value of IGCS signature.

## 5 CONCLUSIONS

This study indicted a comprehensive dissection of immunogenomic characterizations will help enhance understanding the mechanisms of TME diversity and complexity, as well as guide more precise immunotherapeutic strategies. The transformation of “cold tumors” into “hot tumors” based on immunogenomic characteristics could represent a step toward personalized immunotherapy in gastric cancer.

## CONFLICT OF INTERESTS

The authors declare that there are no conflict of interests.

## AUTHOR CONTRIBUTIONS

Xiao Han, Hongxue Liu, Heyue Lu, Xiaojun Tang, and Yao Zhao were responsible for the design of this study. Xiao Han, Hongxue Liu, and Xiaojun Tang performed the integration and analyses of the data. Xiao Han, Heyue Lu, and Yao Zhao wrote this manuscript. Heyue Lu, Xiao Han, Hongxue Liu, Xiaojun Tang, and Yao Zhao revised the manuscript. All authors approved this manuscript.

## Supporting information

Supplementary information.

Supplementary information.

Supplementary information.

Supplementary information.

Supplementary information.

Supplementary information.

Supplementary information.

Supplementary information.

Supplementary information.

Supplementary information.

Supplementary information.

Supplementary information.

Supplementary information.

Supplementary information.

Supplementary information.

## Data Availability

All data related to this study can be acquired from the Gene‐Expression Omnibus (GEO, http://www.ncbi.nlm.nih.gov/geo) and the GDC portal (https://portal.gdc.cancer.gov/).
