# Supplementary material for: Immunogenomic characterization in gastric cancer identifies microenvironmental and immunotherapeutically relevant gene signatures
Source: Immun Inflamm Dis. 2021 Sep 28;10(1):43–59. doi: 10.1002/iid3.539 (PMC8669697; doi:10.1002/iid3.539)
Supplement: Supplementary file 1 — Supplementary information. [file IID3-10-43-s008.pdf]

Figure S1. Workflow of our study and prognostic analyses of 24 immune genes with high mutation

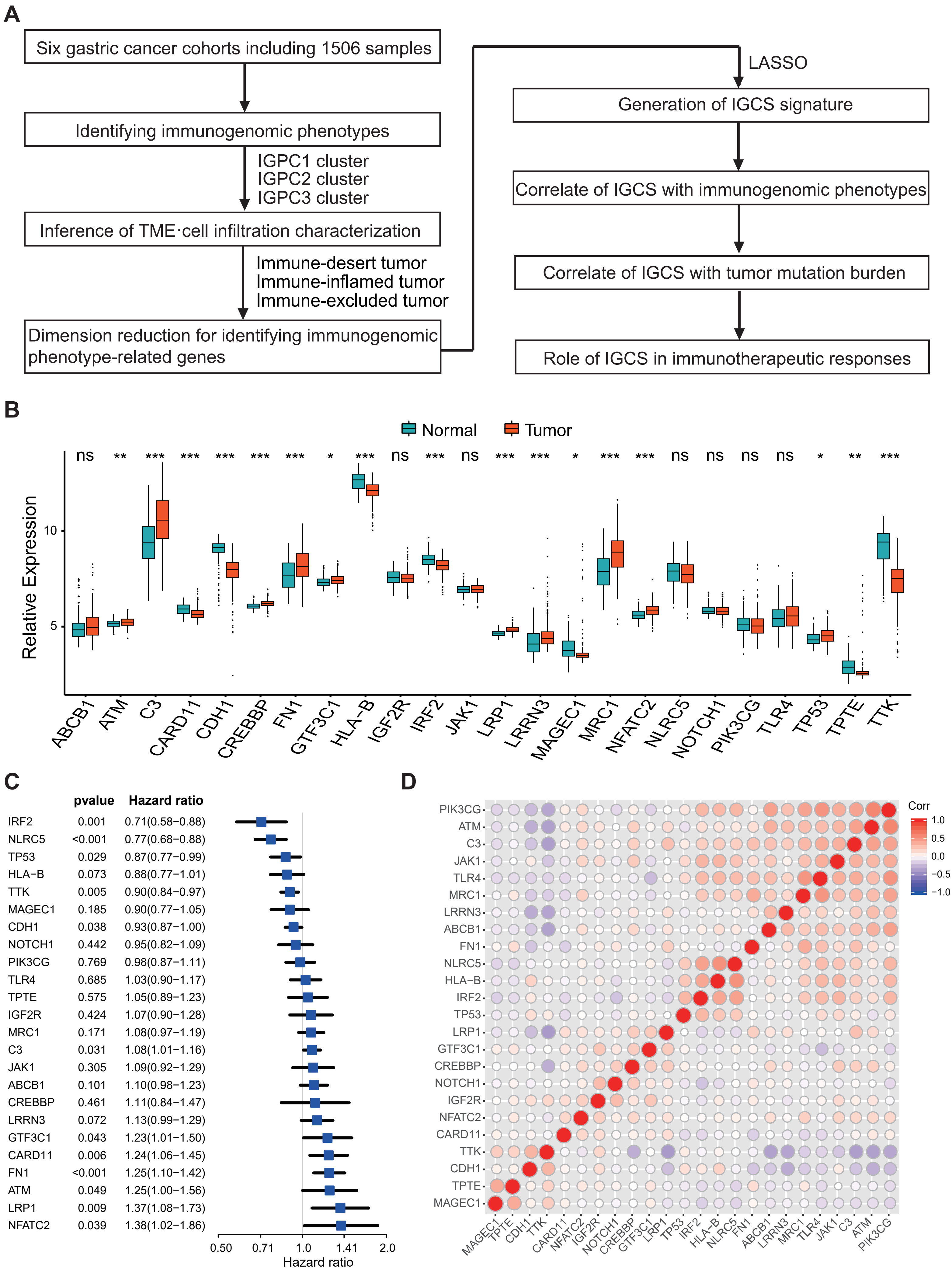

(A) Overview of our study. **(B)** Expression of 24 highly mutated immune-related genes between normal and tumor tissues. Tumor, red; Normal, blue. The asterisks represented the statistical p value.(*P < 0.05; **P < 0.01; ***P < 0.001) **(C)** Correlation between each highly mutated immune-related gene using spearman's correlation analyses. Positive correlation, red; Negative correlation, blue. **(D)** Survival analyses for the highly mutated immune-related genes based on the univariate Cox regression model. Hazard ratio more than 1 represented risk factors for prognosis while hazard ratio less than 1 represented protective factors for prognosis.

Figure S2. Unsupervised clustering of immune-related genes in the all gastric cancer cohorts.

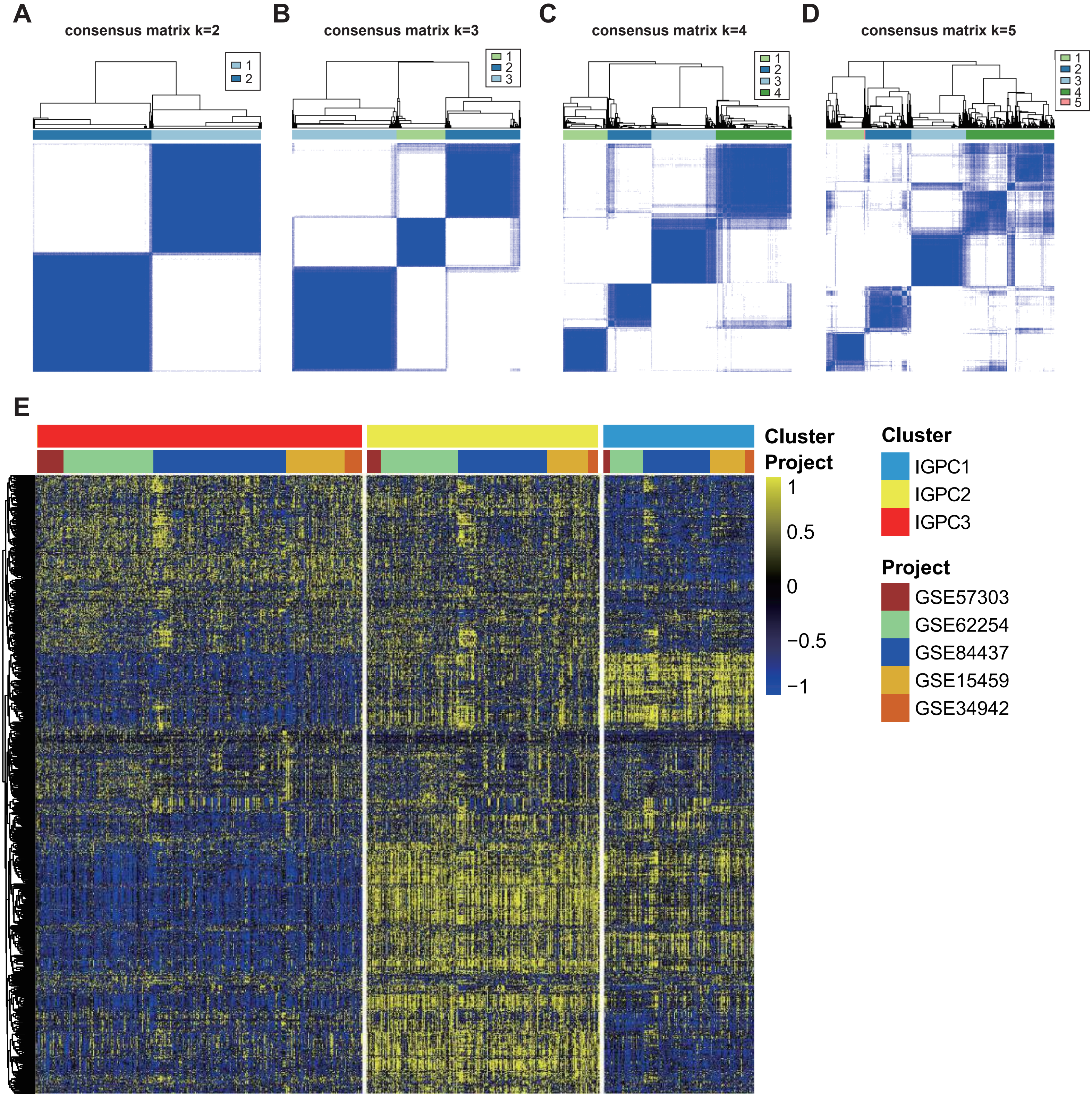

(A-D) Consensus matrices of all gastric cancer patients for k = 2, k=3, k=4 and k=5. **(E)** Gastric cancer samples were clustered into three immunogenomic phenotypes and hierarchical clustering of 730 immune-related genes. Each column represented samples; Each row represented immune-related genes.

Figure S3. Immunogenomic phenotypes of gastric cancer in the ACRG cohort, and expression of dendritic cell activating molecules in three immunogenomic phenotypes.

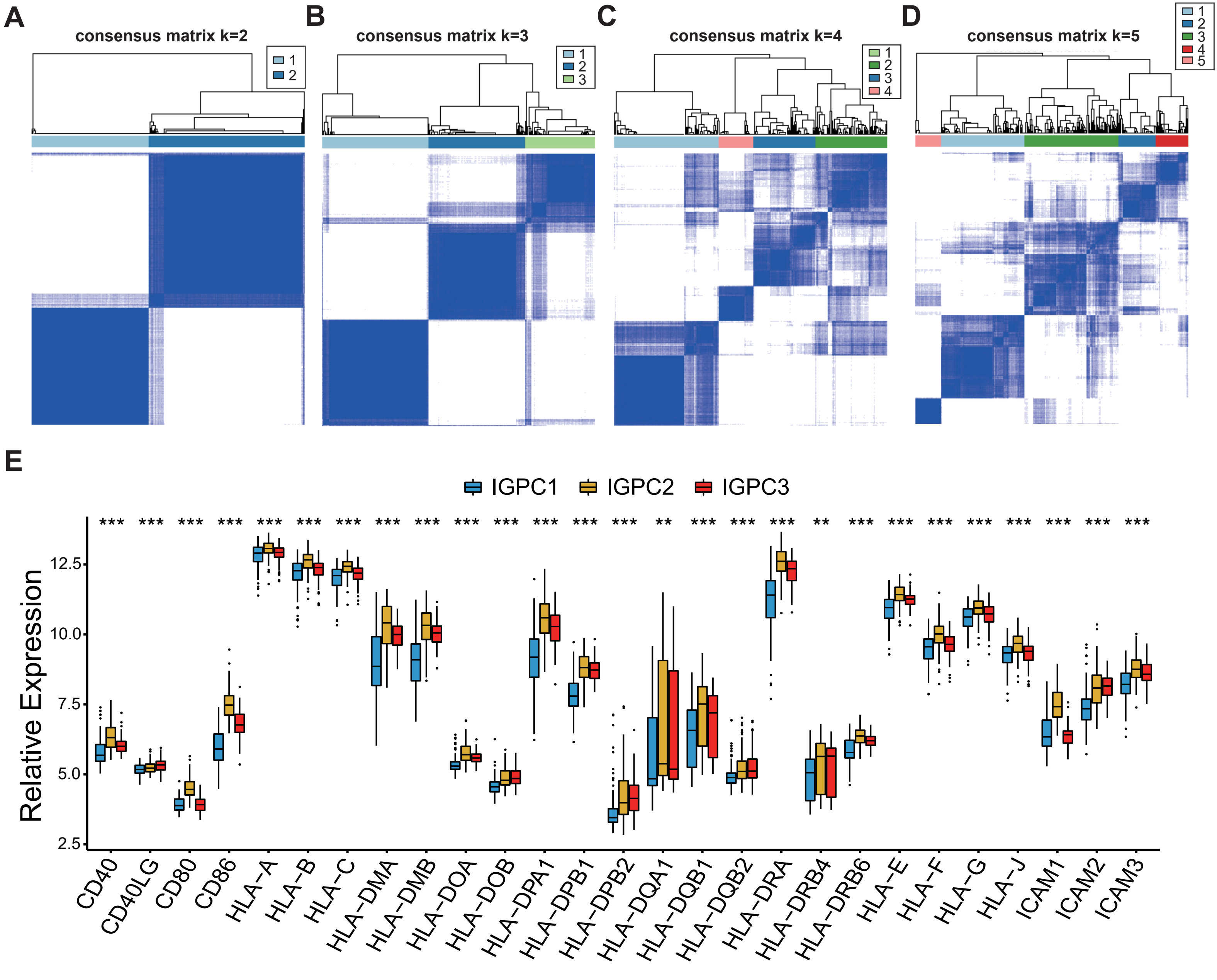

(A-D) Consensus matrices of ACRG cohorts for k = 2, k=3, k=4 and k=5. **(E)** Activation molecules of dendritic cells expressed in three immunogenomic phenotypes. The upper and lower ends of the boxes represented interquartile range of values. The lines in the boxes represented median value, and black dots showed outliers. The asterisks represented the statistical p value.(*P < 0.05; **P < 0.01; ***P < 0.001)

Figure S4. Enrichment analysis for three immunogenomic phenotypes.

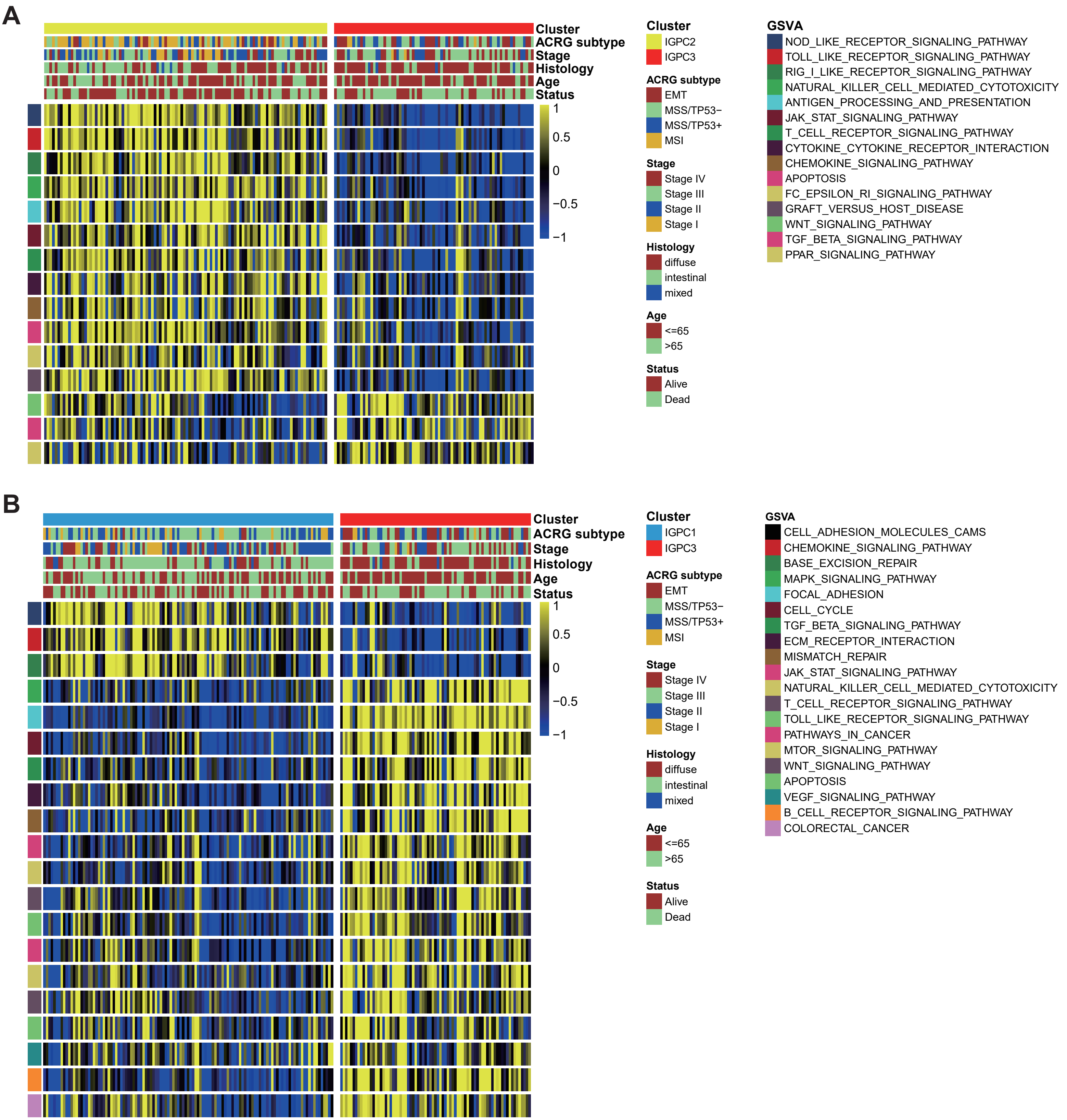

(A-B) GSVA enrichment showing the activation states of biological pathways in distinct immunogenomic phenotypes. The heatmap was used to visualize these biological processes, and yellow represented activated pathways and blue represented inhibited pathways. The cluster, ACRG subtypes, clinical stage, histology, survival status and age were used as patient annotations. **(A)** IGPC2 phenotype vs IGPC3 phenotype; **(B)** IGPC1 phenotype vs IGPC3 phenotype.

Figure S5. Correlation between IGCS signature and clinicopathological features and prognostic analyses of IGCS using multivariate Cox regression model.

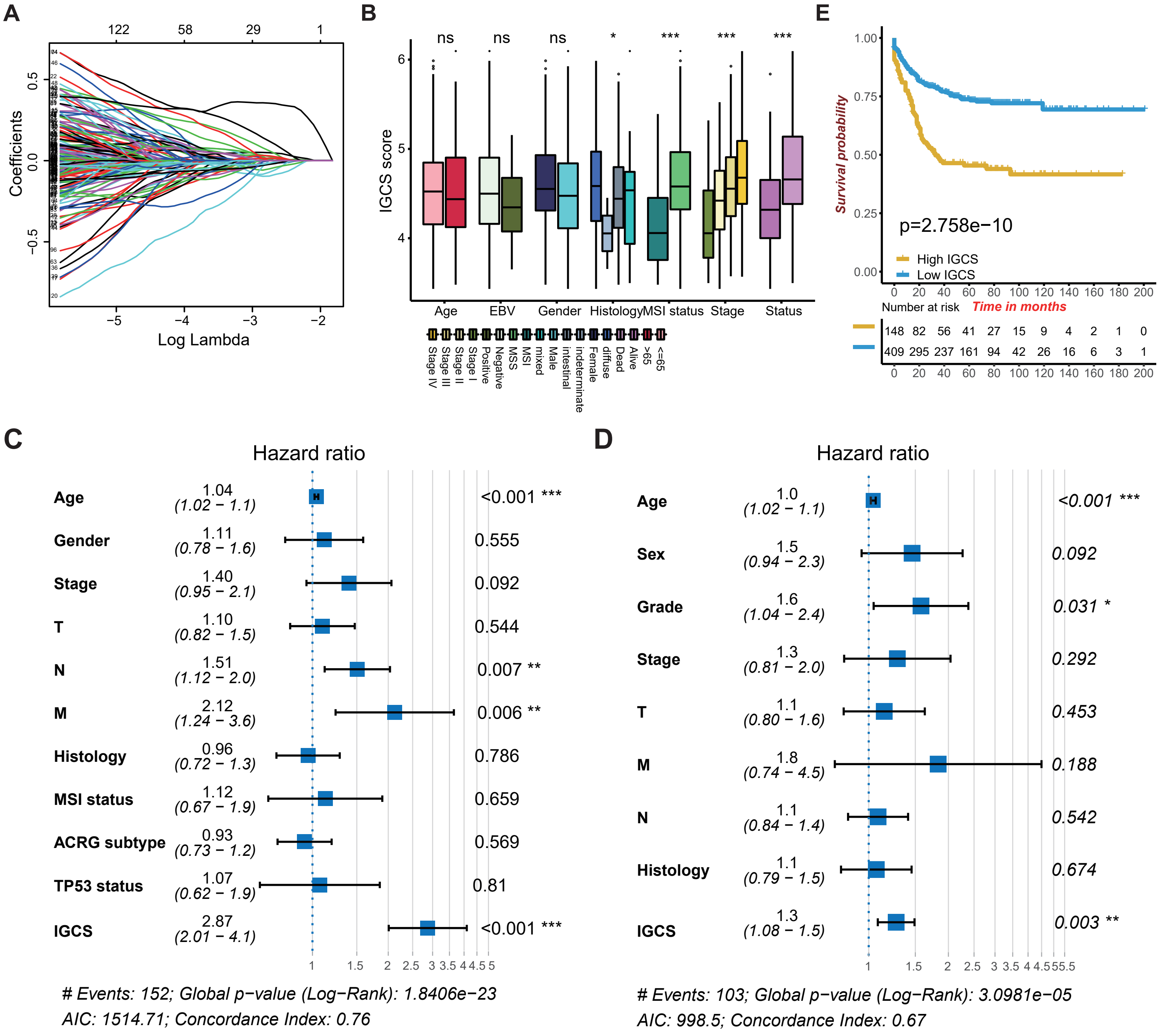

(A) Least absolute shrinkage and selection operator (LASSO) coefficient profiles of the immune-related genes. **(B)** Differences of IGCS between multiple clinicopathological features. The upper and lower ends of the boxes represented interquartile range of values. The lines in the boxes represented median value, and black dots showed outliers. The asterisks represented the statistical p value.(*P < 0.05; **P < 0.01; ***P < 0.001) **(C)** Multivariate Cox regression analyses showing the prognostic values of IGCS in the ACRG cohort. **(D)** Multivariate Cox regression analyses showing the prognostic values of IGCS in the TCGA-STAD cohort.

Figure S6. Prognostic value of IGCS in other independent gastric cancer cohorts.

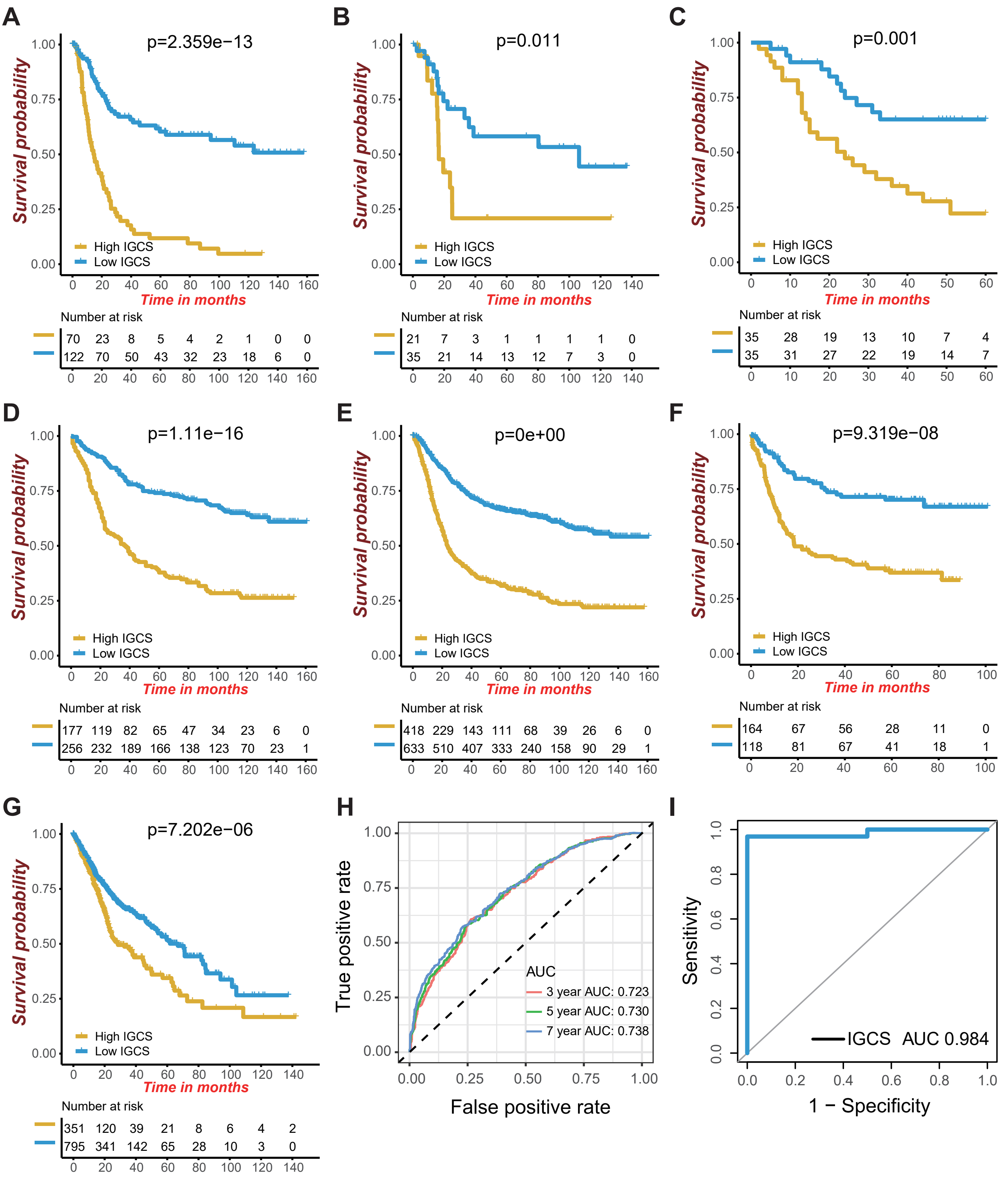

(A) GSE15459 cohort, HR 4.27; 95%CI 2.81 to 6.49; (P < 0.0001, Log-rank test) **(B)** GSE34942 cohort, HR 2.34; 95%CI 1.08 to 5.08; (P = 0.011, Log-rank test)
(C) GSE57303 cohort, HR 3.11; 95%CI 1.53 to 6.35; (P = 0.001, Log-rank test)
(D) GSE84437 cohort, HR 3.09; 95%CI 2.34 to 4.08; (P < 0.0001, Log-rank test)
(E) The combined set of all GEO gastric cancer cohorts. HR 2.88; 95%CI 2.42 to 3.43. (P < 0.0001, Log-rank test)
(F) Relapse-free survival analysis of IGCS in GSE62254 cohort. HR 2.86; 95%CI 1.91 to 4.27. (P < 0.0001, Log-rank test)
(G) Survival analysis of IGCS in all digestive cancer cohorts from TCGA HR 1.60; 95%CI 1.30 to 1.98. (P < 0.001, Log-rank test)
(H-I) The predictive values of IGCS signature in all gastric cancer patients **(H)** and patients with distant metastases **(I)**.
