# Supplementary material for: Immunogenomic characterization in gastric cancer identifies microenvironmental and immunotherapeutically relevant gene signatures
Source: Immun Inflamm Dis. 2021 Sep 28;10(1):43–59. doi: 10.1002/iid3.539 (PMC8669697; doi:10.1002/iid3.539)
Supplement: Supplementary file 2 — Supplementary information. [file IID3-10-43-s010.docx]

**Table-S1**. Basic information of gastric cancer gene expression profiles included in this study

| **Dataset** | **Platform** | **No. of  patients** | **Gender** | **Histology** | **Outcome** |
| --- | --- | --- | --- | --- | --- |
| GSE34942 | Affymetrix   GPL570 | 56 | Female:20 Male:36 | Diffuse:11;Intestinal:39;Mixed:4 | OS |
| GSE57303 | Affymetrix   GPL570 | 70 | Female:18 Male:52 | Diffuse:35;Intestinal:20;Mixed:15 | OS |
| GSE15459 | Affymetrix   GPL570 | 200 | Female:67 Male:125 | Diffuse:75;Intestinal:99;Mixed:18 | OS |
| GSE62254(ACRG) | Affymetrix  GPL570 | 300 | Female:101 Male:199 | Diffuse:135;Intestinal:146;Mixed:19 | OS/RFS |
| GSE84437 | Illumina GPL6947 | 433 | Female:137 Male:296 | NA | OS |
| TCGA-STAD | Illumina  RNAseq | 347 | Female:123 Male:224 | Diffuse:61;Intestinal:157;Mixed:117 | OS |
| GSE66229 | Affymetrix   GPL570 | 400 | NA | NA | OS/RFS |
