# Supplementary material for: Immunogenomic characterization in gastric cancer identifies microenvironmental and immunotherapeutically relevant gene signatures
Source: Immun Inflamm Dis. 2021 Sep 28;10(1):43–59. doi: 10.1002/iid3.539 (PMC8669697; doi:10.1002/iid3.539)
Supplement: Supplementary file 4 — Supplementary information. [file IID3-10-43-s001.docx]

| **Gene**  **Table-S3.** Survival analysis of immune-related genes using univariate Cox regression model. | **HR** | **HR.95L** | **HR.95H** | **pvalue** |
| --- | --- | --- | --- | --- |
| TGFB2 | 1.694556487 | 1.461292964 | 1.96505544 | 2.95E-12 |
| ITGB1 | 2.202675205 | 1.739996961 | 2.788383065 | 5.24E-11 |
| PSMB10 | 0.674901655 | 0.593582332 | 0.767361527 | 1.95E-09 |
| CASP10 | 0.368578583 | 0.264811659 | 0.513006761 | 3.29E-09 |
| JAM3 | 1.302217376 | 1.191425462 | 1.423311948 | 5.86E-09 |
| ITGA1 | 1.317140765 | 1.198422259 | 1.447619803 | 1.09E-08 |
| MFGE8 | 1.512077226 | 1.309045824 | 1.74659855 | 1.90E-08 |
| FEZ1 | 1.395735523 | 1.238576877 | 1.572835476 | 4.49E-08 |
| C7 | 1.151385636 | 1.094323388 | 1.211423331 | 5.46E-08 |
| GZMB | 0.830546597 | 0.775819907 | 0.889133733 | 9.36E-08 |
| SMPD3 | 0.674905715 | 0.58399998 | 0.779961883 | 1.00E-07 |
| PDGFC | 1.284688523 | 1.169873075 | 1.410772361 | 1.57E-07 |
| THBS1 | 1.259255831 | 1.154819815 | 1.373136508 | 1.80E-07 |
| BCL6 | 1.407587216 | 1.232688364 | 1.60730143 | 4.41E-07 |
| CASP1 | 0.820702168 | 0.758961704 | 0.887465131 | 7.35E-07 |
| PSMB8 | 0.743804647 | 0.660063936 | 0.838169339 | 1.19E-06 |
| TNFRSF11A | 0.785108972 | 0.712052464 | 0.86566107 | 1.20E-06 |
| CD38 | 0.809521472 | 0.743305859 | 0.881635742 | 1.21E-06 |
| JAK2 | 0.76175143 | 0.682370257 | 0.850367136 | 1.25E-06 |
| AXL | 1.387324557 | 1.214287752 | 1.585019221 | 1.46E-06 |
| TAP1 | 0.769585316 | 0.691602791 | 0.856360856 | 1.55E-06 |
| AKT3 | 1.525407348 | 1.282839137 | 1.813842055 | 1.76E-06 |
| PBK | 0.85031064 | 0.794802865 | 0.90969499 | 2.50E-06 |
| IKBKE | 0.647633997 | 0.540073593 | 0.776616001 | 2.76E-06 |
| PDGFRB | 1.277251946 | 1.152918614 | 1.414993664 | 2.82E-06 |
| CX3CR1 | 1.372677268 | 1.200546483 | 1.569487652 | 3.59E-06 |
| IFNG | 0.803394527 | 0.731896789 | 0.881876756 | 4.16E-06 |
| SLAMF7 | 0.783623818 | 0.706306026 | 0.869405422 | 4.22E-06 |
| PNMA1 | 1.308063435 | 1.166501598 | 1.466804635 | 4.32E-06 |
| NRP1 | 1.475380043 | 1.249154789 | 1.742575291 | 4.66E-06 |
| KLRC2 | 0.797027995 | 0.723203087 | 0.878388985 | 4.77E-06 |
| CD276 | 1.639188544 | 1.324633106 | 2.02844023 | 5.47E-06 |
| PLA2G6 | 0.370330367 | 0.241241572 | 0.568494807 | 5.56E-06 |
| KIR3DL2 | 0.446732115 | 0.315023255 | 0.633507463 | 6.15E-06 |
| IGF1R | 1.451652605 | 1.235024967 | 1.706277479 | 6.18E-06 |
| LAG3 | 0.780046606 | 0.699804737 | 0.869489266 | 7.29E-06 |
| RRAD | 1.332746599 | 1.174766107 | 1.511972031 | 8.12E-06 |
| IL12RB1 | 0.530773049 | 0.401682972 | 0.701349196 | 8.39E-06 |
| IRF1 | 0.786358136 | 0.707211407 | 0.874362477 | 8.97E-06 |
| CD244 | 0.427767333 | 0.293850944 | 0.622713301 | 9.32E-06 |
| TNFRSF14 | 0.713297752 | 0.612916652 | 0.830118877 | 1.27E-05 |
| THY1 | 1.223102736 | 1.117373937 | 1.338835866 | 1.27E-05 |
| A2M | 1.323098466 | 1.166680372 | 1.500487702 | 1.29E-05 |
| KLRD1 | 0.748592844 | 0.656839922 | 0.853162586 | 1.42E-05 |
| CTSW | 0.637177009 | 0.519721217 | 0.781177539 | 1.45E-05 |
| CD160 | 0.676362385 | 0.566413432 | 0.807654002 | 1.56E-05 |
| MEFV | 0.482637764 | 0.346323235 | 0.672606361 | 1.69E-05 |
| MCAM | 1.469382623 | 1.232341246 | 1.752019012 | 1.81E-05 |
| TAP2 | 0.714946715 | 0.612490398 | 0.834541743 | 2.12E-05 |
| C1R | 1.182722878 | 1.093679911 | 1.27901536 | 2.64E-05 |
| CXCR6 | 0.773950707 | 0.686410115 | 0.872655695 | 2.86E-05 |
| KIR3DL1 | 0.435191511 | 0.293980028 | 0.644233055 | 3.23E-05 |
| CCL20 | 0.90819378 | 0.867454665 | 0.950846166 | 3.91E-05 |
| DEFB1 | 1.107176513 | 1.054617124 | 1.162355325 | 4.08E-05 |
| IL1R1 | 1.301885011 | 1.147558001 | 1.476966376 | 4.17E-05 |
| COL3A1 | 1.220704333 | 1.108714359 | 1.344006286 | 4.87E-05 |
| PSMB9 | 0.815611292 | 0.739122245 | 0.90001591 | 4.98E-05 |
| IRF8 | 0.812834075 | 0.735131228 | 0.898750058 | 5.29E-05 |
| IL21R | 0.655617042 | 0.533468454 | 0.805734064 | 5.99E-05 |
| NLRC5 | 0.770542446 | 0.677825839 | 0.875941323 | 6.75E-05 |
| CD9 | 1.387803926 | 1.180283114 | 1.63181165 | 7.32E-05 |
| TICAM1 | 0.720360388 | 0.611849041 | 0.848116209 | 8.23E-05 |
| ITGA5 | 1.20646549 | 1.098484409 | 1.325061117 | 8.73E-05 |
| ALAS1 | 0.680261829 | 0.560974547 | 0.82491471 | 8.98E-05 |
| TNFRSF12A | 1.239559833 | 1.113090979 | 1.380398017 | 9.18E-05 |
| IL18RAP | 0.776284357 | 0.683679203 | 0.881432989 | 9.34E-05 |
| LAIR2 | 0.820587636 | 0.742967082 | 0.9063175 | 9.61E-05 |
| VEGFC | 1.244983417 | 1.114646352 | 1.390560967 | 0.000102907 |
| MEF2C | 1.24297019 | 1.11328054 | 1.387767808 | 0.000109429 |
| C8G | 0.7660714 | 0.669313511 | 0.876816887 | 0.000109656 |
| C1S | 1.161694175 | 1.076714329 | 1.253381069 | 0.00011018 |
| ENG | 1.300056593 | 1.137349565 | 1.486040174 | 0.000119798 |
| CXCR3 | 0.667384432 | 0.542765038 | 0.820616564 | 0.00012573 |
| COLEC12 | 1.133225242 | 1.062834602 | 1.208277796 | 0.000132122 |
| SYT17 | 1.24110735 | 1.110702487 | 1.386822729 | 0.000136921 |
| CD274 | 0.762762674 | 0.662825758 | 0.877767481 | 0.000157138 |
| CCL25 | 0.893091558 | 0.842174527 | 0.947086983 | 0.00015994 |
| RORA | 1.488043875 | 1.210509563 | 1.829208659 | 0.000160716 |
| CTSS | 0.807843433 | 0.722940338 | 0.902717663 | 0.000165595 |
| IL7 | 0.830101691 | 0.752944842 | 0.915165068 | 0.00018328 |
| PDCD1 | 0.639015313 | 0.504427599 | 0.809512744 | 0.000206243 |
| MNX1 | 0.710860938 | 0.593125075 | 0.851967477 | 0.000220681 |
| IFI35 | 0.787531783 | 0.692638421 | 0.895425795 | 0.000266273 |
| IFIH1 | 0.769529636 | 0.666781765 | 0.888110462 | 0.000340038 |
| GNLY | 0.852104506 | 0.780586159 | 0.93017546 | 0.000345892 |
| SPP1 | 1.107956153 | 1.047332862 | 1.172088533 | 0.000355893 |
| CD1B | 0.663869114 | 0.529888835 | 0.831725772 | 0.000368035 |
| BTLA | 0.733438804 | 0.61681394 | 0.872114658 | 0.000450445 |
| NFATC4 | 1.529348197 | 1.206160251 | 1.939133631 | 0.000452374 |
| IL2RG | 0.814913105 | 0.726805207 | 0.913701996 | 0.0004551 |
| IRF4 | 0.649871564 | 0.510012022 | 0.8280845 | 0.000491001 |
| CTLA4 | 0.733301534 | 0.615882049 | 0.873107343 | 0.000493829 |
| C1QBP | 0.795909298 | 0.699884998 | 0.905108142 | 0.000501701 |
| KIT | 1.124318141 | 1.051887432 | 1.201736273 | 0.000562946 |
| LAMP2 | 1.350080398 | 1.136433543 | 1.603892364 | 0.000637665 |
| TARP | 0.763254343 | 0.653385073 | 0.891598563 | 0.000657124 |
| FN1 | 1.247917832 | 1.098012557 | 1.418288803 | 0.000693927 |
| STAT1 | 0.838844512 | 0.757073056 | 0.929448103 | 0.000784837 |
| IL27 | 0.53526633 | 0.371449868 | 0.771328971 | 0.000799888 |
| TGFB1 | 1.682429814 | 1.241144176 | 2.280613432 | 0.000802745 |
| BCL10 | 0.707416483 | 0.577167176 | 0.86705915 | 0.000856282 |
| LAMP3 | 0.870706199 | 0.802292857 | 0.944953302 | 0.000912877 |
| ICOS | 0.79680406 | 0.696423822 | 0.911652776 | 0.000945259 |
| TNFRSF11B | 1.097956668 | 1.038509557 | 1.160806693 | 0.00100029 |
| TIGIT | 0.782500241 | 0.676113912 | 0.905626429 | 0.001003669 |
| FAS | 0.817262134 | 0.724478208 | 0.921928896 | 0.001030622 |
| PRF1 | 0.8168763 | 0.723549306 | 0.922241074 | 0.001084152 |
| IL2RB | 0.840914403 | 0.757831935 | 0.933105351 | 0.001096806 |
| SOCS1 | 0.746012665 | 0.625423242 | 0.889853237 | 0.001125071 |
| CD80 | 0.693875058 | 0.556523028 | 0.865126098 | 0.001165154 |
| CEBPB | 1.243426373 | 1.089867918 | 1.418620661 | 0.001197185 |
| TNFRSF17 | 0.910333442 | 0.859836422 | 0.963796082 | 0.001253522 |
| IRF2 | 0.712945573 | 0.580363536 | 0.875815516 | 0.00126811 |
| CDH5 | 1.178080412 | 1.066332202 | 1.301539477 | 0.001268443 |
| NCR1 | 0.57062412 | 0.405205131 | 0.80357296 | 0.001318135 |
| TRAF3 | 0.70846383 | 0.573853659 | 0.87464982 | 0.001347476 |
| IL2RA | 0.739280946 | 0.614410918 | 0.88952898 | 0.001373876 |
| CD96 | 0.828829382 | 0.738754112 | 0.929887406 | 0.00138225 |
| GZMA | 0.8866929 | 0.823486645 | 0.954750517 | 0.001436523 |
| CXCL11 | 0.915900097 | 0.867591698 | 0.966898357 | 0.001485261 |
| C2 | 0.840692245 | 0.755085909 | 0.936004025 | 0.001540468 |
| TNFRSF9 | 0.713775842 | 0.579099317 | 0.879773015 | 0.001574253 |
| CD81 | 1.307967097 | 1.107098584 | 1.545280566 | 0.001599644 |
| CCL28 | 0.871514439 | 0.799412997 | 0.950118925 | 0.001800487 |
| EGR2 | 1.158222377 | 1.055791545 | 1.270590848 | 0.001876378 |
| CASP3 | 0.78519878 | 0.674036563 | 0.914693887 | 0.001904092 |
| EWSR1 | 0.597371389 | 0.431425216 | 0.827148167 | 0.001916691 |
| RPS6 | 0.632659072 | 0.473374233 | 0.845541378 | 0.001976675 |
| F13A1 | 1.109588869 | 1.03883359 | 1.185163312 | 0.001979936 |
| IL17RB | 0.885593677 | 0.819934702 | 0.956510511 | 0.001993166 |
| KIR3DL3 | 0.591234438 | 0.421400712 | 0.829514879 | 0.002351572 |
| IFNL1 | 0.569554549 | 0.394803001 | 0.821656327 | 0.002607845 |
| MASP2 | 0.384165001 | 0.204332787 | 0.722266605 | 0.00297742 |
| TNFRSF10B | 0.775077358 | 0.654522346 | 0.91783713 | 0.003137484 |
| THBD | 1.19626388 | 1.061947942 | 1.347568194 | 0.003187098 |
| CCL1 | 0.539553836 | 0.35776125 | 0.813722396 | 0.003247609 |
| CXCL9 | 0.923684007 | 0.876086294 | 0.9738677 | 0.003272074 |
| SH2D1B | 0.662590196 | 0.503630946 | 0.871721192 | 0.00327293 |
| BLNK | 0.859017383 | 0.775968279 | 0.950954936 | 0.003396815 |
| CXCL13 | 0.933416953 | 0.891313759 | 0.977508984 | 0.003434227 |
| CD8B | 0.584266855 | 0.406638955 | 0.83948612 | 0.003659139 |
| ENTPD1 | 1.19051849 | 1.058258196 | 1.339308573 | 0.003703545 |
| TLR8 | 0.841345261 | 0.748656928 | 0.94550898 | 0.003721613 |
| IL18R1 | 0.849195706 | 0.760311361 | 0.948471091 | 0.003757773 |
| SLAMF1 | 0.762280057 | 0.634287984 | 0.91609947 | 0.003799317 |
| CD7 | 0.826258701 | 0.725665967 | 0.940795726 | 0.003959512 |
| HLA-DOB | 0.831131473 | 0.7325554 | 0.942972402 | 0.004084761 |
| PRPF38A | 0.696860052 | 0.544593443 | 0.891699925 | 0.004089098 |
| IL1RAP | 1.290047043 | 1.083244657 | 1.536330101 | 0.00427726 |
| TNFSF13 | 0.814325176 | 0.707218268 | 0.937653228 | 0.004308057 |
| TLR3 | 0.848384273 | 0.757817657 | 0.949774484 | 0.004308857 |
| ANXA1 | 1.145602228 | 1.043458724 | 1.257744494 | 0.00433402 |
| IL6ST | 1.237023302 | 1.068825862 | 1.431689392 | 0.004336284 |
| MTMR14 | 0.63790819 | 0.467420427 | 0.870579964 | 0.004603984 |
| F12 | 0.872713155 | 0.794276675 | 0.9588954 | 0.004604008 |
| SERPING1 | 1.125433759 | 1.036743816 | 1.221710827 | 0.004778697 |
| ICAM3 | 0.826640792 | 0.724239122 | 0.943521246 | 0.004779031 |
| IL32 | 0.874142476 | 0.796010168 | 0.959943853 | 0.004867029 |
| CCL2 | 1.106050164 | 1.030461696 | 1.187183348 | 0.005258009 |
| LYN | 0.827805076 | 0.724620858 | 0.945682471 | 0.005399511 |
| CD59 | 1.213524148 | 1.058612171 | 1.391105163 | 0.005479567 |
| TTK | 0.900847538 | 0.836845482 | 0.96974448 | 0.005485733 |
| SH2D1A | 0.807490598 | 0.693926784 | 0.939639572 | 0.005691131 |
| CD247 | 0.831824518 | 0.729912706 | 0.947965453 | 0.005756749 |
| SBNO2 | 0.778956493 | 0.652359965 | 0.930120257 | 0.005771246 |
| TREM2 | 1.194300579 | 1.051777428 | 1.356136607 | 0.006171096 |
| CASP8 | 0.674761878 | 0.509036862 | 0.894441299 | 0.006223919 |
| MICA | 1.249198731 | 1.064557603 | 1.465864755 | 0.006399549 |
| CARD11 | 1.241263221 | 1.062495958 | 1.450108467 | 0.006449611 |
| EGR1 | 1.138615213 | 1.036920569 | 1.250283428 | 0.006538308 |
| IL12A | 0.717329557 | 0.56429731 | 0.911862744 | 0.006655682 |
| CD3D | 0.872068004 | 0.789400002 | 0.963393213 | 0.007062482 |
| CXCL12 | 1.093757679 | 1.024603509 | 1.167579312 | 0.007159261 |
| LY86 | 1.15119779 | 1.038850862 | 1.27569452 | 0.007199902 |
| APOE | 1.112491029 | 1.029178293 | 1.202547991 | 0.007271833 |
| PTGDR2 | 0.706661901 | 0.548352351 | 0.910675482 | 0.007296112 |
| CD6 | 0.814975288 | 0.70022803 | 0.948526325 | 0.008229694 |
| MYD88 | 0.776207307 | 0.642404389 | 0.937879307 | 0.008681799 |
| LRP1 | 1.367389921 | 1.082089963 | 1.727911042 | 0.008773672 |
| TLR7 | 1.169741038 | 1.040233354 | 1.31537226 | 0.008822896 |
| SLAMF6 | 0.845862723 | 0.746014498 | 0.959074855 | 0.009002311 |
| OAS3 | 0.851439462 | 0.754409914 | 0.960948609 | 0.009180924 |
| CLU | 1.14256441 | 1.03352802 | 1.263104054 | 0.009203232 |
| HLA-DMA | 0.896107524 | 0.825101404 | 0.973224249 | 0.009205463 |
| POU2AF1 | 0.878801995 | 0.796941617 | 0.969070922 | 0.009605195 |
| CD3G | 0.848312372 | 0.748909854 | 0.960908548 | 0.009679777 |
| MICB | 0.883334936 | 0.804089413 | 0.970390352 | 0.009689704 |
| CD36 | 1.143592267 | 1.032430088 | 1.266723325 | 0.010120816 |
| YTHDF2 | 0.738540855 | 0.586071011 | 0.930676631 | 0.010201824 |
| SDHA | 0.786608197 | 0.654716426 | 0.945069394 | 0.010367781 |
| TIRAP | 0.610452973 | 0.418269436 | 0.890939669 | 0.010509119 |
| CLEC4C | 0.432128247 | 0.227143337 | 0.822101251 | 0.010559702 |
| IFI27 | 0.894848043 | 0.821023402 | 0.975310835 | 0.011438072 |
| LY9 | 0.780422919 | 0.643940723 | 0.945832296 | 0.011478706 |
| MAF | 1.196049351 | 1.040974544 | 1.374225776 | 0.011512509 |
| CD5 | 0.770072558 | 0.627586144 | 0.944908917 | 0.012321777 |
| BST2 | 0.902854162 | 0.83280727 | 0.978792655 | 0.013131289 |
| CDK1 | 0.895838503 | 0.821218178 | 0.977239234 | 0.013181695 |
| ABL1 | 1.225623341 | 1.043155031 | 1.440008943 | 0.013373593 |
| CD74 | 0.870988089 | 0.780580856 | 0.97186633 | 0.013498673 |
| CD99 | 1.18985567 | 1.035807542 | 1.366814255 | 0.013999072 |
| MERTK | 1.259373315 | 1.047359067 | 1.514305073 | 0.014209037 |
| KLRB1 | 0.905615563 | 0.836468477 | 0.980478727 | 0.014427227 |
| TYK2 | 0.779826792 | 0.638836018 | 0.951934156 | 0.014521741 |
| TLR5 | 1.174671755 | 1.031723638 | 1.337425724 | 0.015028201 |
| NEFL | 1.214010149 | 1.037887122 | 1.420020166 | 0.01530985 |
| CNOT10 | 0.794399772 | 0.659294032 | 0.957192038 | 0.015522287 |
| PDCD1LG2 | 0.722998143 | 0.55465846 | 0.942429173 | 0.016465882 |
| TNFSF10 | 0.893071301 | 0.814037175 | 0.979778777 | 0.016753669 |
| RAG1 | 0.564427207 | 0.353096264 | 0.902241412 | 0.016857139 |
| NFKB1 | 0.796652022 | 0.660967414 | 0.960190217 | 0.017014236 |
| ICAM2 | 1.148312051 | 1.025019757 | 1.286434294 | 0.01701452 |
| AMMECR1L | 1.292671806 | 1.045801156 | 1.597818466 | 0.017590316 |
| EP300 | 1.232204419 | 1.036956429 | 1.464215554 | 0.01767972 |
| ECSIT | 0.779631529 | 0.634666475 | 0.957708253 | 0.017708543 |
| ITGA6 | 0.859619614 | 0.758534096 | 0.974176222 | 0.017794914 |
| HLA-G | 0.857960611 | 0.755770442 | 0.973968244 | 0.017903675 |
| PPIA | 0.72920456 | 0.561448158 | 0.947085288 | 0.017906585 |
| CCL19 | 1.061984477 | 1.010032355 | 1.116608813 | 0.018771337 |
| ICOSLG | 0.728369392 | 0.557402782 | 0.951774889 | 0.020228375 |
| ELK1 | 0.744069769 | 0.579427568 | 0.955494444 | 0.020517382 |
| CXCL1 | 0.932119008 | 0.878260101 | 0.989280789 | 0.020620518 |
| FCER2 | 0.788440147 | 0.643823742 | 0.965540451 | 0.021496188 |
| CYFIP2 | 1.133351701 | 1.018474443 | 1.261186362 | 0.021694349 |
| IL23R | 0.565303772 | 0.346802584 | 0.921470511 | 0.022135875 |
| CCR4 | 0.699416951 | 0.514726945 | 0.950375876 | 0.02229405 |
| IGLL1 | 0.875539391 | 0.780684161 | 0.981919787 | 0.02309714 |
| BIRC5 | 0.889528087 | 0.803896402 | 0.984281327 | 0.023405717 |
| CCL18 | 0.921990095 | 0.859397745 | 0.989141222 | 0.023552534 |
| IFITM1 | 0.908743114 | 0.836489869 | 0.987237356 | 0.023584117 |
| MST1R | 0.901927897 | 0.824682893 | 0.986408155 | 0.023850166 |
| CTSL | 1.134734086 | 1.016214463 | 1.267076482 | 0.024720786 |
| BST1 | 1.145277941 | 1.017045286 | 1.28967862 | 0.025160142 |
| CHUK | 0.821752883 | 0.691730348 | 0.976215374 | 0.025490936 |
| CD164 | 0.869777022 | 0.769254686 | 0.983435112 | 0.025978567 |
| SPACA3 | 0.691338655 | 0.499169849 | 0.957487991 | 0.026323967 |
| CCL5 | 0.922999542 | 0.859598534 | 0.991076789 | 0.027326062 |
| BATF | 0.871583093 | 0.771026411 | 0.9852543 | 0.027986479 |
| HLA-DRA | 0.911416916 | 0.838533405 | 0.990635303 | 0.029167021 |
| TP53 | 0.870859812 | 0.768994466 | 0.986218817 | 0.029360946 |
| FCGR1A | 0.792196984 | 0.642174804 | 0.977266715 | 0.029657262 |
| IFNA7 | 0.62579474 | 0.410006056 | 0.955154324 | 0.029807849 |
| TFRC | 0.830002864 | 0.700807538 | 0.983015618 | 0.030896671 |
| C3 | 1.081883901 | 1.007027086 | 1.162305156 | 0.031446349 |
| ATG10 | 0.814028088 | 0.674881852 | 0.98186331 | 0.0314499 |
| TNFRSF1A | 1.223700066 | 1.017454088 | 1.471753732 | 0.032056463 |
| CD34 | 1.149466412 | 1.011846681 | 1.305803595 | 0.032276091 |
| CSF1R | 1.131837741 | 1.010414483 | 1.267852643 | 0.032443457 |
| ISG20 | 0.90515795 | 0.825888571 | 0.992035661 | 0.033091903 |
| FCER1A | 1.095082374 | 1.007259825 | 1.190562133 | 0.033207909 |
| IL5RA | 0.592175098 | 0.364729456 | 0.961456063 | 0.034096937 |
| HLA-DPB1 | 0.891870511 | 0.80226948 | 0.991478585 | 0.034142324 |
| TNFSF13B | 0.913515582 | 0.840031072 | 0.993428395 | 0.034510258 |
| FLT3 | 0.728087574 | 0.5424551 | 0.977244966 | 0.034579441 |
| PLAU | 1.096325267 | 1.006287568 | 1.1944191 | 0.035438027 |
| CRP | 1.465228714 | 1.024559977 | 2.095431435 | 0.036358719 |
| CD8A | 0.924350035 | 0.858526053 | 0.995220801 | 0.036882163 |
| CDH1 | 0.9294885 | 0.86739467 | 0.996027415 | 0.038190751 |
| IL4 | 0.617708832 | 0.391422965 | 0.974813018 | 0.038494167 |
| NFATC2 | 1.375152665 | 1.016575905 | 1.860210186 | 0.038770463 |
| MAP2K2 | 0.806306721 | 0.657302527 | 0.989088741 | 0.038903434 |
| IFNA2 | 0.555424825 | 0.3177297 | 0.970940821 | 0.039070133 |
| IFNL2 | 0.741068005 | 0.557376749 | 0.985297268 | 0.039219199 |
| G6PD | 1.141905526 | 1.006556754 | 1.295454255 | 0.039256147 |
| CD209 | 0.835234095 | 0.703541193 | 0.991578034 | 0.039727354 |
| HPRT1 | 0.799842413 | 0.646405156 | 0.989701088 | 0.039856967 |
| MAP4K2 | 1.183145351 | 1.00759435 | 1.389282227 | 0.04014157 |
| ITGAL | 0.88676426 | 0.790538189 | 0.994703182 | 0.04030741 |
| CAMP | 0.786187728 | 0.624101989 | 0.990368808 | 0.041139651 |
| CD2 | 0.915635959 | 0.840833533 | 0.997092976 | 0.042671047 |
| GTF3C1 | 1.229770957 | 1.006799884 | 1.50212235 | 0.042727997 |
| HLA-DRB4 | 0.919181752 | 0.846970608 | 0.997549484 | 0.043514392 |
| PLA2G1B | 1.180727491 | 1.004000189 | 1.388562895 | 0.04461964 |
| GPATCH3 | 0.764758293 | 0.588560656 | 0.993704287 | 0.044725032 |
| CXCR1 | 0.806078027 | 0.652856787 | 0.995259296 | 0.045054866 |
| CCL23 | 0.858784139 | 0.738940234 | 0.998064745 | 0.047119474 |
| PRKCD | 0.866863276 | 0.752747534 | 0.998278845 | 0.0472688 |
| CD27 | 0.9071495 | 0.823502623 | 0.999292769 | 0.048347918 |
| ATM | 1.251330055 | 1.001411461 | 1.56361992 | 0.048567157 |
| REL | 0.804959832 | 0.648695541 | 0.998866633 | 0.048808347 |
| IL6 | 1.060767333 | 1.000258983 | 1.124935995 | 0.048998442 |
| TNFRSF18 | 0.807298235 | 0.652199553 | 0.999280724 | 0.049232308 |
| TUBB | 1.190455079 | 1.000500011 | 1.41647504 | 0.049344822 |
| HLA-E | 0.851839393 | 0.725948945 | 0.999561135 | 0.049374447 |
| CXCL2 | 0.909334241 | 0.826903347 | 0.999982363 | 0.049957493 |
| CXCL3 | 0.930361115 | 0.865476635 | 1.000109961 | 0.050349476 |
| CCR2 | 0.86534533 | 0.748368579 | 1.000606601 | 0.050964306 |
| PECAM1 | 1.123843474 | 0.999018978 | 1.264264426 | 0.051940714 |
| CXCL10 | 0.946325771 | 0.894990221 | 1.000605866 | 0.052540363 |
| GZMH | 0.925748615 | 0.856193059 | 1.000954737 | 0.052865515 |
| ITGB3 | 1.531800658 | 0.992515557 | 2.364107281 | 0.054100207 |
| CREB5 | 1.208458976 | 0.996619371 | 1.465326823 | 0.054163472 |
| MSR1 | 1.14475212 | 0.996678422 | 1.31482471 | 0.05576264 |
| APP | 1.159462854 | 0.995931083 | 1.349846524 | 0.05646854 |
| TPSAB1 | 1.077228948 | 0.997825299 | 1.162951278 | 0.056879352 |
| PTGS2 | 1.055594798 | 0.997676734 | 1.116875176 | 0.060220272 |
| IFNAR1 | 0.80403203 | 0.6401871 | 1.009810265 | 0.06065556 |
| LCP1 | 0.910960098 | 0.82635484 | 1.004227554 | 0.060773727 |
| CD14 | 1.09934302 | 0.995547634 | 1.213960069 | 0.061237479 |
| HLA-DPA1 | 0.931523752 | 0.864502083 | 1.003741364 | 0.062611554 |
| LTK | 0.81079049 | 0.650075929 | 1.011237595 | 0.062769283 |
| NT5E | 1.098640951 | 0.994371844 | 1.213843641 | 0.064453525 |
| IL4R | 1.173932345 | 0.99021617 | 1.391733636 | 0.064785396 |
| IL10 | 1.12514595 | 0.992439618 | 1.275597412 | 0.065556399 |
| CD79A | 0.920744942 | 0.842773805 | 1.005929756 | 0.067398776 |
| PRAME | 1.076667979 | 0.994410967 | 1.165729236 | 0.068493626 |
| TMEFF2 | 1.169132704 | 0.987962833 | 1.383525003 | 0.068912439 |
| LRRN3 | 1.128140559 | 0.989351574 | 1.286399248 | 0.071840155 |
| IL26 | 0.84383721 | 0.701202288 | 1.015486185 | 0.072288316 |
| HLA-B | 0.881610882 | 0.768076974 | 1.011926895 | 0.073228674 |
| CCL27 | 0.68840276 | 0.45718117 | 1.036565788 | 0.073777799 |
| CD33 | 0.84283819 | 0.698191541 | 1.017451764 | 0.075098474 |
| ZC3H14 | 0.757020226 | 0.556916237 | 1.02902301 | 0.075519383 |
| DHX16 | 1.221591021 | 0.97934538 | 1.523757249 | 0.075916237 |
| CEACAM6 | 1.036585063 | 0.996230235 | 1.078574566 | 0.076138972 |
| CD4 | 0.828918565 | 0.673547958 | 1.02012927 | 0.076431754 |
| ERCC3 | 1.222561275 | 0.978705274 | 1.527176885 | 0.076671654 |
| AICDA | 0.771587953 | 0.578940346 | 1.028340784 | 0.076847246 |
| STAT5B | 1.215632082 | 0.978588424 | 1.510094869 | 0.077666413 |
| TNFRSF4 | 0.81917468 | 0.656314416 | 1.022447688 | 0.077787635 |
| PPBP | 1.050705627 | 0.994367618 | 1.110235585 | 0.078563001 |
| C8B | 1.27482123 | 0.972498568 | 1.671127568 | 0.07873916 |
| STAT2 | 0.844269188 | 0.698468774 | 1.020504406 | 0.080096714 |
| RELA | 1.20186049 | 0.978053309 | 1.476881294 | 0.080309584 |
| HLA-A | 0.834969125 | 0.681996947 | 1.022253021 | 0.080671191 |
| IL15RA | 0.854291388 | 0.715523259 | 1.019972121 | 0.081629892 |
| TAPBP | 0.858047105 | 0.722073183 | 1.019626335 | 0.082005025 |
| IL34 | 1.219429915 | 0.975110725 | 1.524964581 | 0.082030507 |
| ATF2 | 0.801911681 | 0.625239646 | 1.028505386 | 0.082104189 |
| REPS1 | 0.747856022 | 0.537110002 | 1.041292523 | 0.085364173 |
| XCR1 | 0.658490991 | 0.408833726 | 1.060603267 | 0.085792723 |
| CCL17 | 0.786148895 | 0.59731178 | 1.034685915 | 0.08603691 |
| IL1RN | 0.907343429 | 0.81115935 | 1.014932638 | 0.088997406 |
| HLA-DMB | 0.922617809 | 0.840825833 | 1.012366163 | 0.089041811 |
| IRF5 | 1.208950121 | 0.970455109 | 1.506056674 | 0.09055622 |
| PSEN1 | 0.742961699 | 0.525817479 | 1.049778884 | 0.092078902 |
| POLR2A | 0.814922987 | 0.642220274 | 1.034068063 | 0.092129552 |
| DMBT1 | 0.969280727 | 0.934699693 | 1.005141153 | 0.092315647 |
| LY96 | 1.075425411 | 0.987255339 | 1.1714698 | 0.095697325 |
| CFB | 0.920545135 | 0.834999174 | 1.01485531 | 0.096183738 |
| NCF4 | 0.892284581 | 0.779906577 | 1.020855314 | 0.097029307 |
| LTA | 0.846847516 | 0.695512053 | 1.031111843 | 0.097934579 |
| FPR2 | 0.892684562 | 0.78037396 | 1.021158788 | 0.097973314 |
| CD3E | 0.883365589 | 0.762564611 | 1.023303145 | 0.098344213 |
| MAP2K4 | 0.855929102 | 0.711519973 | 1.02964731 | 0.098928044 |
| CEACAM8 | 0.739922094 | 0.516700447 | 1.05957854 | 0.100158177 |
| DUSP6 | 1.116977561 | 0.978840843 | 1.274608513 | 0.100497343 |
| CCR3 | 0.859096457 | 0.716678459 | 1.02981569 | 0.100534242 |
| ABCB1 | 1.09909577 | 0.981612097 | 1.230640408 | 0.101381233 |
| AIRE | 0.852414961 | 0.70403186 | 1.032071568 | 0.101745629 |
| CD79B | 0.908402326 | 0.809456669 | 1.019442817 | 0.102532225 |
| CFP | 0.827672192 | 0.659476578 | 1.038765106 | 0.102715408 |
| CXCR4 | 1.068999659 | 0.986648845 | 1.158223897 | 0.10281885 |
| COG7 | 0.837880436 | 0.677418691 | 1.036351129 | 0.102939365 |
| LILRA4 | 0.859094914 | 0.715328384 | 1.031755607 | 0.104078254 |
| CTSH | 1.09662237 | 0.980743454 | 1.226192861 | 0.105508271 |
| LCK | 0.891281354 | 0.77485498 | 1.025201454 | 0.107074416 |
| FOXP3 | 0.764226729 | 0.550792688 | 1.060367186 | 0.10757693 |
| SPA17 | 0.897145131 | 0.78546221 | 1.024707969 | 0.109569591 |
| TXNIP | 1.084500079 | 0.98145333 | 1.198366123 | 0.111282621 |
| IFNA17 | 0.729915567 | 0.49507666 | 1.076149973 | 0.111959926 |
| SELE | 1.055684088 | 0.986836949 | 1.129334379 | 0.11528607 |
| BCL2L1 | 1.122765071 | 0.971762723 | 1.297231694 | 0.116117128 |
| CXCL16 | 0.917522333 | 0.823895782 | 1.021788496 | 0.117006662 |
| CCL22 | 0.903381315 | 0.795544342 | 1.025835717 | 0.117190376 |
| BTK | 0.905563889 | 0.799323841 | 1.025924556 | 0.119237011 |
| MAGEA12 | 1.031691152 | 0.991981859 | 1.072990018 | 0.119242911 |
| ADA | 0.915347161 | 0.818750133 | 1.023340812 | 0.120070064 |
| HAMP | 1.123106571 | 0.969942093 | 1.300457398 | 0.120664361 |
| TNF | 0.879424545 | 0.747698447 | 1.034357545 | 0.120675592 |
| SPN | 0.879819715 | 0.748429008 | 1.03427676 | 0.12076748 |
| SSX1 | 1.120990704 | 0.969945195 | 1.295557899 | 0.121933251 |
| TFE3 | 1.246755296 | 0.942383873 | 1.649432691 | 0.122490045 |
| CEACAM1 | 0.929946462 | 0.847631402 | 1.020255291 | 0.124563412 |
| LAMP1 | 1.159036866 | 0.959934002 | 1.399436266 | 0.124845256 |
| NOL7 | 0.821405631 | 0.6388388 | 1.056146263 | 0.125023715 |
| S100B | 1.185651679 | 0.953682198 | 1.474044399 | 0.125268397 |
| MAVS | 0.857337648 | 0.703554502 | 1.04473476 | 0.126991168 |
| HSD11B1 | 1.063590678 | 0.982526357 | 1.151343292 | 0.127470244 |
| IL1R2 | 0.939330612 | 0.866394789 | 1.0184064 | 0.129093455 |
| ITGA2 | 1.082141727 | 0.976963486 | 1.19864328 | 0.130224214 |
| CSF2RB | 0.921040777 | 0.827941216 | 1.024609109 | 0.130326383 |
| ALCAM | 1.089719237 | 0.974592635 | 1.218445504 | 0.131501037 |
| CXCL5 | 0.966183474 | 0.923548455 | 1.010786712 | 0.135170858 |
| IL24 | 1.058705446 | 0.981790207 | 1.141646365 | 0.138232003 |
| SMAD2 | 0.795368568 | 0.587598619 | 1.076604231 | 0.138304686 |
| GUSB | 0.835451238 | 0.658659145 | 1.059696469 | 0.138340462 |
| TLR9 | 0.799200291 | 0.593912057 | 1.075447277 | 0.138935588 |
| IL22RA1 | 0.928977951 | 0.842580323 | 1.024234735 | 0.139093753 |
| C3AR1 | 1.090366529 | 0.972252012 | 1.222830246 | 0.139162081 |
| FADD | 1.128462505 | 0.961213491 | 1.324812476 | 0.139774453 |
| FCGR2A | 1.102819838 | 0.968242572 | 1.25610217 | 0.14049892 |
| OSM | 0.920341419 | 0.823997227 | 1.027950459 | 0.141197813 |
| ITCH | 1.157104234 | 0.951544009 | 1.407071239 | 0.143675941 |
| LILRA5 | 0.873602911 | 0.728074137 | 1.048220238 | 0.146104118 |
| ILF3 | 0.850870814 | 0.683550452 | 1.05914807 | 0.148294594 |
| ABCF1 | 1.146863244 | 0.952362101 | 1.381087403 | 0.148404336 |
| CCL13 | 0.907793641 | 0.796094534 | 1.035165122 | 0.148723102 |
| CXCR2 | 0.933768434 | 0.850751839 | 1.02488581 | 0.149156018 |
| TFEB | 1.170681666 | 0.944521052 | 1.450995253 | 0.15020003 |
| IL22RA2 | 0.832985613 | 0.649283901 | 1.068661998 | 0.150560173 |
| IL2 | 0.68317868 | 0.40595391 | 1.149719455 | 0.151395306 |
| MAPK1 | 0.834516778 | 0.651667792 | 1.068670664 | 0.151678067 |
| CCL21 | 1.052941667 | 0.981205096 | 1.129922949 | 0.151876006 |
| CXCL6 | 1.037728763 | 0.986302683 | 1.091836213 | 0.153257729 |
| TRAF2 | 0.885148111 | 0.748145115 | 1.047239583 | 0.15503408 |
| FCGR2B | 1.068824946 | 0.974988376 | 1.171692702 | 0.155697321 |
| TLR10 | 0.887592973 | 0.752858832 | 1.046439588 | 0.1557358 |
| TRAF6 | 0.809915265 | 0.604316552 | 1.085462137 | 0.158218977 |
| CDKN1A | 1.09792702 | 0.964028273 | 1.25042364 | 0.159165182 |
| DUSP4 | 0.935064111 | 0.850714832 | 1.02777671 | 0.163937516 |
| INPP5D | 0.879368341 | 0.733319108 | 1.054505018 | 0.1653659 |
| UBC | 1.265714029 | 0.907005776 | 1.76628644 | 0.165779654 |
| ZAP70 | 0.866983337 | 0.708419183 | 1.061038611 | 0.166038906 |
| NLRP3 | 1.15134561 | 0.942547017 | 1.406398501 | 0.167461685 |
| IFNB1 | 0.821861764 | 0.621613105 | 1.086619237 | 0.168536008 |
| MRC1 | 1.075064046 | 0.969223197 | 1.192462898 | 0.171063567 |
| FUT7 | 0.793088579 | 0.56875973 | 1.105896676 | 0.171753819 |
| HLA-C | 0.90486269 | 0.783774002 | 1.044658901 | 0.172598198 |
| MAPK3 | 0.883282681 | 0.738607406 | 1.056296332 | 0.173872373 |
| TLR6 | 0.829597116 | 0.633649278 | 1.086139288 | 0.174175174 |
| IL1B | 0.957481475 | 0.899209101 | 1.019530135 | 0.175028239 |
| CLEC4A | 0.93312089 | 0.844200444 | 1.031407412 | 0.175501855 |
| RIPK2 | 0.910848377 | 0.795416901 | 1.043031351 | 0.17682537 |
| LTB | 0.93018058 | 0.837012969 | 1.033718644 | 0.17891542 |
| NOD1 | 1.145838319 | 0.939497394 | 1.397497706 | 0.178987737 |
| IL1RAPL2 | 0.779267429 | 0.541568306 | 1.121294801 | 0.179165839 |
| ITK | 0.939423133 | 0.857077939 | 1.029679778 | 0.181849024 |
| MAGEC1 | 0.901115581 | 0.772524127 | 1.051111883 | 0.185032798 |
| CLEC5A | 1.060506245 | 0.972040425 | 1.157023379 | 0.186210391 |
| LILRB2 | 0.909038364 | 0.787732285 | 1.049024855 | 0.191883439 |
| CD58 | 1.120065775 | 0.944324845 | 1.328512478 | 0.192876951 |
| HMGB1 | 0.894326828 | 0.755803087 | 1.058239228 | 0.193358598 |
| IL25 | 0.799641404 | 0.570462615 | 1.120890937 | 0.19441249 |
| IL15 | 0.921826373 | 0.813390761 | 1.044717868 | 0.202372293 |
| ICAM4 | 0.884838971 | 0.732801137 | 1.068420839 | 0.203393869 |
| CCR1 | 0.927937934 | 0.826814181 | 1.041429658 | 0.203937215 |
| TNFSF4 | 1.063515355 | 0.9670497 | 1.169603703 | 0.204324506 |
| USP39 | 0.868297078 | 0.697409799 | 1.0810571 | 0.206607551 |
| CCR5 | 0.91644012 | 0.800212095 | 1.049549862 | 0.207290527 |
| IL3 | 0.75648487 | 0.49009365 | 1.167673481 | 0.207649315 |
| ATG7 | 0.849579738 | 0.658048768 | 1.096857508 | 0.211053561 |
| CD40LG | 0.804892737 | 0.572538566 | 1.131543544 | 0.211710908 |
| STAT4 | 0.926385196 | 0.821570662 | 1.044571782 | 0.211973511 |
| F2RL1 | 0.953236079 | 0.883930918 | 1.027975154 | 0.213664235 |
| MAP2K1 | 1.125540149 | 0.93378691 | 1.356669936 | 0.214586621 |
| LIF | 1.071265552 | 0.96071963 | 1.194531523 | 0.215407918 |
| MRPS5 | 0.853522172 | 0.664150037 | 1.09689085 | 0.215926716 |
| ANP32B | 0.858963971 | 0.674950815 | 1.093144991 | 0.216479734 |
| IL12B | 0.771134512 | 0.51061538 | 1.16457212 | 0.216599659 |
| TBX21 | 0.910481816 | 0.78342665 | 1.058142632 | 0.221341093 |
| SELL | 0.949742126 | 0.873763222 | 1.032327847 | 0.22547913 |
| SYCP1 | 0.714691711 | 0.41460294 | 1.231984127 | 0.22664662 |
| LBP | 1.080692493 | 0.951708832 | 1.227157115 | 0.23142717 |
| IL1A | 1.07403338 | 0.95502814 | 1.20786776 | 0.233262706 |
| TNFRSF10C | 0.853293722 | 0.657301809 | 1.107725807 | 0.233432334 |
| ETS1 | 1.090997109 | 0.944380218 | 1.260376562 | 0.236895564 |
| CD70 | 0.905924638 | 0.768895602 | 1.067374358 | 0.237705191 |
| FYN | 1.10053413 | 0.937292448 | 1.292206476 | 0.242236715 |
| MPPED1 | 0.806738367 | 0.561634479 | 1.158808472 | 0.245126012 |
| DOCK9 | 1.148163439 | 0.908288295 | 1.451388606 | 0.24789244 |
| ZNF205 | 0.842360622 | 0.62772478 | 1.13038618 | 0.252950423 |
| C5 | 1.05276448 | 0.963806271 | 1.14993343 | 0.253644562 |
| RUNX3 | 0.924566215 | 0.807204824 | 1.058991053 | 0.257463185 |
| MX1 | 0.95449876 | 0.879991339 | 1.035314603 | 0.261424857 |
| CYBB | 0.929555334 | 0.818094263 | 1.056202393 | 0.262323369 |
| DNAJC14 | 0.881438538 | 0.706866369 | 1.09912415 | 0.262427763 |
| IL13RA1 | 1.083494854 | 0.941261415 | 1.247221102 | 0.264049325 |
| ATG16L1 | 0.871308705 | 0.683826609 | 1.110192041 | 0.265121056 |
| TREM1 | 1.048059277 | 0.964514859 | 1.138840151 | 0.26807384 |
| TNFRSF13B | 0.922694801 | 0.799996579 | 1.06421167 | 0.269102745 |
| TLR2 | 1.078123292 | 0.943221159 | 1.232319507 | 0.270069092 |
| PMCH | 1.137344337 | 0.903494331 | 1.431721369 | 0.273153544 |
| CLEC7A | 0.895950269 | 0.735649821 | 1.091180698 | 0.274665339 |
| NUP107 | 0.918249839 | 0.787483403 | 1.070730842 | 0.276564569 |
| PRM1 | 0.803551541 | 0.541007356 | 1.193505177 | 0.278553242 |
| IL11 | 1.049632547 | 0.960985118 | 1.146457383 | 0.281935026 |
| TNFSF15 | 1.054346992 | 0.955813697 | 1.163037925 | 0.290427461 |
| CREB1 | 1.097675778 | 0.92307401 | 1.305303908 | 0.291716488 |
| ITGAX | 0.904813239 | 0.750811481 | 1.090402874 | 0.293358195 |
| MME | 1.043320074 | 0.963816891 | 1.129381305 | 0.294338564 |
| KLRG1 | 0.891850102 | 0.719586994 | 1.105351555 | 0.295907915 |
| HLA-DQB1 | 0.963993181 | 0.899286715 | 1.033355479 | 0.300939457 |
| JAK1 | 1.09235302 | 0.922590184 | 1.293353366 | 0.305349769 |
| CFI | 1.041484905 | 0.962937476 | 1.126439499 | 0.309637274 |
| MAGEB2 | 1.044478219 | 0.960202592 | 1.1361506 | 0.310659885 |
| IL17B | 1.146038531 | 0.879395434 | 1.493530969 | 0.313065588 |
| CD53 | 0.930778041 | 0.80831092 | 1.071800146 | 0.318950046 |
| CMKLR1 | 0.911555489 | 0.759615666 | 1.093886614 | 0.319549568 |
| HDAC3 | 0.868464019 | 0.65681473 | 1.148314308 | 0.322382269 |
| CD180 | 1.112997082 | 0.899865411 | 1.376608647 | 0.323588142 |
| MS4A2 | 1.063506869 | 0.940234969 | 1.202940644 | 0.327305728 |
| ARG1 | 0.760842843 | 0.438391481 | 1.320467791 | 0.331198359 |
| CNOT4 | 0.854818154 | 0.622572538 | 1.173701106 | 0.332148745 |
| IL23A | 0.901277693 | 0.730291667 | 1.112297343 | 0.3328433 |
| CD48 | 0.949433724 | 0.85440955 | 1.055026124 | 0.334841748 |
| SEMG1 | 0.963190232 | 0.892490221 | 1.039490855 | 0.334938975 |
| EOMES | 0.958014164 | 0.877024165 | 1.046483295 | 0.341212362 |
| MAPKAPK2 | 0.883024223 | 0.682581353 | 1.14232798 | 0.343639272 |
| PPARG | 0.958256069 | 0.876793835 | 1.047286897 | 0.3468668 |
| EPCAM | 0.965666588 | 0.897716774 | 1.038759647 | 0.348002587 |
| BAGE | 0.808986271 | 0.518491911 | 1.262235286 | 0.350346594 |
| CFD | 1.029600838 | 0.968446865 | 1.094616467 | 0.350447123 |
| CD47 | 0.922756782 | 0.779384296 | 1.092503509 | 0.350780326 |
| SMAD3 | 1.093674408 | 0.90606135 | 1.320135453 | 0.351043191 |
| CCRL2 | 0.931401136 | 0.801917452 | 1.081792239 | 0.352098511 |
| PTPRC | 0.933739765 | 0.808158816 | 1.078834916 | 0.352221269 |
| FOXJ1 | 1.064940963 | 0.932000606 | 1.216843904 | 0.355047456 |
| ATF1 | 0.926874209 | 0.788076187 | 1.090117699 | 0.35889709 |
| SF3A3 | 0.915789473 | 0.75825507 | 1.106053086 | 0.361042934 |
| EBI3 | 0.936801716 | 0.814237102 | 1.077815604 | 0.361495107 |
| LGALS3 | 0.938516227 | 0.818535189 | 1.076084107 | 0.36322148 |
| USP9Y | 1.036772348 | 0.959036257 | 1.120809451 | 0.363806713 |
| DDX58 | 0.934377158 | 0.80668346 | 1.082284088 | 0.365306429 |
| CCL26 | 1.04140693 | 0.953709843 | 1.137168083 | 0.366010977 |
| HRAS | 0.913503225 | 0.750356261 | 1.112122582 | 0.367444847 |
| C4BPA | 1.027366409 | 0.968464312 | 1.089850938 | 0.370121816 |
| MR1 | 0.92896744 | 0.789868722 | 1.092561942 | 0.373303405 |
| CSF3R | 0.95015281 | 0.847352825 | 1.065424386 | 0.381453611 |
| MAP3K7 | 1.107120511 | 0.880572237 | 1.391953748 | 0.383661048 |
| CD44 | 1.053519018 | 0.936595063 | 1.185039688 | 0.385054952 |
| GZMM | 0.917937126 | 0.75548229 | 1.115325375 | 0.388883407 |
| IFNAR2 | 0.912456425 | 0.74 | 1.125103685 | 0.391368527 |
| CCL24 | 0.940920516 | 0.818293295 | 1.081924322 | 0.392690696 |
| STAT6 | 0.921651706 | 0.763991635 | 1.11184708 | 0.394021454 |
| LTF | 0.982705228 | 0.943826316 | 1.023185673 | 0.396956577 |
| SPANXB1 | 1.145776144 | 0.836069117 | 1.570208666 | 0.397341195 |
| MAPK11 | 0.84918398 | 0.580778807 | 1.241631793 | 0.3990031 |
| CD19 | 0.966803247 | 0.893719403 | 1.045863517 | 0.399894309 |
| CTSG | 1.032962623 | 0.95754485 | 1.114320421 | 0.401795892 |
| IL17RA | 0.888608861 | 0.674030655 | 1.171498211 | 0.402314643 |
| CD163 | 1.040676123 | 0.947400068 | 1.143135651 | 0.405309847 |
| TLK2 | 1.119636244 | 0.85701325 | 1.462737383 | 0.407343636 |
| CD1C | 1.061500814 | 0.921728675 | 1.222468182 | 0.407372729 |
| PSMB7 | 0.912688511 | 0.733728898 | 1.135297139 | 0.411969567 |
| ATG5 | 0.931227478 | 0.784754495 | 1.105039373 | 0.414478399 |
| TNFRSF1B | 0.943708852 | 0.820949556 | 1.08482475 | 0.41515281 |
| TNFSF11 | 0.934944489 | 0.795160205 | 1.099301993 | 0.415570503 |
| CD1D | 1.049096844 | 0.934645688 | 1.177563008 | 0.416095355 |
| IFNA8 | 1.176080944 | 0.794649032 | 1.740600354 | 0.417459992 |
| FCF1 | 0.922392589 | 0.75809505 | 1.122297379 | 0.419573849 |
| MAGEA1 | 1.029001785 | 0.959849295 | 1.103136376 | 0.420559282 |
| CD1E | 1.063153727 | 0.915722024 | 1.234322007 | 0.421378477 |
| SERPINB2 | 1.025013893 | 0.965100753 | 1.088646422 | 0.421405347 |
| IGF2R | 1.073832113 | 0.901661717 | 1.278878081 | 0.424321795 |
| IRAK2 | 0.944380296 | 0.820397972 | 1.087099401 | 0.425482932 |
| CSF3 | 1.083173341 | 0.889476895 | 1.319049986 | 0.426722691 |
| ITGAE | 0.931947735 | 0.780571886 | 1.112679815 | 0.435787854 |
| MIF | 1.065476044 | 0.906842987 | 1.251858609 | 0.44065865 |
| GPI | 0.946351339 | 0.82251808 | 1.088828172 | 0.440929095 |
| NOTCH1 | 0.946111534 | 0.821411946 | 1.089741924 | 0.442377842 |
| IL17A | 0.90341561 | 0.69539038 | 1.173671349 | 0.44684469 |
| MARCO | 0.945425115 | 0.817949078 | 1.092768085 | 0.447584617 |
| ISG15 | 0.96819769 | 0.890540166 | 1.052627161 | 0.448672095 |
| NUBP1 | 0.924873197 | 0.755141213 | 1.13275559 | 0.45027015 |
| CCL8 | 1.024656882 | 0.961786978 | 1.091636454 | 0.450876992 |
| NFKBIA | 0.931578923 | 0.772925915 | 1.122797506 | 0.456850203 |
| CREBBP | 1.110940335 | 0.839882626 | 1.46947727 | 0.460986042 |
| SAP130 | 0.917098779 | 0.727270685 | 1.156474731 | 0.464556515 |
| AMBP | 1.07281969 | 0.887116722 | 1.297396451 | 0.468559994 |
| IFIT1 | 1.025802959 | 0.957287885 | 1.099221799 | 0.470099967 |
| S100A7 | 0.980816567 | 0.929936904 | 1.034480011 | 0.476036778 |
| PIN1 | 0.929897436 | 0.761119968 | 1.136101111 | 0.47692745 |
| CSF1 | 0.849458674 | 0.540331937 | 1.335438439 | 0.479673628 |
| CCL11 | 0.977212239 | 0.916406464 | 1.042052624 | 0.481895616 |
| IRF3 | 0.937430384 | 0.781376698 | 1.12465054 | 0.486747136 |
| ITGA4 | 0.957139688 | 0.845439857 | 1.083597343 | 0.489007312 |
| IL21 | 0.846989433 | 0.528492954 | 1.357427935 | 0.490139625 |
| IL17F | 0.907314524 | 0.686552334 | 1.199063211 | 0.494123997 |
| CD84 | 0.93268071 | 0.761916023 | 1.141718089 | 0.499388137 |
| NCAM1 | 1.080474649 | 0.862947439 | 1.352834963 | 0.499786665 |
| STAT3 | 0.936511698 | 0.773981507 | 1.133171985 | 0.500022582 |
| TNFRSF13C | 0.910283214 | 0.691196511 | 1.198813241 | 0.503405593 |
| IL3RA | 1.07541176 | 0.868081728 | 1.332259873 | 0.505837254 |
| FUT5 | 0.860807232 | 0.552441583 | 1.341298544 | 0.507745205 |
| CD24 | 1.024705679 | 0.952090344 | 1.10285934 | 0.515180382 |
| CR2 | 1.027002115 | 0.946635454 | 1.114191678 | 0.5216084 |
| CCR9 | 0.930237388 | 0.741935307 | 1.166330259 | 0.53088456 |
| SPO11 | 0.831791562 | 0.466746337 | 1.482340936 | 0.532140599 |
| FOS | 0.975259868 | 0.9000022 | 1.056810539 | 0.54093384 |
| IL6R | 1.059499091 | 0.879931037 | 1.275711705 | 0.541873647 |
| IKBKB | 1.068817994 | 0.861697483 | 1.325722689 | 0.544801073 |
| ITGAM | 1.042644544 | 0.908438418 | 1.196677313 | 0.552500702 |
| PAX5 | 0.944720525 | 0.782733633 | 1.140230639 | 0.553486325 |
| LTBR | 0.939507518 | 0.764120681 | 1.155150486 | 0.553929404 |
| FLT3LG | 0.943971412 | 0.77945945 | 1.143205112 | 0.555092053 |
| CCL4 | 1.032478928 | 0.928192732 | 1.148482099 | 0.556303813 |
| IL13RA2 | 1.022225664 | 0.949834421 | 1.100134177 | 0.557483657 |
| IL9 | 0.840944669 | 0.468803808 | 1.50849444 | 0.561216239 |
| IL22 | 1.120558046 | 0.761000534 | 1.649999281 | 0.564240741 |
| NFATC3 | 0.909790407 | 0.65863036 | 1.256727043 | 0.566250022 |
| DPP4 | 0.979884011 | 0.913633309 | 1.05093878 | 0.569397137 |
| CCL16 | 0.91219123 | 0.663969761 | 1.25320894 | 0.570617654 |
| IFNGR1 | 0.949918644 | 0.795013816 | 1.135005974 | 0.571610221 |
| CCR7 | 0.978287536 | 0.906539628 | 1.055713919 | 0.572171837 |
| MUC1 | 0.980031335 | 0.913562718 | 1.051336048 | 0.573502713 |
| TPTE | 1.046913969 | 0.891885264 | 1.228889974 | 0.575011987 |
| IL1RL2 | 1.055663433 | 0.871143604 | 1.279267022 | 0.580516753 |
| LCN2 | 0.988709519 | 0.949421256 | 1.029623581 | 0.583107594 |
| C6 | 1.022324944 | 0.944071075 | 1.10706526 | 0.586836091 |
| IL16 | 0.947428851 | 0.779536215 | 1.151481369 | 0.587372769 |
| TLR1 | 0.973278161 | 0.882187429 | 1.073774515 | 0.589035495 |
| TANK | 0.939482261 | 0.744701885 | 1.185208384 | 0.598469807 |
| TMUB2 | 1.064013532 | 0.843386968 | 1.342355099 | 0.60073881 |
| CCR6 | 0.969750878 | 0.862083305 | 1.090865304 | 0.608969292 |
| CXCR5 | 0.954658865 | 0.796208422 | 1.144641936 | 0.616306482 |
| IRAK4 | 1.059319163 | 0.843663012 | 1.330101086 | 0.619764495 |
| CD55 | 0.980505102 | 0.906865457 | 1.060124462 | 0.621141359 |
| SIGIRR | 0.953654359 | 0.789925604 | 1.151319353 | 0.621467395 |
| RORC | 0.96343302 | 0.830713892 | 1.117356039 | 0.62228936 |
| ROPN1 | 0.919236107 | 0.656843096 | 1.286448812 | 0.623365458 |
| IRAK1 | 0.95758707 | 0.804995747 | 1.139102909 | 0.624590085 |
| CR1 | 0.940455086 | 0.733950026 | 1.205062658 | 0.627440958 |
| CD200 | 1.031606456 | 0.907208374 | 1.173062233 | 0.635058674 |
| BID | 0.964120373 | 0.828628825 | 1.121766545 | 0.636295949 |
| NFATC1 | 0.936684937 | 0.71296327 | 1.230608515 | 0.638546426 |
| RELB | 1.041462704 | 0.877652756 | 1.235847044 | 0.641721473 |
| MAGEA4 | 1.01219625 | 0.961346086 | 1.065736119 | 0.644824184 |
| DDX43 | 1.017486609 | 0.943142993 | 1.09769039 | 0.65428756 |
| TNFSF14 | 0.970028218 | 0.848885427 | 1.108459061 | 0.654811648 |
| SPINK5 | 1.015325893 | 0.949474034 | 1.085744982 | 0.656642067 |
| MAPK14 | 1.050123181 | 0.845408414 | 1.304409415 | 0.658448101 |
| SLC11A1 | 0.959682017 | 0.799435133 | 1.152050412 | 0.658857404 |
| CCND3 | 1.043658498 | 0.863025989 | 1.262097637 | 0.659422731 |
| IRF7 | 0.974020069 | 0.86618983 | 1.095273877 | 0.660130835 |
| KLRF1 | 0.959105807 | 0.795061416 | 1.156997345 | 0.662642055 |
| IL7R | 1.018971911 | 0.936308072 | 1.108933893 | 0.663281076 |
| CC2D1B | 0.939135383 | 0.706826471 | 1.247796035 | 0.664939002 |
| CXCL14 | 0.986299754 | 0.926547951 | 1.049904868 | 0.665276358 |
| BAX | 1.029927804 | 0.89851103 | 1.180565676 | 0.672000317 |
| TAB1 | 0.93331678 | 0.675746581 | 1.289063439 | 0.67532458 |
| JAK3 | 0.918853813 | 0.615908942 | 1.370807066 | 0.678404119 |
| TLR4 | 1.02650866 | 0.904468005 | 1.165016367 | 0.685374407 |
| MAP3K5 | 0.968755941 | 0.829614674 | 1.131233695 | 0.688236434 |
| GZMK | 1.013296032 | 0.949856243 | 1.08097289 | 0.688851223 |
| MAPK8 | 0.948510959 | 0.731207742 | 1.230393208 | 0.69048971 |
| LILRB3 | 0.961086373 | 0.788533424 | 1.171398685 | 0.69423605 |
| S100A12 | 1.018286846 | 0.927442352 | 1.118029707 | 0.703880318 |
| CD22 | 0.949365581 | 0.725458509 | 1.242379814 | 0.704977503 |
| PLAUR | 1.018458414 | 0.924373805 | 1.122119142 | 0.711503121 |
| IFNA1 | 0.9067673 | 0.534233162 | 1.539078807 | 0.716923836 |
| ATG12 | 1.031361224 | 0.870777214 | 1.221559265 | 0.720645278 |
| SYK | 1.027295932 | 0.883816377 | 1.194068089 | 0.725693692 |
| ITGA2B | 1.063501207 | 0.748773512 | 1.510516597 | 0.730924325 |
| NFKB2 | 0.964296802 | 0.782899899 | 1.187723134 | 0.732401845 |
| CD37 | 0.98027723 | 0.872838246 | 1.100941041 | 0.73662668 |
| MASP1 | 0.89841968 | 0.465419102 | 1.734260407 | 0.749563515 |
| ZKSCAN5 | 0.954249764 | 0.713923973 | 1.275475605 | 0.751746898 |
| EDC3 | 1.030018783 | 0.852446635 | 1.244580774 | 0.759330694 |
| C1QB | 1.011615398 | 0.939111352 | 1.089717116 | 0.760859727 |
| HCK | 0.983452581 | 0.882599187 | 1.095830354 | 0.762456147 |
| CD3EAP | 0.972535452 | 0.810515865 | 1.166942247 | 0.764546093 |
| IL1RL1 | 0.960725042 | 0.739038105 | 1.248910711 | 0.764676435 |
| PIK3CG | 0.982366188 | 0.872320219 | 1.106294806 | 0.769140161 |
| DDX50 | 0.967112189 | 0.773368844 | 1.209391861 | 0.769385065 |
| PSEN2 | 1.03440819 | 0.822502946 | 1.300907564 | 0.772393467 |
| TCF7 | 1.046193824 | 0.767265456 | 1.426522604 | 0.775307614 |
| ZNF346 | 1.044766108 | 0.773052244 | 1.411982474 | 0.775668814 |
| CMA1 | 0.978384387 | 0.840328029 | 1.139121837 | 0.778269642 |
| TNFRSF8 | 0.944350877 | 0.629238614 | 1.417266135 | 0.782226208 |
| CSF2 | 0.973073394 | 0.798831254 | 1.185321463 | 0.786282778 |
| IFI16 | 1.012897425 | 0.922958808 | 1.111600197 | 0.787071383 |
| SH2B2 | 1.029763898 | 0.831838271 | 1.27478348 | 0.787685706 |
| XCL2 | 0.98286459 | 0.866302607 | 1.115110118 | 0.78842907 |
| MBL2 | 0.932583637 | 0.557464105 | 1.560122402 | 0.790350928 |
| POU2F2 | 0.958242593 | 0.698570892 | 1.314439061 | 0.791390056 |
| LILRB1 | 1.02000319 | 0.878191016 | 1.184715499 | 0.795392654 |
| CD40 | 0.980488187 | 0.844376003 | 1.138541458 | 0.796088473 |
| ITGB4 | 0.981677521 | 0.853181393 | 1.129526222 | 0.796134779 |
| IL12RB2 | 0.966338801 | 0.744373289 | 1.254492461 | 0.797057707 |
| TNFSF8 | 1.027306782 | 0.831389776 | 1.269391632 | 0.802940697 |
| ARG2 | 1.019195557 | 0.877048069 | 1.184381585 | 0.804058713 |
| IL19 | 0.981328816 | 0.842464355 | 1.14308248 | 0.808692063 |
| ZNF143 | 0.962904175 | 0.700892324 | 1.322862896 | 0.81554472 |
| HAVCR2 | 0.984518157 | 0.858012618 | 1.129675697 | 0.824037953 |
| TNFSF18 | 1.033030658 | 0.774091866 | 1.378586169 | 0.825306743 |
| EIF2B4 | 1.027143206 | 0.808501823 | 1.304911301 | 0.826415586 |
| PSMD7 | 1.018218033 | 0.86609081 | 1.197066117 | 0.826905946 |
| IL5 | 0.96047319 | 0.664865828 | 1.38751115 | 0.829856291 |
| GATA3 | 1.017870578 | 0.861019128 | 1.203295583 | 0.835661384 |
| CD207 | 1.028087382 | 0.78822457 | 1.340942297 | 0.838076558 |
| TOLLIP | 1.025024505 | 0.805072275 | 1.305069456 | 0.841041739 |
| TNFAIP3 | 1.011972954 | 0.895898083 | 1.143086786 | 0.848155391 |
| ELANE | 0.972721211 | 0.731968269 | 1.292660617 | 0.848813814 |
| PASD1 | 1.01316623 | 0.879370121 | 1.167319408 | 0.856356738 |
| CD83 | 1.012506285 | 0.883826284 | 1.159921351 | 0.85776696 |
| AGK | 0.981199921 | 0.796278123 | 1.209066603 | 0.858621753 |
| MS4A1 | 0.992980184 | 0.918689778 | 1.073278128 | 0.859072098 |
| BLK | 1.012768058 | 0.878970124 | 1.166932881 | 0.860688741 |
| PVR | 1.013869631 | 0.868357051 | 1.183766086 | 0.861662798 |
| ST6GAL1 | 0.989202291 | 0.872422311 | 1.121614107 | 0.865498982 |
| PRG2 | 1.022743702 | 0.78430078 | 1.33367798 | 0.868119872 |
| IKBKG | 1.020262155 | 0.801371364 | 1.298941929 | 0.870671114 |
| VEGFA | 1.009537804 | 0.895734197 | 1.137800232 | 0.876382882 |
| TBP | 0.982293721 | 0.784273196 | 1.230312293 | 0.876404714 |
| VCAM1 | 1.006505838 | 0.925893824 | 1.094136255 | 0.878989708 |
| CTAGE1 | 0.966687865 | 0.622284331 | 1.501701685 | 0.880171678 |
| ICAM1 | 0.991265759 | 0.881318511 | 1.114929272 | 0.883721872 |
| SELPLG | 1.017979701 | 0.800535759 | 1.294486423 | 0.884435282 |
| CD1A | 0.988958085 | 0.85044913 | 1.150025391 | 0.885320717 |
| PRKCE | 1.019034117 | 0.784386032 | 1.32387688 | 0.88770519 |
| MAP3K1 | 1.009830765 | 0.87524233 | 1.165115236 | 0.893364637 |
| SIGLEC1 | 0.989005348 | 0.833312854 | 1.173786739 | 0.899335223 |
| CD86 | 0.992742657 | 0.883977483 | 1.114890369 | 0.902086004 |
| LILRA1 | 0.968801368 | 0.577620678 | 1.624900436 | 0.9043831 |
| C8A | 0.98826148 | 0.786617497 | 1.241595511 | 0.919221917 |
| CYLD | 1.011435228 | 0.807326946 | 1.267146136 | 0.921239847 |
| TAL1 | 1.013240061 | 0.777930258 | 1.319726815 | 0.922289443 |
| CCL7 | 0.993742236 | 0.866866966 | 1.13918706 | 0.928227758 |
| C9 | 1.019100024 | 0.669595041 | 1.551034273 | 0.929645515 |
| IFIT2 | 0.996606519 | 0.91190699 | 1.089173089 | 0.940205215 |
| CKLF | 0.996085213 | 0.863448681 | 1.149096379 | 0.957094688 |
| NOD2 | 0.996537801 | 0.870814421 | 1.140412428 | 0.959799455 |
| C1QA | 1.00203535 | 0.916666705 | 1.095354328 | 0.964302888 |
| BCL2 | 0.995627301 | 0.820893688 | 1.207554322 | 0.964499476 |
| PIK3CD | 1.003791421 | 0.838162809 | 1.202149757 | 0.967191636 |
| PYCARD | 0.997647213 | 0.890567403 | 1.117602057 | 0.967565289 |
| IL18 | 0.998049314 | 0.906474788 | 1.098874947 | 0.968280109 |
| MAGEC2 | 0.997607782 | 0.880230581 | 1.130637027 | 0.970085281 |
| CTCFL | 1.00189382 | 0.894222039 | 1.12253018 | 0.973980176 |
| CD28 | 1.003751633 | 0.766700845 | 1.314094471 | 0.978266049 |
| IFITM2 | 0.99839756 | 0.881566457 | 1.130711903 | 0.979850089 |
| TBK1 | 0.997234705 | 0.796745381 | 1.248174236 | 0.980708459 |
| S100A8 | 0.999187733 | 0.93547529 | 1.067239441 | 0.980715196 |
| IL13 | 1.002821764 | 0.752782728 | 1.335912013 | 0.984636287 |
| ITGB2 | 0.999160447 | 0.899825526 | 1.109461302 | 0.987457222 |
| CX3CL1 | 1.000597101 | 0.909946832 | 1.100278085 | 0.990170603 |
| IL10RA | 0.999622384 | 0.901053346 | 1.108974196 | 0.994310622 |
| TXK | 1.000819662 | 0.79263128 | 1.263689714 | 0.99450608 |
| FCER1G | 0.999793236 | 0.925163692 | 1.080442872 | 0.995831618 |
