# Supplementary material for: Immunogenomic characterization in gastric cancer identifies microenvironmental and immunotherapeutically relevant gene signatures
Source: Immun Inflamm Dis. 2021 Sep 28;10(1):43–59. doi: 10.1002/iid3.539 (PMC8669697; doi:10.1002/iid3.539)
Supplement: Supplementary file 5 — Supplementary information. [file IID3-10-43-s002.docx]

**Table-S4.** The difference of immune-related genes in expression between normal tissues and tumor tissues.

| **Gene** | **Normal** | **Tumor** | **logFC** | **pValue** |
| --- | --- | --- | --- | --- |
| CREB5 | 3.463943842 | 6.149279963 | 0.82800195 | 4.86E-49 |
| ICAM4 | 2.626459949 | 3.767739743 | 0.520579729 | 7.97E-46 |
| TNFRSF13C | 5.731776906 | 4.98023876 | -0.202767549 | 8.27E-45 |
| PPIA | 9.072567403 | 8.692950033 | -0.061665018 | 7.17E-44 |
| SELE | 2.368265579 | 6.72067884 | 1.504776089 | 1.23E-43 |
| MFGE8 | 4.680243032 | 5.407429843 | 0.208359596 | 1.48E-42 |
| HPRT1 | 6.876790189 | 6.286024073 | -0.129587532 | 5.83E-41 |
| FOXP3 | 4.745426912 | 4.41258559 | -0.10491362 | 9.20E-41 |
| IL4 | 3.130130064 | 2.85845986 | -0.130984574 | 4.91E-39 |
| NEFL | 2.558420944 | 3.869148927 | 0.596762606 | 2.46E-38 |
| S100B | 2.893438849 | 3.938155557 | 0.444734944 | 2.50E-38 |
| TNFRSF4 | 5.609271028 | 5.125850847 | -0.130021793 | 5.88E-38 |
| CTSW | 5.260341884 | 4.75686859 | -0.145144396 | 1.18E-37 |
| MNX1 | 7.642963558 | 6.454725157 | -0.243776485 | 3.75E-37 |
| PBK | 10.77851321 | 7.936091393 | -0.441657641 | 1.31E-36 |
| IRF5 | 5.721186326 | 5.227051787 | -0.130316878 | 2.40E-36 |
| EGR2 | 5.830610693 | 7.671513697 | 0.395864272 | 5.53E-36 |
| CDKN1A | 8.084436499 | 9.231575097 | 0.191429604 | 7.76E-36 |
| THBS1 | 5.078220485 | 6.341864907 | 0.320584111 | 9.25E-36 |
| CEBPB | 10.09514823 | 11.31421589 | 0.16447451 | 9.37E-36 |
| SH2B2 | 5.027469465 | 5.651640527 | 0.168837292 | 1.10E-35 |
| IL6 | 4.914431207 | 8.37107597 | 0.768388621 | 1.94E-35 |
| TAL1 | 4.759273613 | 5.111102337 | 0.10289308 | 2.14E-35 |
| RRAD | 4.05611835 | 5.27003671 | 0.377713263 | 2.34E-35 |
| IRGM | 2.374648122 | 2.647496067 | 0.156914792 | 7.33E-35 |
| PDGFRB | 6.052037083 | 7.495527307 | 0.308609147 | 9.62E-35 |
| TMEFF2 | 3.467626278 | 4.206507573 | 0.27867452 | 1.05E-34 |
| PSEN1 | 5.689576962 | 5.31677724 | -0.097769365 | 3.55E-34 |
| CSF3 | 5.328174628 | 6.049017243 | 0.183059406 | 4.21E-34 |
| NOD1 | 5.394429696 | 6.0349521 | 0.161871875 | 4.70E-34 |
| AICDA | 3.551823901 | 3.18874169 | -0.155572821 | 1.46E-33 |
| CD164 | 11.59563187 | 10.54253503 | -0.137359622 | 1.59E-33 |
| EGR1 | 8.474873189 | 10.07107079 | 0.248953398 | 1.22E-32 |
| EP300 | 6.523629427 | 7.25460441 | 0.153222114 | 1.32E-32 |
| HLA-DRA | 13.0996804 | 11.45700426 | -0.193301752 | 2.01E-32 |
| ICAM1 | 5.744845029 | 6.827105437 | 0.249006057 | 2.08E-32 |
| THBD | 5.834240534 | 7.213846757 | 0.30622391 | 2.27E-32 |
| NFATC1 | 5.3823888 | 5.811201387 | 0.110589843 | 3.65E-32 |
| IL12A | 3.345566105 | 3.931323003 | 0.232764552 | 3.96E-32 |
| PIN1 | 7.369916985 | 8.001766367 | 0.118670137 | 8.96E-32 |
| TNFSF18 | 4.185679471 | 3.810218747 | -0.135588012 | 2.80E-31 |
| CDK1 | 9.259730093 | 7.475522353 | -0.30879575 | 3.11E-31 |
| CD83 | 6.779765282 | 7.896547247 | 0.219986647 | 1.88E-30 |
| CLEC4C | 3.192142446 | 2.956322067 | -0.110721584 | 5.01E-30 |
| IFNA1 | 2.762964953 | 2.509296527 | -0.138934296 | 9.28E-30 |
| PTGS2 | 5.506967593 | 8.40778908 | 0.610468359 | 1.11E-29 |
| CD79A | 6.283302515 | 5.501519777 | -0.191692828 | 2.67E-29 |
| CXCR6 | 7.045041435 | 5.887788197 | -0.258882418 | 2.92E-29 |
| CXCR3 | 5.614251141 | 5.2214444 | -0.104644649 | 4.29E-29 |
| CD34 | 6.321017394 | 6.894642213 | 0.125318902 | 8.14E-29 |
| KLRF1 | 5.017476325 | 4.111208577 | -0.287399337 | 2.51E-28 |
| FOS | 9.653669946 | 11.02218424 | 0.19126074 | 2.54E-28 |
| CASP10 | 5.631519824 | 5.147392703 | -0.129682474 | 2.84E-28 |
| BCL6 | 5.979938238 | 6.890064647 | 0.204386935 | 3.00E-28 |
| SERPING1 | 7.130022014 | 8.82067884 | 0.306983159 | 6.03E-28 |
| MEFV | 5.096501848 | 4.679688333 | -0.123094896 | 7.04E-28 |
| STAT5B | 5.747139543 | 6.105047993 | 0.087158558 | 1.52E-27 |
| CD38 | 8.09503422 | 6.409252427 | -0.336881088 | 4.82E-27 |
| FLT3 | 5.358993064 | 4.881914057 | -0.134515051 | 5.92E-27 |
| ITGA6 | 10.35906766 | 9.170253507 | -0.175860641 | 9.73E-27 |
| IL17RB | 8.685312938 | 7.09814969 | -0.291134832 | 1.03E-26 |
| CD24 | 12.59107705 | 11.0969143 | -0.182243132 | 3.96E-26 |
| PLA2G1B | 3.961519671 | 3.285439183 | -0.269967729 | 5.51E-26 |
| KIR3DL3 | 5.60349308 | 5.229501527 | -0.099653012 | 6.26E-26 |
| TTK | 9.057639404 | 7.30332728 | -0.310581222 | 6.89E-26 |
| CCL2 | 7.446853692 | 9.1752883 | 0.301122477 | 8.79E-26 |
| CD99 | 8.529527698 | 9.443590317 | 0.146869598 | 9.36E-26 |
| ALAS1 | 9.31179864 | 8.49666657 | -0.13216291 | 9.46E-26 |
| CD8B | 3.772372864 | 3.480448523 | -0.116199043 | 9.98E-26 |
| C1QBP | 11.12511703 | 10.29528403 | -0.11183688 | 2.24E-25 |
| SPO11 | 2.594057251 | 2.40471816 | -0.109342504 | 3.41E-25 |
| CDH5 | 5.947925483 | 6.98726125 | 0.23234051 | 3.63E-25 |
| MAPK1 | 7.071605462 | 6.74075971 | -0.069126588 | 8.22E-25 |
| CD46 | 9.398556849 | 8.569371607 | -0.133249832 | 9.17E-25 |
| PSMB8 | 10.81610683 | 9.79870501 | -0.142518305 | 9.17E-25 |
| IGLL1 | 5.883676945 | 5.637717263 | -0.061606907 | 1.74E-24 |
| FEZ1 | 5.02659014 | 5.87540011 | 0.225107043 | 2.79E-24 |
| CASP3 | 9.853916422 | 9.01000628 | -0.129169124 | 2.91E-24 |
| YTHDF2 | 10.27586821 | 9.901282877 | -0.053572925 | 3.03E-24 |
| PECAM1 | 6.951727093 | 7.7150802 | 0.150309707 | 3.32E-24 |
| STAT2 | 6.862492793 | 6.488304967 | -0.080891097 | 4.24E-24 |
| BID | 8.756627283 | 7.962035947 | -0.137237921 | 4.46E-24 |
| C8B | 3.966163922 | 3.56204449 | -0.15503877 | 4.80E-24 |
| PRG2 | 4.086691099 | 3.781833193 | -0.111847467 | 1.14E-23 |
| DDX50 | 5.886351669 | 6.274833473 | 0.092203436 | 1.21E-23 |
| CDH1 | 8.821400879 | 7.861157073 | -0.166266104 | 1.43E-23 |
| VEGFC | 4.923399658 | 6.038459957 | 0.294525798 | 1.45E-23 |
| IL27 | 5.641096148 | 5.3308271 | -0.081616136 | 1.83E-23 |
| TNFRSF13B | 5.561656288 | 5.156397683 | -0.109151054 | 2.12E-23 |
| HLA-C | 12.46693816 | 11.90578313 | -0.066444667 | 3.21E-23 |
| CD4 | 4.719534035 | 4.502513653 | -0.067913777 | 6.01E-23 |
| IFNL2 | 4.169407687 | 3.715518663 | -0.166278831 | 6.64E-23 |
| AKT3 | 4.589600585 | 5.23373004 | 0.189470904 | 1.02E-22 |
| IFI27 | 12.48341563 | 11.30685997 | -0.142814395 | 1.17E-22 |
| ITGA4 | 6.686861106 | 5.525497433 | -0.275224802 | 1.37E-22 |
| MYD88 | 10.62306016 | 10.03713326 | -0.081852144 | 1.91E-22 |
| REL | 6.266948351 | 6.628760723 | 0.080976074 | 2.52E-22 |
| CD5 | 6.020646043 | 5.685670223 | -0.08258788 | 2.83E-22 |
| CCL28 | 7.8087151 | 6.182651627 | -0.33685946 | 4.66E-22 |
| CFD | 7.517197639 | 9.060611697 | 0.269413517 | 4.71E-22 |
| ISG20 | 9.806772237 | 8.72544249 | -0.168550075 | 4.94E-22 |
| TIGIT | 5.874177154 | 5.005188377 | -0.230962408 | 5.19E-22 |
| BATF | 7.706461286 | 6.96516412 | -0.145911197 | 5.83E-22 |
| HLA-DRB4 | 6.080175108 | 4.76633362 | -0.351232937 | 6.24E-22 |
| CD1D | 6.492252458 | 5.164506493 | -0.330088606 | 6.30E-22 |
| C1R | 8.159493335 | 9.39495671 | 0.203406943 | 7.22E-22 |
| CKLF | 9.101967439 | 8.52944738 | -0.093726152 | 2.03E-21 |
| CHUK | 8.651618171 | 8.193423073 | -0.078503684 | 2.97E-21 |
| ATG16L1 | 5.518100141 | 5.24143502 | -0.074209786 | 3.15E-21 |
| JAM3 | 4.622840325 | 5.263566637 | 0.187261181 | 5.84E-21 |
| CD2 | 8.346009264 | 7.208160907 | -0.211455305 | 9.65E-21 |
| F2RL1 | 9.284050492 | 8.1417552 | -0.189414525 | 1.08E-20 |
| KLRB1 | 8.168724383 | 6.801592853 | -0.264238158 | 1.19E-20 |
| IFNB1 | 3.484516544 | 3.728309737 | 0.097563215 | 1.24E-20 |
| PPARG | 9.331990283 | 8.07600883 | -0.208542317 | 1.43E-20 |
| IL1RL2 | 4.747000067 | 4.28789582 | -0.146746216 | 1.49E-20 |
| CFI | 8.772229767 | 7.09910284 | -0.305306887 | 2.18E-20 |
| ANXA1 | 7.186957406 | 8.232987163 | 0.196034841 | 3.03E-20 |
| CD1C | 6.617115641 | 5.80606908 | -0.188640755 | 3.17E-20 |
| CCR9 | 3.593166802 | 3.23779444 | -0.150244514 | 3.71E-20 |
| BCL2 | 4.999939086 | 5.309801047 | 0.086747287 | 4.39E-20 |
| TANK | 7.139901745 | 6.735832837 | -0.084047886 | 4.82E-20 |
| DDX43 | 5.32172714 | 3.801262817 | -0.485415766 | 5.09E-20 |
| ICOSLG | 5.922222213 | 5.723913833 | -0.04913667 | 5.38E-20 |
| TNFRSF1B | 7.610876003 | 8.264219953 | 0.118816136 | 6.42E-20 |
| LY86 | 7.949817314 | 7.024587303 | -0.178508239 | 7.23E-20 |
| ICOS | 5.973217484 | 5.1353176 | -0.218054747 | 7.72E-20 |
| SH2D1A | 5.796962563 | 5.194853493 | -0.158214106 | 8.23E-20 |
| CD58 | 7.067327804 | 6.60888458 | -0.096758027 | 8.62E-20 |
| MUC1 | 10.73006445 | 9.403715703 | -0.190355913 | 1.16E-19 |
| GZMA | 8.903228016 | 7.53351632 | -0.241005095 | 1.24E-19 |
| CD48 | 7.002463707 | 6.254316833 | -0.163010295 | 1.28E-19 |
| ITCH | 6.451847183 | 6.07221458 | -0.087489494 | 2.70E-19 |
| LAMP3 | 8.474556278 | 7.170534007 | -0.241057268 | 3.27E-19 |
| LAMP2 | 9.76044462 | 9.29744388 | -0.070112734 | 3.33E-19 |
| EBI3 | 5.759580542 | 5.188317573 | -0.150696958 | 3.88E-19 |
| AMBP | 5.365261524 | 4.86720339 | -0.140555432 | 4.13E-19 |
| HAMP | 5.058836852 | 4.605857463 | -0.135335948 | 4.25E-19 |
| CXCL2 | 5.158870112 | 5.877743303 | 0.188207231 | 6.31E-19 |
| NCAM1 | 4.262346543 | 4.691154737 | 0.138295195 | 6.43E-19 |
| CD37 | 4.314774752 | 4.06958099 | -0.084404987 | 7.76E-19 |
| HLA-DOB | 5.142689543 | 4.71393255 | -0.125591947 | 8.80E-19 |
| PSMD7 | 9.762277615 | 9.395938557 | -0.0551805 | 9.20E-19 |
| EPCAM | 12.89126158 | 12.19008548 | -0.080685215 | 9.97E-19 |
| TNFRSF12A | 8.037469093 | 8.796232403 | 0.130144437 | 1.10E-18 |
| GZMM | 4.091622914 | 4.502225167 | 0.137965019 | 1.94E-18 |
| HSD11B1 | 5.203960894 | 6.521161933 | 0.325518924 | 3.33E-18 |
| LRP1 | 4.660204074 | 4.854963763 | 0.059067395 | 4.26E-18 |
| TNFAIP3 | 8.442446327 | 9.24721491 | 0.131357816 | 5.07E-18 |
| CTSS | 10.241289 | 9.421755433 | -0.12032952 | 5.64E-18 |
| TBK1 | 9.441020878 | 9.145052573 | -0.045951406 | 6.20E-18 |
| AGK | 5.963245209 | 5.641227143 | -0.080088634 | 6.54E-18 |
| IL4R | 8.018072202 | 8.556313223 | 0.093733888 | 8.06E-18 |
| ZNF346 | 4.95795408 | 5.19808423 | 0.068235102 | 1.10E-17 |
| ANP32B | 11.24536074 | 11.51703876 | 0.034439879 | 1.12E-17 |
| CCL21 | 5.828970129 | 6.858994887 | 0.234756172 | 1.15E-17 |
| CD40LG | 5.434947584 | 5.198045587 | -0.064296835 | 1.21E-17 |
| TLR6 | 4.771064631 | 4.51139445 | -0.080737798 | 1.36E-17 |
| DNAJC14 | 6.849331355 | 6.517296517 | -0.071689522 | 1.63E-17 |
| IDO1 | 9.500415082 | 7.74556858 | -0.2946194 | 1.76E-17 |
| IRF8 | 9.742704454 | 8.992790303 | -0.115553474 | 4.10E-17 |
| IL17B | 4.218073375 | 4.5417275 | 0.106656957 | 4.43E-17 |
| SLAMF7 | 7.067249312 | 5.94848007 | -0.24862772 | 6.48E-17 |
| PNMA1 | 8.466971797 | 9.30562161 | 0.136256441 | 1.08E-16 |
| BST2 | 7.768911795 | 8.851922027 | 0.188278211 | 1.18E-16 |
| CTSH | 11.39427322 | 10.6886325 | -0.092231619 | 1.95E-16 |
| CD81 | 10.56659016 | 11.10513218 | 0.071716671 | 1.96E-16 |
| HLA-B | 12.60156542 | 12.08535293 | -0.060343357 | 2.23E-16 |
| FCGR1A | 3.169109779 | 2.978893673 | -0.089301006 | 2.93E-16 |
| IL12B | 2.542835942 | 2.770092383 | 0.123495706 | 3.29E-16 |
| ERCC3 | 7.307733792 | 7.609084213 | 0.058298749 | 3.34E-16 |
| GZMK | 8.071367989 | 6.78435086 | -0.250602429 | 3.34E-16 |
| BCL2L1 | 6.115735567 | 5.812549283 | -0.073354985 | 3.40E-16 |
| CCL25 | 3.62393502 | 4.619462467 | 0.350167898 | 3.94E-16 |
| LY9 | 4.791783382 | 4.352522737 | -0.138710857 | 4.35E-16 |
| IL22RA2 | 3.629450525 | 3.34905678 | -0.115996314 | 4.65E-16 |
| TLK2 | 5.711250286 | 5.895867467 | 0.045897485 | 5.85E-16 |
| CCL22 | 6.855004971 | 6.27991641 | -0.126412356 | 6.00E-16 |
| IL2RB | 8.259666163 | 7.479706427 | -0.143101826 | 1.07E-15 |
| IL19 | 4.941026186 | 4.368605017 | -0.17763803 | 1.18E-15 |
| PSMB9 | 10.74759398 | 9.91238018 | -0.116710299 | 1.47E-15 |
| IFNL1 | 4.12416986 | 4.332345657 | 0.0710446 | 1.73E-15 |
| CD27 | 7.277387742 | 6.438137903 | -0.176777201 | 1.83E-15 |
| TLR2 | 8.136344178 | 7.300178817 | -0.156448905 | 1.84E-15 |
| IL23R | 3.577497748 | 3.405385413 | -0.07113277 | 2.82E-15 |
| CSF1R | 6.097192096 | 6.688303763 | 0.133495372 | 4.07E-15 |
| CX3CL1 | 5.940021711 | 6.533781107 | 0.137449919 | 4.17E-15 |
| DUSP6 | 9.175918946 | 9.693412097 | 0.079151938 | 4.55E-15 |
| C8A | 3.720426858 | 3.455331727 | -0.106643934 | 4.70E-15 |
| MAGEC2 | 3.879256784 | 3.630935827 | -0.095438845 | 5.51E-15 |
| FYN | 5.736495785 | 6.218603567 | 0.116420933 | 5.91E-15 |
| IL22 | 3.710104847 | 3.511582747 | -0.079338527 | 8.24E-15 |
| HLA-A | 13.03383172 | 12.6876597 | -0.038835292 | 8.31E-15 |
| SIGLEC1 | 5.452962102 | 5.986407763 | 0.134650422 | 1.13E-14 |
| MCAM | 6.252727632 | 6.712793037 | 0.102427488 | 1.20E-14 |
| CD6 | 4.43038959 | 4.288538603 | -0.046947462 | 1.37E-14 |
| TNFRSF1A | 8.542627031 | 8.94573355 | 0.066519992 | 1.57E-14 |
| CD3D | 8.943419059 | 8.166518787 | -0.131105258 | 1.62E-14 |
| ATG5 | 8.716037813 | 8.335838943 | -0.064345052 | 1.69E-14 |
| INPP5D | 4.818987582 | 5.12874051 | 0.089874496 | 1.73E-14 |
| CEACAM1 | 6.989904798 | 6.265066407 | -0.157943005 | 1.85E-14 |
| NFKBIA | 7.005205881 | 7.292993227 | 0.058083603 | 2.01E-14 |
| SYCP1 | 2.835798125 | 2.713884153 | -0.063395695 | 2.24E-14 |
| NFKB1 | 8.412599044 | 8.0698947 | -0.060001736 | 2.26E-14 |
| CTSL | 9.346083528 | 10.06933795 | 0.107534995 | 3.01E-14 |
| IL2RG | 6.458361286 | 5.833612377 | -0.146778621 | 3.91E-14 |
| CXCL12 | 6.739115999 | 7.812507187 | 0.213226253 | 3.91E-14 |
| ATG12 | 7.269264278 | 7.553732073 | 0.055380257 | 5.47E-14 |
| CD80 | 4.312398224 | 3.97529149 | -0.117429758 | 5.64E-14 |
| ELK1 | 5.615778334 | 5.423433707 | -0.050279445 | 5.91E-14 |
| TMUB2 | 6.641931477 | 6.952184187 | 0.065863465 | 7.04E-14 |
| HLA-DQA1 | 6.921802449 | 5.837321457 | -0.245841248 | 7.71E-14 |
| TIRAP | 5.309351278 | 5.12150549 | -0.051967637 | 1.18E-13 |
| IL23A | 4.367539709 | 4.187372303 | -0.060775625 | 1.26E-13 |
| CD180 | 6.757109056 | 6.322654463 | -0.095875762 | 1.40E-13 |
| LIF | 5.936150391 | 6.551189817 | 0.142229308 | 1.78E-13 |
| SDHA | 10.59895829 | 10.23325944 | -0.05065674 | 1.79E-13 |
| MR1 | 5.842750409 | 5.496001533 | -0.088265255 | 2.02E-13 |
| CFB | 7.46699011 | 6.893162053 | -0.115360888 | 2.37E-13 |
| IL1RL1 | 5.350656068 | 5.053514513 | -0.082428725 | 2.85E-13 |
| CD28 | 3.992508463 | 3.722353817 | -0.101080273 | 2.91E-13 |
| IFNA7 | 3.252582833 | 3.09637325 | -0.071006408 | 3.64E-13 |
| ITGA1 | 5.366261032 | 6.373481013 | 0.248164313 | 3.69E-13 |
| TFRC | 7.608245442 | 7.30781184 | -0.0581243 | 4.00E-13 |
| SLAMF1 | 5.216410124 | 4.86293249 | -0.101230738 | 4.15E-13 |
| IRF4 | 4.567286792 | 4.314936827 | -0.081997945 | 4.78E-13 |
| IRF3 | 5.916731505 | 6.210345823 | 0.069873179 | 5.22E-13 |
| CEACAM8 | 3.021740445 | 2.83733849 | -0.090841472 | 5.26E-13 |
| CXCL11 | 8.382067548 | 6.639504813 | -0.336230501 | 6.22E-13 |
| AIRE | 4.569854878 | 4.785220127 | 0.066436941 | 6.45E-13 |
| STAT1 | 9.021789875 | 8.321091323 | -0.116640933 | 6.45E-13 |
| IL1RN | 5.931180177 | 5.400001213 | -0.135359467 | 6.50E-13 |
| CCL17 | 4.85887759 | 4.649817787 | -0.063448905 | 6.65E-13 |
| C7 | 4.923056158 | 5.994080217 | 0.283984194 | 8.39E-13 |
| TFEB | 6.793785227 | 7.164415223 | 0.076633342 | 8.58E-13 |
| BLNK | 9.275484619 | 8.612937867 | -0.106917237 | 1.17E-12 |
| TFE3 | 5.373056383 | 5.52942887 | 0.041387496 | 1.20E-12 |
| CTAGE1 | 3.125176748 | 2.945493283 | -0.085428522 | 1.24E-12 |
| IL18R1 | 6.355963865 | 5.61894172 | -0.177812486 | 1.58E-12 |
| ITGA2 | 8.713530604 | 8.05887336 | -0.112679235 | 1.58E-12 |
| LTA | 5.721866131 | 5.535177887 | -0.047856062 | 2.07E-12 |
| ITGAX | 4.741423431 | 4.50756734 | -0.072971194 | 2.10E-12 |
| ZAP70 | 5.439281607 | 5.178365693 | -0.070919269 | 2.13E-12 |
| FADD | 8.647414327 | 8.318516473 | -0.055942555 | 2.40E-12 |
| AMMECR1L | 8.165122862 | 8.416481563 | 0.043742659 | 2.66E-12 |
| MRC1 | 7.921311241 | 8.825505943 | 0.155939723 | 2.70E-12 |
| TGFB2 | 4.03169268 | 4.348927187 | 0.109273882 | 2.70E-12 |
| NFATC4 | 4.956192823 | 5.16846819 | 0.060504447 | 3.41E-12 |
| CCR2 | 5.373217027 | 4.821962947 | -0.156165545 | 3.74E-12 |
| ABL1 | 7.81714338 | 8.177121347 | 0.06495155 | 4.40E-12 |
| PSMB7 | 7.392450332 | 7.6685792 | 0.052906661 | 4.95E-12 |
| TPTE | 2.930341832 | 2.694386513 | -0.121112146 | 5.58E-12 |
| CXCL9 | 9.837463402 | 8.551551277 | -0.202100211 | 6.42E-12 |
| CD9 | 6.621097683 | 6.912207503 | 0.062076113 | 7.04E-12 |
| RUNX3 | 6.314857963 | 5.87330294 | -0.104578232 | 7.34E-12 |
| MAPKAPK2 | 6.198085386 | 6.002850097 | -0.046174989 | 7.49E-12 |
| SLAMF6 | 5.408402909 | 5.05081077 | -0.09868764 | 7.70E-12 |
| TLR5 | 5.520661678 | 6.09774656 | 0.143434998 | 1.50E-11 |
| COG7 | 7.02638745 | 6.710970437 | -0.06626173 | 1.51E-11 |
| IRAK1 | 7.728629078 | 7.444284583 | -0.05407932 | 1.54E-11 |
| TNFRSF17 | 8.155157392 | 6.71791507 | -0.279699164 | 1.64E-11 |
| NLRP3 | 3.696659468 | 3.997182683 | 0.11276136 | 1.73E-11 |
| KLRC2 | 6.203917285 | 5.164934457 | -0.26442941 | 1.75E-11 |
| A2M | 7.234736171 | 7.551356867 | 0.061795492 | 1.93E-11 |
| CD86 | 7.022694184 | 6.367029087 | -0.141404257 | 2.26E-11 |
| TNFSF8 | 5.119341028 | 4.79703137 | -0.093816242 | 2.28E-11 |
| CFP | 4.757557592 | 5.065138067 | 0.090380474 | 2.44E-11 |
| MAP3K5 | 9.050066958 | 8.68810223 | -0.058887387 | 2.77E-11 |
| CARD11 | 5.918533832 | 5.6908788 | -0.056588374 | 2.89E-11 |
| MAGEA4 | 3.506791311 | 3.505222647 | -0.000645493 | 2.99E-11 |
| CNOT10 | 7.404422509 | 7.116626883 | -0.057193621 | 3.94E-11 |
| BTLA | 5.073338851 | 4.601692337 | -0.140770992 | 4.13E-11 |
| PTGDR2 | 5.211446445 | 5.059788853 | -0.042606668 | 4.36E-11 |
| IRF2 | 8.501467228 | 8.193266173 | -0.053273167 | 4.73E-11 |
| RELB | 6.63985668 | 6.329502633 | -0.069059963 | 4.76E-11 |
| ALCAM | 7.340249815 | 6.83829371 | -0.102192775 | 4.79E-11 |
| CD1B | 4.071003742 | 3.818470677 | -0.092389603 | 5.68E-11 |
| CD70 | 5.60807979 | 5.916687377 | 0.077282793 | 5.74E-11 |
| HLA-DMB | 10.18160868 | 9.463001977 | -0.105595691 | 5.94E-11 |
| CXCL5 | 8.812679592 | 7.278079483 | -0.276022946 | 6.88E-11 |
| CASP8 | 6.084497251 | 5.87244334 | -0.051177173 | 7.01E-11 |
| SLC11A1 | 5.33047044 | 5.164700063 | -0.045578295 | 7.25E-11 |
| FCER2 | 4.561517217 | 4.418478103 | -0.045964228 | 7.45E-11 |
| CCR3 | 4.610057122 | 4.10600475 | -0.16704933 | 7.85E-11 |
| C3 | 9.448667162 | 10.52736505 | 0.155961641 | 8.23E-11 |
| IFIH1 | 5.6694372 | 5.30927629 | -0.094690307 | 8.45E-11 |
| PRPF38A | 7.3480385 | 7.62085542 | 0.05259376 | 8.50E-11 |
| CCR6 | 5.993857202 | 5.201769357 | -0.204482281 | 1.13E-10 |
| CNOT4 | 4.636950604 | 4.753593833 | 0.035842281 | 2.12E-10 |
| IL2 | 3.232016321 | 3.126191677 | -0.048028246 | 2.15E-10 |
| IL12RB2 | 3.006996201 | 3.221547997 | 0.099431044 | 2.29E-10 |
| CCL8 | 6.85468681 | 7.90195088 | 0.205118127 | 2.71E-10 |
| MAP3K7 | 7.191415993 | 6.974655703 | -0.044153864 | 2.78E-10 |
| BAX | 7.968058651 | 7.530571863 | -0.081468841 | 2.80E-10 |
| LYN | 9.421234917 | 8.921001877 | -0.078710436 | 3.20E-10 |
| CD36 | 5.269971851 | 5.779299773 | 0.133099449 | 3.37E-10 |
| LTBR | 5.773172709 | 5.65435504 | -0.030001912 | 3.96E-10 |
| HLA-DPA1 | 10.46198144 | 9.638466773 | -0.11828054 | 4.01E-10 |
| IL1R2 | 9.144612681 | 8.019108793 | -0.189480155 | 4.11E-10 |
| PRKCD | 6.87443803 | 6.435552633 | -0.09517774 | 4.11E-10 |
| TCF7 | 6.637635588 | 6.411829247 | -0.049933421 | 4.63E-10 |
| GPATCH3 | 6.180120034 | 6.377949757 | 0.045457873 | 5.01E-10 |
| ST6GAL1 | 5.271909676 | 4.974455543 | -0.083787022 | 5.17E-10 |
| IRAK4 | 6.878564698 | 6.609402957 | -0.057587604 | 7.61E-10 |
| IRF1 | 7.960185357 | 8.503927073 | 0.0953272 | 8.01E-10 |
| RORA | 4.694447735 | 5.038711093 | 0.102099292 | 8.42E-10 |
| IL25 | 5.774567639 | 5.61366257 | -0.040770583 | 9.05E-10 |
| NFATC2 | 5.61867203 | 5.85070412 | 0.05838107 | 9.72E-10 |
| CMA1 | 4.060497998 | 4.492237387 | 0.14577749 | 1.04E-09 |
| IL9 | 2.774191607 | 2.658678317 | -0.061358204 | 1.12E-09 |
| CCL4 | 8.029114108 | 8.902790207 | 0.149016742 | 1.14E-09 |
| C9 | 2.832630594 | 2.697189097 | -0.0706858 | 1.25E-09 |
| EWSR1 | 6.348718791 | 6.50201353 | 0.034421081 | 1.39E-09 |
| SPANXB1 | 3.020309912 | 3.141996723 | 0.056985085 | 1.42E-09 |
| LILRB2 | 5.497138276 | 6.003629893 | 0.127154272 | 1.50E-09 |
| MAP3K1 | 7.49094577 | 7.127292717 | -0.071793701 | 1.50E-09 |
| IL1B | 9.965005966 | 8.867279613 | -0.1683791 | 2.14E-09 |
| FCER1G | 8.387953214 | 7.65893258 | -0.131175476 | 2.15E-09 |
| OAS3 | 6.671515314 | 6.296340977 | -0.083500809 | 2.36E-09 |
| MPPED1 | 4.71864031 | 4.580847057 | -0.042756807 | 2.37E-09 |
| ZNF143 | 6.058506622 | 5.94897931 | -0.026320063 | 2.63E-09 |
| SBNO2 | 5.381581793 | 5.231150403 | -0.040902032 | 2.81E-09 |
| MST1R | 7.549455895 | 7.04869017 | -0.099017478 | 3.01E-09 |
| IFI16 | 8.773581159 | 9.364772127 | 0.094078054 | 3.20E-09 |
| TXK | 3.435627074 | 3.216212707 | -0.095210622 | 3.22E-09 |
| LAIR2 | 6.157960908 | 5.532857937 | -0.154427828 | 3.26E-09 |
| IL21R | 4.868466421 | 4.542586413 | -0.099953432 | 3.30E-09 |
| IL7 | 7.128182976 | 6.395370357 | -0.156506462 | 3.57E-09 |
| CREBBP | 6.085447206 | 6.203605377 | 0.027743629 | 3.63E-09 |
| LCP1 | 8.87771109 | 8.282581897 | -0.100107196 | 4.88E-09 |
| RORC | 6.0816039 | 5.685667547 | -0.097122111 | 4.91E-09 |
| REPS1 | 5.263807739 | 5.117096597 | -0.040781328 | 4.94E-09 |
| CHIT1 | 4.457437234 | 4.22873007 | -0.075990011 | 5.60E-09 |
| EDC3 | 6.218017103 | 6.02175384 | -0.046270851 | 6.94E-09 |
| S100A8 | 7.243592262 | 8.09782421 | 0.160828985 | 6.99E-09 |
| SMAD3 | 5.993097777 | 5.835536647 | -0.038436577 | 7.11E-09 |
| MAP2K1 | 9.11321146 | 8.9071778 | -0.032991151 | 7.20E-09 |
| CXCR5 | 5.061667457 | 4.912507947 | -0.043152987 | 7.50E-09 |
| TLR1 | 6.57913072 | 5.877507123 | -0.162692595 | 8.15E-09 |
| TLR10 | 4.120168004 | 3.88593536 | -0.084441263 | 8.25E-09 |
| CD14 | 8.223571711 | 8.823990137 | 0.101666048 | 9.39E-09 |
| COLEC12 | 6.05362721 | 6.93747111 | 0.196610023 | 9.96E-09 |
| SF3A3 | 8.039436466 | 8.274628353 | 0.041600139 | 1.17E-08 |
| CLEC4A | 7.06280433 | 6.392536703 | -0.143852589 | 1.31E-08 |
| IL3RA | 6.118445281 | 6.43044639 | 0.071753785 | 1.36E-08 |
| CD200 | 5.665600727 | 6.149791557 | 0.118308578 | 1.38E-08 |
| PRKCE | 4.883249324 | 4.995006033 | 0.032644981 | 1.49E-08 |
| IL6ST | 5.893042335 | 6.14191784 | 0.059676583 | 1.92E-08 |
| CCL19 | 7.848864191 | 6.804569 | -0.205980112 | 2.09E-08 |
| LCK | 7.111036887 | 6.673131217 | -0.091696068 | 2.17E-08 |
| TREM2 | 7.242779362 | 6.813450113 | -0.088157909 | 2.29E-08 |
| IL21 | 4.002355985 | 3.867422937 | -0.049476947 | 2.45E-08 |
| JAK2 | 6.188463632 | 5.759937423 | -0.103528148 | 4.19E-08 |
| ITK | 6.855224692 | 6.223802797 | -0.139407605 | 4.21E-08 |
| MBL2 | 2.726890737 | 2.62841524 | -0.053063682 | 4.46E-08 |
| MARCO | 5.539886047 | 5.27372555 | -0.071033807 | 4.66E-08 |
| SSX1 | 3.312814281 | 3.176911973 | -0.060432211 | 5.02E-08 |
| AXL | 6.54357677 | 6.868586803 | 0.069933859 | 5.07E-08 |
| KLRG1 | 5.24236265 | 5.047394543 | -0.054678295 | 5.13E-08 |
| PSMB10 | 9.832666128 | 9.427518903 | -0.060704518 | 5.19E-08 |
| IL12RB1 | 5.21440781 | 5.024481943 | -0.053528565 | 5.55E-08 |
| CTSG | 6.049348201 | 6.482286593 | 0.099723101 | 5.61E-08 |
| CD22 | 4.357713978 | 4.266703537 | -0.030449636 | 7.58E-08 |
| PVR | 5.668857345 | 5.537311827 | -0.033872197 | 9.99E-08 |
| CD63 | 12.4894047 | 12.63637012 | 0.016877386 | 1.06E-07 |
| CCL7 | 4.556302222 | 4.277110223 | -0.091227058 | 1.20E-07 |
| MAGEC1 | 3.853228974 | 3.659470907 | -0.074432843 | 1.23E-07 |
| MAGEA1 | 4.23211777 | 4.106614963 | -0.043430088 | 1.37E-07 |
| MX1 | 9.552923962 | 8.91928702 | -0.099013991 | 1.38E-07 |
| CCND3 | 6.363773926 | 6.209285653 | -0.035455281 | 1.52E-07 |
| DUSP4 | 8.929335971 | 8.30343904 | -0.104843911 | 1.61E-07 |
| CD1E | 4.659493391 | 4.351549107 | -0.098644028 | 1.62E-07 |
| KLRD1 | 5.534658158 | 5.1338517 | -0.108452591 | 2.08E-07 |
| HLA-G | 10.76740259 | 10.47395163 | -0.039864424 | 2.30E-07 |
| BIRC5 | 6.689842154 | 6.291787577 | -0.088502208 | 2.84E-07 |
| CMKLR1 | 4.936810596 | 5.168254423 | 0.066097797 | 2.91E-07 |
| TRAF6 | 6.872810806 | 6.733525117 | -0.029538267 | 2.91E-07 |
| MAPK11 | 5.059041385 | 5.185133023 | 0.035516961 | 2.93E-07 |
| LILRB1 | 6.235568941 | 5.99213531 | -0.057450997 | 3.14E-07 |
| NUP107 | 9.600897355 | 9.366886167 | -0.035599722 | 3.15E-07 |
| CCL20 | 10.714738 | 9.76711647 | -0.133591967 | 3.73E-07 |
| FN1 | 7.714392569 | 8.221811423 | 0.091903719 | 3.73E-07 |
| MAPK8 | 6.513651199 | 6.32677918 | -0.041995223 | 3.89E-07 |
| CLEC7A | 4.890657016 | 4.552587303 | -0.103341608 | 3.93E-07 |
| IL10 | 3.546123436 | 3.75148505 | 0.081219054 | 4.06E-07 |
| IL7R | 8.690526023 | 8.10367837 | -0.100866588 | 4.08E-07 |
| MAGEA12 | 4.556601887 | 4.656188807 | 0.031191233 | 4.14E-07 |
| C8G | 3.913375032 | 4.12340493 | 0.07542277 | 5.05E-07 |
| TARP | 5.731924791 | 5.303405347 | -0.11210066 | 5.55E-07 |
| HLA-DQB1 | 6.201333446 | 6.872450303 | 0.148246104 | 5.73E-07 |
| TLR7 | 5.637581883 | 5.309162253 | -0.086592251 | 5.88E-07 |
| CLU | 6.146553292 | 6.787613913 | 0.143126866 | 6.09E-07 |
| CCRL2 | 7.086094887 | 6.636745087 | -0.094514922 | 6.42E-07 |
| C5 | 6.278150092 | 5.709944843 | -0.13686271 | 7.00E-07 |
| MME | 5.533632142 | 6.17207992 | 0.157530001 | 7.18E-07 |
| TAP2 | 6.34257204 | 6.09362833 | -0.057766493 | 7.18E-07 |
| CAMP | 5.950554328 | 6.082826853 | 0.031717869 | 8.64E-07 |
| CD8A | 7.5743692 | 6.963429283 | -0.121327779 | 8.77E-07 |
| ADA | 7.867241975 | 7.41450272 | -0.085508021 | 9.04E-07 |
| CRP | 3.205210474 | 3.342060543 | 0.060318773 | 9.23E-07 |
| CCR5 | 8.040124741 | 7.611504863 | -0.079036169 | 1.24E-06 |
| CR2 | 4.59029508 | 4.18527201 | -0.133265508 | 1.51E-06 |
| S100A7 | 4.663121583 | 4.750462603 | 0.026771965 | 1.56E-06 |
| TLR8 | 5.87731013 | 5.40248886 | -0.121531835 | 1.67E-06 |
| IL15RA | 7.302955615 | 7.548184057 | 0.04764914 | 1.82E-06 |
| APP | 9.192308734 | 9.368815703 | 0.027439437 | 2.10E-06 |
| IL22RA1 | 6.909071278 | 6.384503007 | -0.113917476 | 2.49E-06 |
| MICA | 7.063870777 | 6.76880337 | -0.061558143 | 2.72E-06 |
| CCL27 | 3.693476612 | 3.56851314 | -0.049656357 | 2.82E-06 |
| MS4A1 | 6.045819567 | 5.260674603 | -0.200690108 | 3.08E-06 |
| IL13RA1 | 8.050162379 | 8.39494396 | 0.060502811 | 3.12E-06 |
| IKBKE | 6.165489327 | 5.992180247 | -0.041134378 | 3.23E-06 |
| ELANE | 3.805040545 | 3.708806023 | -0.036957012 | 3.44E-06 |
| CD74 | 7.120180908 | 6.878644697 | -0.049789559 | 3.54E-06 |
| MAP2K4 | 7.198197061 | 6.99925898 | -0.040433408 | 3.54E-06 |
| CD44 | 6.594629372 | 6.927450303 | 0.071032876 | 3.61E-06 |
| IFNGR1 | 8.62344011 | 8.404368723 | -0.037124053 | 4.79E-06 |
| PIK3CD | 5.632949061 | 5.811579063 | 0.045039786 | 7.26E-06 |
| SPINK5 | 7.209119302 | 6.481953923 | -0.153394259 | 7.47E-06 |
| FPR2 | 5.693436276 | 5.308074177 | -0.101111123 | 8.51E-06 |
| POLR2A | 5.078895417 | 4.994012903 | -0.024315218 | 9.00E-06 |
| TNFSF13 | 8.657434966 | 8.316408103 | -0.057979089 | 9.25E-06 |
| LY96 | 8.705349763 | 8.166851427 | -0.092122281 | 9.74E-06 |
| CASP1 | 9.132317962 | 8.563000807 | -0.09286463 | 1.01E-05 |
| SYT17 | 5.293714388 | 5.657547037 | 0.095896315 | 1.03E-05 |
| SMPD3 | 5.391684742 | 5.072160587 | -0.08813572 | 1.16E-05 |
| FUT7 | 4.497025352 | 4.58800787 | 0.028896849 | 1.24E-05 |
| LCN2 | 11.75969386 | 11.07921251 | -0.085995162 | 1.42E-05 |
| SEMG1 | 3.946517855 | 3.827950487 | -0.044008107 | 1.55E-05 |
| IL2RA | 4.831917104 | 4.605295523 | -0.069301967 | 1.82E-05 |
| LRRN3 | 4.25853911 | 4.56596779 | 0.100562083 | 2.05E-05 |
| SYK | 6.508276757 | 6.31282509 | -0.043989822 | 2.23E-05 |
| NFATC3 | 6.185271078 | 6.295361993 | 0.025452513 | 2.53E-05 |
| ITGB4 | 5.147075788 | 5.021917417 | -0.035514721 | 2.54E-05 |
| NCR1 | 4.411118233 | 4.320878987 | -0.029819604 | 2.73E-05 |
| CSF3R | 5.987238066 | 6.247698967 | 0.061434305 | 2.79E-05 |
| IL13 | 5.175503816 | 5.276442607 | 0.027866278 | 3.44E-05 |
| PRM1 | 5.233484475 | 5.136272463 | -0.027050083 | 3.50E-05 |
| BCL10 | 6.517477465 | 6.74696071 | 0.049924071 | 4.34E-05 |
| VCAM1 | 8.290592328 | 8.77152383 | 0.081352316 | 4.46E-05 |
| IGF1R | 5.454974178 | 5.674266873 | 0.056861638 | 4.61E-05 |
| CXCL16 | 8.762016502 | 8.429210877 | -0.055865356 | 4.65E-05 |
| IL1RAPL2 | 3.477433925 | 3.573387207 | 0.039269148 | 5.50E-05 |
| ICAM2 | 7.615099793 | 7.91715371 | 0.056118919 | 5.50E-05 |
| RELA | 7.204946247 | 7.086251337 | -0.023965031 | 5.57E-05 |
| NFKB2 | 4.144162741 | 4.21469591 | 0.024347886 | 5.69E-05 |
| LTB | 6.208112658 | 6.03070623 | -0.041827778 | 7.42E-05 |
| THY1 | 7.241973826 | 7.588451117 | 0.067422484 | 7.76E-05 |
| HLA-DMA | 9.978460712 | 9.528046697 | -0.066636797 | 9.79E-05 |
| MAPK3 | 8.204730653 | 8.05805183 | -0.026024888 | 1.00E-04 |
| CD3G | 6.544050824 | 6.213257957 | -0.074834001 | 0.000104605 |
| ENTPD1 | 6.588469476 | 6.918941147 | 0.070607908 | 0.000113536 |
| HLA-E | 10.94348274 | 11.12242052 | 0.023398844 | 0.000115407 |
| ITGB2 | 6.940385497 | 6.66154372 | -0.059159258 | 0.000125203 |
| TNFSF4 | 5.724700805 | 5.319893637 | -0.105802893 | 0.000129337 |
| CD160 | 4.738804198 | 4.497017133 | -0.075554671 | 0.000130919 |
| CYBB | 5.683490171 | 5.482493217 | -0.051945024 | 0.000133599 |
| HLA-DPB1 | 8.654054888 | 8.35758751 | -0.050289715 | 0.000136882 |
| ECSIT | 7.156214957 | 7.306186433 | 0.029921844 | 0.000145425 |
| CCL11 | 9.319034372 | 8.670786143 | -0.10401767 | 0.000146012 |
| CD96 | 4.644349191 | 4.36905262 | -0.088155965 | 0.000153233 |
| TPSAB1 | 5.847587388 | 5.70137217 | -0.036532337 | 0.000168694 |
| USP9Y | 4.014460259 | 5.018353487 | 0.322008064 | 0.000184146 |
| TNFRSF11A | 7.716342044 | 7.145713337 | -0.110839057 | 0.000185615 |
| ABCF1 | 8.477586658 | 8.675129883 | 0.033231731 | 0.000190843 |
| PDCD1LG2 | 4.0918495 | 3.9792298 | -0.040263866 | 0.000202515 |
| ENG | 6.333173633 | 6.208854547 | -0.028601499 | 0.000226119 |
| IL26 | 3.44492945 | 3.235688953 | -0.090401509 | 0.000230596 |
| MIF | 12.10585024 | 11.93006121 | -0.021102964 | 0.000241684 |
| TNFSF11 | 4.238434817 | 4.004587203 | -0.081878064 | 0.000244534 |
| EIF2B4 | 8.420627934 | 8.520919787 | 0.017081349 | 0.000250327 |
| IL6R | 5.437971508 | 5.650993013 | 0.055435813 | 0.000258251 |
| NRP1 | 5.092094284 | 5.237920843 | 0.040735125 | 0.000274279 |
| CD59 | 9.923864631 | 9.72634725 | -0.029003956 | 0.000292362 |
| TNFRSF14 | 7.240327876 | 7.417419103 | 0.034862256 | 0.00030739 |
| CD3E | 5.319984303 | 5.218534197 | -0.027777355 | 0.0003219 |
| TNFSF10 | 9.253508195 | 9.560363497 | 0.047065048 | 0.000333202 |
| STAT4 | 6.275839093 | 6.01806214 | -0.06050936 | 0.000335763 |
| ROPN1 | 3.493510695 | 3.565918183 | 0.029596045 | 0.000373611 |
| HRAS | 6.978148622 | 7.17672559 | 0.040481434 | 0.000380776 |
| CD276 | 5.269339809 | 5.349077027 | 0.02166776 | 0.000426532 |
| LAG3 | 5.189374065 | 4.968233617 | -0.06282752 | 0.00043464 |
| CCL18 | 8.993636067 | 8.248327697 | -0.124802855 | 0.000458114 |
| F13A1 | 6.77486284 | 7.336526747 | 0.114905486 | 0.000459836 |
| DOCK9 | 4.979901424 | 5.072761583 | 0.026654171 | 0.000480966 |
| IL17RA | 6.571909741 | 6.698617207 | 0.027550645 | 0.000482767 |
| CD33 | 5.564892881 | 5.391549613 | -0.045653932 | 0.000484575 |
| C1QB | 9.062388723 | 8.70818549 | -0.057519237 | 0.000510551 |
| BLK | 4.399532413 | 4.31534405 | -0.027874615 | 0.000537821 |
| S100A12 | 5.404220428 | 5.873829417 | 0.120214848 | 0.000551959 |
| IL5RA | 3.628484766 | 3.574438173 | -0.021650715 | 0.000592098 |
| LGALS3 | 8.455424585 | 8.564970667 | 0.018571105 | 0.000612045 |
| MASP2 | 4.637363532 | 4.586246707 | -0.015990863 | 0.000641963 |
| CD84 | 5.107639867 | 4.97549806 | -0.037815856 | 0.00064432 |
| IL16 | 5.140289716 | 4.980749403 | -0.045486848 | 0.000670782 |
| PDGFC | 7.399280767 | 7.708133493 | 0.058996514 | 0.0006757 |
| IFNG | 4.850713604 | 4.654437963 | -0.059590034 | 0.000698251 |
| XCL2 | 6.041615138 | 5.679988587 | -0.089046254 | 0.000718882 |
| LILRA1 | 3.848022142 | 3.9288107 | 0.029975556 | 0.000827813 |
| SAP130 | 7.946847447 | 7.839531933 | -0.01961513 | 0.000915256 |
| C1S | 8.369777196 | 7.989083323 | -0.067159243 | 0.001007614 |
| ITGAM | 5.290937627 | 5.44636293 | 0.041769712 | 0.001124257 |
| NT5E | 6.373409468 | 6.703140233 | 0.072771765 | 0.0011442 |
| CD209 | 5.135849222 | 5.236291133 | 0.027942464 | 0.001235893 |
| ILF3 | 7.989152246 | 8.113740987 | 0.022324826 | 0.001235893 |
| CD207 | 3.522477645 | 3.43160955 | -0.037705139 | 0.001320494 |
| CXCR4 | 9.509703241 | 9.050503513 | -0.071402264 | 0.001410412 |
| SPP1 | 5.786710821 | 6.300877287 | 0.122809162 | 0.00148535 |
| ISG15 | 10.07559925 | 9.724666203 | -0.051145011 | 0.001574706 |
| IFNAR2 | 7.74670624 | 7.63334361 | -0.021267898 | 0.001686155 |
| CD79B | 4.877145942 | 4.991835727 | 0.033533314 | 0.00174456 |
| CCR7 | 5.89515785 | 5.574889243 | -0.080587301 | 0.001756462 |
| CTLA4 | 5.062371385 | 4.905076727 | -0.045537646 | 0.001879654 |
| IL34 | 6.493732095 | 6.411883407 | -0.018299674 | 0.001937637 |
| IFITM2 | 11.00947938 | 11.2276994 | 0.028316097 | 0.002157363 |
| FCGR2B | 6.883179718 | 7.268469707 | 0.078576474 | 0.002171827 |
| MAGEB2 | 3.51288194 | 3.594857517 | 0.033279499 | 0.002521684 |
| LILRB3 | 6.925587015 | 6.81693181 | -0.022813808 | 0.002903442 |
| TNFRSF10C | 4.696494706 | 4.798226547 | 0.030916893 | 0.003028834 |
| PPBP | 5.099516661 | 4.744762587 | -0.104024612 | 0.003048558 |
| MEF2C | 6.438297784 | 6.680303377 | 0.053234316 | 0.003108433 |
| FCER1A | 4.468426455 | 4.353268113 | -0.037668002 | 0.003220998 |
| GATA3 | 4.459898672 | 4.64169927 | 0.057642122 | 0.003220998 |
| HCK | 6.371423638 | 6.659676833 | 0.063836405 | 0.003348021 |
| MRPS5 | 6.423407856 | 6.506086603 | 0.018451123 | 0.00336963 |
| CXCL1 | 9.958225045 | 9.4301723 | -0.078604488 | 0.00346845 |
| POU2AF1 | 5.931237175 | 5.77298105 | -0.039016573 | 0.003650664 |
| CSF2 | 5.538188574 | 5.470734133 | -0.017679733 | 0.003685783 |
| DEFB1 | 6.117034902 | 6.70311568 | 0.131999324 | 0.003744998 |
| SIGIRR | 7.589715364 | 7.716768397 | 0.023951025 | 0.003768925 |
| TYK2 | 7.784873999 | 7.68705745 | -0.018242238 | 0.003792991 |
| MAPK14 | 6.589376546 | 6.482364297 | -0.023621871 | 0.003890657 |
| GZMB | 7.718799348 | 7.343281987 | -0.071951454 | 0.004041443 |
| ATM | 5.151290012 | 5.231956707 | 0.022416838 | 0.004277585 |
| ATG7 | 5.954485402 | 5.880193267 | -0.018113259 | 0.004414119 |
| MTMR14 | 6.31992506 | 6.39290273 | 0.016563692 | 0.004568856 |
| IFIT1 | 8.666318426 | 9.0380942 | 0.060599347 | 0.004938956 |
| CXCR2 | 5.903318192 | 6.295808737 | 0.092865607 | 0.004969704 |
| DMBT1 | 8.670734979 | 9.413768967 | 0.118618157 | 0.005126059 |
| ITGB3 | 4.1130483 | 4.074564977 | -0.013561976 | 0.005352427 |
| CXCR1 | 5.756120536 | 5.664998877 | -0.023021133 | 0.005468963 |
| ZNF205 | 6.765619836 | 6.821906503 | 0.011952869 | 0.005674075 |
| DHX16 | 8.146648045 | 8.243026683 | 0.016967583 | 0.005674077 |
| IL1RAP | 5.757721924 | 5.621399773 | -0.034568696 | 0.005726428 |
| PLAU | 7.330201966 | 7.064085883 | -0.053350066 | 0.005743975 |
| IL5 | 2.717932124 | 2.8041208 | 0.045039074 | 0.005779205 |
| GTF3C1 | 7.362763886 | 7.44292888 | 0.015623014 | 0.005832439 |
| CD53 | 5.857919481 | 6.004980167 | 0.035771118 | 0.006807455 |
| TP53 | 4.393884618 | 4.5141221 | 0.038948456 | 0.007004475 |
| TLR3 | 6.341324673 | 6.034836477 | -0.071469565 | 0.007014987 |
| CCL1 | 4.112047166 | 4.04249589 | -0.024610506 | 0.007358873 |
| ITGA2B | 3.931380457 | 3.892938173 | -0.014176554 | 0.00778683 |
| CXCL13 | 8.18472392 | 7.67644328 | -0.092495731 | 0.00959023 |
| PASD1 | 4.23345298 | 4.119851383 | -0.03924257 | 0.009986922 |
| TREM1 | 6.282552304 | 5.92216214 | -0.085226787 | 0.010132106 |
| PRF1 | 6.783730508 | 6.6414981 | -0.030570156 | 0.010458135 |
| ZKSCAN5 | 5.867004494 | 5.959969583 | 0.022680871 | 0.010855451 |
| ITGAL | 6.107482975 | 5.96806635 | -0.033314362 | 0.010886562 |
| MAVS | 6.572255015 | 6.474413057 | -0.02163905 | 0.010949023 |
| CREB1 | 7.259163891 | 7.3435008 | 0.016664601 | 0.0110749 |
| C4BPA | 6.088377675 | 5.992885787 | -0.022806976 | 0.011924897 |
| CXCL14 | 7.405746116 | 7.651856123 | 0.047164655 | 0.012546759 |
| TNFRSF18 | 4.765291067 | 4.705658393 | -0.018167745 | 0.01283224 |
| CXCL10 | 9.434607347 | 8.99656766 | -0.068587782 | 0.013086736 |
| SPN | 5.598967667 | 5.639879107 | 0.010503389 | 0.013345685 |
| ARG1 | 3.166649498 | 3.136346723 | -0.013872133 | 0.014071513 |
| IL13RA2 | 5.047371461 | 5.32639895 | 0.077628226 | 0.014347578 |
| IFNA17 | 4.358587733 | 4.30224072 | -0.018772501 | 0.015288577 |
| CD3EAP | 6.933779457 | 6.827649247 | -0.022253004 | 0.015415237 |
| OSM | 5.595783334 | 5.876577713 | 0.070636128 | 0.015887595 |
| TICAM1 | 6.719784031 | 6.593829713 | -0.027298237 | 0.015887595 |
| CYFIP2 | 6.80436091 | 6.639871833 | -0.035304271 | 0.016062492 |
| SERPINB2 | 4.714381275 | 4.566267053 | -0.046053204 | 0.016870636 |
| TNFRSF9 | 4.438430665 | 4.375578607 | -0.020575855 | 0.017618726 |
| MAP4K2 | 5.075805977 | 5.17992905 | 0.029295414 | 0.017955051 |
| CSF2RB | 8.474425273 | 8.188094077 | -0.049587851 | 0.018003559 |
| TAB1 | 4.759372874 | 4.802410513 | 0.012987244 | 0.018395735 |
| LTK | 4.478095757 | 4.59077939 | 0.035853727 | 0.018947177 |
| NUBP1 | 7.869161411 | 7.759771847 | -0.020195666 | 0.020147086 |
| STAT3 | 7.890863393 | 7.97970933 | 0.016153032 | 0.021187808 |
| SH2D1B | 3.752119632 | 3.690740887 | -0.023795372 | 0.022510029 |
| TBP | 7.666922431 | 7.76648803 | 0.018614782 | 0.023288648 |
| TGFB1 | 5.070941866 | 5.140186943 | 0.019567094 | 0.023471615 |
| SELPLG | 6.409365199 | 6.508120897 | 0.022059576 | 0.023655828 |
| LILRA5 | 4.340197801 | 4.289519753 | -0.016944658 | 0.024532169 |
| CLEC5A | 4.598912196 | 4.621352653 | 0.007022531 | 0.029273997 |
| CX3CR1 | 4.656455573 | 4.801773657 | 0.044335186 | 0.030633639 |
| CC2D1B | 5.55449151 | 5.50267127 | -0.013522701 | 0.033934378 |
| MASP1 | 4.309741377 | 4.342771613 | 0.011014786 | 0.034018534 |
| TOLLIP | 5.713995268 | 5.667787273 | -0.01171423 | 0.034442045 |
| PMCH | 4.694134697 | 4.60879477 | -0.026469715 | 0.035215706 |
| FUT5 | 5.292091378 | 5.244067083 | -0.013151832 | 0.037078778 |
| HDAC3 | 7.909755102 | 7.96859083 | 0.010691592 | 0.037351673 |
| CCL24 | 5.474510692 | 5.410008833 | -0.017099074 | 0.039692917 |
| IFIT2 | 7.187603014 | 6.983131977 | -0.041636489 | 0.041754709 |
| GNLY | 5.888622749 | 6.164619767 | 0.066081661 | 0.043385814 |
| IFNAR1 | 6.690777936 | 6.62604549 | -0.014025855 | 0.045500096 |
| GZMH | 6.67799904 | 6.50486702 | -0.037896322 | 0.04593321 |
| CTCFL | 2.839336583 | 2.85166663 | 0.006251455 | 0.046260289 |
| XCR1 | 3.541712384 | 3.591883657 | 0.020293563 | 0.046699516 |
| HAVCR2 | 5.326062254 | 5.265377913 | -0.01653221 | 0.047142216 |
| FAS | 6.727620565 | 6.52787855 | -0.043482124 | 0.049063265 |
| CD47 | 7.742835966 | 7.67161529 | -0.013331704 | 0.052857731 |
| CD19 | 5.979700911 | 5.815600937 | -0.040145051 | 0.053843737 |
| GUSB | 7.034461759 | 6.991303073 | -0.008878663 | 0.05384376 |
| IKBKB | 5.762071116 | 5.700737633 | -0.015438861 | 0.055606248 |
| IL1A | 4.419158029 | 4.3382547 | -0.026656766 | 0.057547705 |
| IL24 | 5.091804471 | 5.08813121 | -0.001041145 | 0.06022367 |
| CCL16 | 3.321313346 | 3.270665893 | -0.022169447 | 0.060360084 |
| USP39 | 8.021993381 | 8.093681707 | 0.012835339 | 0.06036012 |
| IFI35 | 8.075777063 | 7.964251817 | -0.020062247 | 0.065300188 |
| TNFSF13B | 8.619346247 | 8.420212163 | -0.033721864 | 0.069181921 |
| RIPK2 | 7.55074225 | 7.483337157 | -0.012936694 | 0.070568983 |
| MERTK | 5.165561018 | 5.097027417 | -0.019268931 | 0.071036362 |
| FCGR2A | 5.23084819 | 5.30705814 | 0.020867453 | 0.071663509 |
| IL17F | 3.740338121 | 3.799156023 | 0.022510268 | 0.07341155 |
| ATF1 | 7.898227096 | 7.83476168 | -0.011639459 | 0.07734662 |
| VEGFA | 7.400203868 | 7.358741147 | -0.008106029 | 0.079720335 |
| POU2F2 | 5.49279989 | 5.45569506 | -0.009778725 | 0.080755629 |
| GPI | 9.349034541 | 9.473773613 | 0.019121809 | 0.081626678 |
| PSEN2 | 6.165000776 | 6.109156013 | -0.013127992 | 0.082681983 |
| PYCARD | 9.134048305 | 8.961070317 | -0.02758336 | 0.087753837 |
| COL3A1 | 9.085142666 | 9.24485453 | 0.025141449 | 0.088872666 |
| CXCL6 | 6.876351916 | 6.29175611 | -0.128180632 | 0.089248153 |
| MSR1 | 4.67660963 | 4.814055613 | 0.0417898 | 0.091720066 |
| PAX5 | 5.264659523 | 5.16370443 | -0.027933809 | 0.09464032 |
| TBX21 | 4.693932668 | 4.6623269 | -0.009746984 | 0.094837623 |
| KIR3DL1 | 3.92646311 | 3.89896015 | -0.010140933 | 0.095233202 |
| IKBKG | 7.291063654 | 7.2636319 | -0.005438204 | 0.095233217 |
| C6 | 5.165837119 | 5.091172517 | -0.021004202 | 0.096628217 |
| TLR4 | 5.419791051 | 5.570349927 | 0.039530727 | 0.096628217 |
| FCF1 | 4.313518029 | 4.35655367 | 0.01432233 | 0.100290488 |
| ABCB1 | 4.969369031 | 5.070599313 | 0.029093593 | 0.10385008 |
| IL15 | 6.625782373 | 6.755370603 | 0.027944101 | 0.104275377 |
| G6PD | 6.010916262 | 5.970697027 | -0.009685559 | 0.104702072 |
| C2 | 5.351175576 | 5.271233503 | -0.021715264 | 0.109931052 |
| CXCL3 | 9.450295092 | 9.249359397 | -0.03100593 | 0.110822276 |
| TRAF3 | 7.196148549 | 7.274715557 | 0.015665871 | 0.115133741 |
| IL3 | 3.117939345 | 3.144343627 | 0.012166027 | 0.116057927 |
| PRAME | 4.839040866 | 4.908575657 | 0.020583329 | 0.118394171 |
| MAP2K2 | 5.486759401 | 5.511215293 | 0.006416173 | 0.118629839 |
| PIK3CG | 5.160541432 | 5.076987393 | -0.02354976 | 0.121486204 |
| LAMP1 | 9.297892164 | 9.34360249 | 0.007075204 | 0.129366334 |
| FLT3LG | 4.242555374 | 4.194354117 | -0.016484821 | 0.1354019 |
| BAGE | 2.908404785 | 2.892727133 | -0.007797832 | 0.143830552 |
| HMGB1 | 11.70092923 | 11.6872822 | -0.001683626 | 0.145757102 |
| CCL23 | 4.01177661 | 3.950579037 | -0.022177149 | 0.155832806 |
| NOD2 | 5.559612355 | 5.498341233 | -0.015987849 | 0.158027582 |
| CD244 | 4.175480846 | 4.22041964 | 0.015444107 | 0.159207632 |
| IL17A | 3.26342795 | 3.241936187 | -0.009532494 | 0.159800181 |
| TNFRSF10B | 6.307407734 | 6.369685053 | 0.014174843 | 0.176435691 |
| IFITM1 | 11.02303649 | 11.10100783 | 0.010168966 | 0.177075722 |
| ATG10 | 4.98274785 | 4.946802883 | -0.010445156 | 0.181931174 |
| CD40 | 6.067055906 | 6.04191179 | -0.005991484 | 0.186551395 |
| CD1A | 4.465544141 | 4.426098693 | -0.012800362 | 0.19024191 |
| CCR4 | 5.67370559 | 5.62473397 | -0.012506427 | 0.190918679 |
| GAGE1 | 3.070708328 | 3.17150859 | 0.046597766 | 0.204115303 |
| CCR1 | 6.880124565 | 6.98504042 | 0.021833779 | 0.209140964 |
| TAPBP | 6.61985793 | 6.579112637 | -0.008907243 | 0.219459731 |
| KIR3DL2 | 3.916804706 | 3.954583973 | 0.013848733 | 0.219834901 |
| PLA2G6 | 5.995501013 | 6.01999424 | 0.005881789 | 0.2266668 |
| MAF | 5.160693224 | 5.20450693 | 0.012196618 | 0.227050742 |
| MICB | 7.004373447 | 7.142340383 | 0.028140885 | 0.234827143 |
| NCF4 | 6.264934869 | 6.177114073 | -0.020366538 | 0.243596917 |
| IL1R1 | 6.367589997 | 6.464104193 | 0.021703008 | 0.248067053 |
| TRAF2 | 5.575305403 | 5.5237678 | -0.01339816 | 0.252594275 |
| CR1 | 4.264145704 | 4.340819443 | 0.02571068 | 0.253423553 |
| ARG2 | 5.93599074 | 6.00070363 | 0.015642837 | 0.254254725 |
| NOTCH1 | 5.886305479 | 5.82505737 | -0.015090156 | 0.259703579 |
| APOE | 7.896060716 | 7.798646867 | -0.017909258 | 0.267380802 |
| ULBP2 | 5.32631663 | 5.320174627 | -0.001664593 | 0.271277357 |
| IGF2R | 7.581838701 | 7.54052911 | -0.007882005 | 0.273240129 |
| ICAM3 | 8.384915331 | 8.490184773 | 0.017999736 | 0.274334756 |
| TNFRSF8 | 4.681051982 | 4.708833547 | 0.00853694 | 0.277636572 |
| CD163 | 7.137722915 | 7.053716643 | -0.017080275 | 0.281857653 |
| PTPRC | 6.564639653 | 6.452755933 | -0.024800362 | 0.284096647 |
| KIT | 7.942567625 | 8.090576333 | 0.026637008 | 0.287251415 |
| PLAUR | 8.19672891 | 8.089276697 | -0.019037575 | 0.288610669 |
| LTF | 8.319990763 | 8.872039793 | 0.09268391 | 0.289974252 |
| F12 | 5.63348695 | 5.662985193 | 0.007534577 | 0.2954719 |
| IRAK2 | 4.741603778 | 4.765186517 | 0.007157572 | 0.295933167 |
| MS4A2 | 4.029926996 | 4.00779389 | -0.007945387 | 0.308095324 |
| STAT6 | 7.453696365 | 7.395423853 | -0.011323216 | 0.358434161 |
| CCL26 | 4.233595222 | 4.394037483 | 0.053663838 | 0.361053642 |
| IL18RAP | 5.817541639 | 5.91729991 | 0.024529386 | 0.365269936 |
| TNFSF14 | 4.467126313 | 4.46013869 | -0.002258477 | 0.368984206 |
| TUBB | 10.87487917 | 10.90575336 | 0.004090062 | 0.372186493 |
| SPACA3 | 3.760310374 | 3.779886233 | 0.007491067 | 0.380267257 |
| IFNA8 | 2.705320629 | 2.687462203 | -0.009555125 | 0.385713784 |
| RAG1 | 2.962253337 | 2.994486457 | 0.01561358 | 0.393418744 |
| BTK | 6.048551363 | 6.00773845 | -0.00976765 | 0.409672479 |
| CCL5 | 8.762524228 | 8.702563713 | -0.009906057 | 0.429811812 |
| UBC | 13.19022069 | 13.17785079 | -0.001353606 | 0.44692595 |
| PDCD1 | 6.22558665 | 6.207326983 | -0.004237647 | 0.454119487 |
| TAP1 | 9.89903897 | 9.854793033 | -0.006462898 | 0.46198567 |
| IRF7 | 6.877384445 | 6.856533133 | -0.004380703 | 0.510103098 |
| ITGA5 | 6.414442724 | 6.37380679 | -0.009168645 | 0.523666773 |
| JAK1 | 6.947904496 | 6.955275823 | 0.001529805 | 0.530190104 |
| IL11 | 5.251775833 | 5.407465113 | 0.042147115 | 0.54137367 |
| IL32 | 7.07629436 | 7.011570057 | -0.013256529 | 0.558702719 |
| NOL7 | 8.623388355 | 8.636904287 | 0.002259449 | 0.566114506 |
| EOMES | 6.388473522 | 6.509012807 | 0.026967502 | 0.566790638 |
| CD7 | 4.239264607 | 4.264031807 | 0.00840418 | 0.573573162 |
| DPP4 | 7.108644099 | 6.801402047 | -0.063742232 | 0.57902673 |
| SPA17 | 7.281856927 | 7.30176448 | 0.00393874 | 0.599691395 |
| TNF | 4.350281157 | 4.41813825 | 0.022329919 | 0.605955278 |
| NLRC5 | 7.79084393 | 7.7621073 | -0.005331238 | 0.615054407 |
| IL18 | 7.222789452 | 7.13206337 | -0.018236594 | 0.619978699 |
| ZC3H14 | 6.335410155 | 6.333487163 | -0.000437969 | 0.62068357 |
| TNFSF15 | 6.208888061 | 6.203537887 | -0.001243701 | 0.627751354 |
| ATF2 | 5.878998767 | 5.88506561 | 0.001488024 | 0.644136571 |
| ITGAE | 9.752473348 | 9.736101777 | -0.002423901 | 0.654196193 |
| CEACAM6 | 10.49309295 | 10.17830381 | -0.04394283 | 0.667222642 |
| ITGB1 | 6.404279373 | 6.4109449 | 0.001500766 | 0.70912314 |
| SELL | 7.039996509 | 7.032956263 | -0.001443468 | 0.718062839 |
| IL11RA | 5.046543381 | 5.046508187 | -1.01E-05 | 0.721051409 |
| CD247 | 6.135435069 | 6.11597813 | -0.004582402 | 0.737563676 |
| TLR9 | 4.596088546 | 4.60445719 | 0.002624498 | 0.739824926 |
| LILRA4 | 4.981153718 | 4.9629733 | -0.00527524 | 0.748135439 |
| LBP | 4.029790911 | 4.07224789 | 0.0151204 | 0.757234925 |
| DDX58 | 7.230238609 | 7.206885573 | -0.00466732 | 0.774768947 |
| ETS1 | 7.119924571 | 7.096752473 | -0.00470297 | 0.79087947 |
| SMAD2 | 7.383968947 | 7.35523438 | -0.005625171 | 0.802442382 |
| SOCS1 | 4.756734471 | 4.776393767 | 0.005950284 | 0.802442382 |
| CSF1 | 5.267075752 | 5.272453783 | 0.001472335 | 0.812498781 |
| TNFRSF11B | 5.280322523 | 5.290171887 | 0.002688547 | 0.814823952 |
| CD274 | 6.017152488 | 6.053486843 | 0.008685464 | 0.843629098 |
| TXNIP | 10.35831193 | 10.31707805 | -0.005754474 | 0.846756465 |
| CCL13 | 4.955643989 | 4.9396385 | -0.004667084 | 0.856152867 |
| IFNA2 | 2.982492402 | 2.969139123 | -0.006473768 | 0.871858279 |
| BST1 | 5.850756259 | 5.823410737 | -0.006758739 | 0.871858302 |
| C3AR1 | 8.370978887 | 8.365232663 | -0.000990672 | 0.899461076 |
| C1QA | 7.651043205 | 7.682928157 | 0.005999793 | 0.90658001 |
| IL10RA | 7.911259492 | 7.907668507 | -0.000655 | 0.910538257 |
| CD55 | 10.30460313 | 10.21151985 | -0.013091333 | 0.914498725 |
| RPS6 | 10.97668715 | 10.97179659 | -0.000642922 | 0.924805638 |
| CYLD | 5.921287577 | 5.920352653 | -0.000227808 | 0.943070821 |
| FOXJ1 | 5.436437059 | 5.45744839 | 0.005565137 | 0.96057005 |
| JAK3 | 4.496060018 | 4.50533832 | 0.002974152 | 0.991632818 |
