# Supplementary material for: Immunogenomic characterization in gastric cancer identifies microenvironmental and immunotherapeutically relevant gene signatures
Source: Immun Inflamm Dis. 2021 Sep 28;10(1):43–59. doi: 10.1002/iid3.539 (PMC8669697; doi:10.1002/iid3.539)
Supplement: Supplementary file 6 — Supplementary information. [file IID3-10-43-s012.docx]

**Table-S5.** Gene Ontology (GO) enrichment analyses for immune-related genes in gastric cancer.

| **Term** | **Count** | **PValue** |
| --- | --- | --- |
| positive regulation of cytokine production | 179 | 1.79E-128 |
| T cell activation | 177 | 8.41E-127 |
| regulation of leukocyte activation | 176 | 2.46E-116 |
| regulation of lymphocyte activation | 157 | 4.85E-110 |
| leukocyte proliferation | 132 | 3.52E-107 |
| leukocyte cell-cell adhesion | 142 | 2E-106 |
| positive regulation of cell activation | 139 | 1.4E-104 |
| positive regulation of leukocyte activation | 136 | 6.31E-104 |
| regulation of leukocyte cell-cell adhesion | 132 | 5.48E-101 |
| leukocyte differentiation | 161 | 3E-100 |
| regulation of cell-cell adhesion | 145 | 7.27E-98 |
| mononuclear cell proliferation | 120 | 3.87E-97 |
| regulation of T cell activation | 131 | 5.17E-97 |
| lymphocyte proliferation | 119 | 2.43E-96 |
| positive regulation of lymphocyte activation | 121 | 1.82E-95 |
| regulation of immune effector process | 150 | 6.99E-95 |
| leukocyte migration | 149 | 1.2E-89 |
| positive regulation of leukocyte cell-cell adhesion | 108 | 3.01E-89 |
| positive regulation of cell-cell adhesion | 113 | 2.01E-86 |
| positive regulation of cell adhesion | 134 | 3.43E-85 |
| regulation of leukocyte proliferation | 105 | 4.78E-85 |
| lymphocyte mediated immunity | 119 | 7.96E-85 |
| regulation of mononuclear cell proliferation | 100 | 6.24E-82 |
| regulation of innate immune response | 138 | 1.34E-81 |
| positive regulation of T cell activation | 99 | 4.11E-81 |
| regulation of lymphocyte proliferation | 99 | 7.59E-81 |
| lymphocyte differentiation | 117 | 2.79E-76 |
| defense response to other organism | 138 | 5.97E-76 |
| response to molecule of bacterial origin | 114 | 1.23E-75 |
| positive regulation of response to external stimulus | 113 | 2.2E-73 |
| regulation of inflammatory response | 134 | 4.23E-72 |
| positive regulation of innate immune response | 119 | 8.81E-71 |
| positive regulation of leukocyte proliferation | 78 | 1.87E-70 |
