# Supplementary material for: Immunogenomic characterization in gastric cancer identifies microenvironmental and immunotherapeutically relevant gene signatures
Source: Immun Inflamm Dis. 2021 Sep 28;10(1):43–59. doi: 10.1002/iid3.539 (PMC8669697; doi:10.1002/iid3.539)
Supplement: Supplementary file 7 — Supplementary information. [file IID3-10-43-s013.docx]

**Table-S6.** Estimating relative abundance of TME infiltrating cells in ACRG cohort using the Single-Sample Gene-Set Enrichment Analysis (ssGSEA).

| **ID** | **IGPC1** | **IGPC2** | **IGPC3** |
| --- | --- | --- | --- |
| Activated B cell | -0.102901 | 0.038167104 | 0.09658626 |
| Activated CD4 T cell | 0.375097523 | 0.499574263 | 0.399773109 |
| Activated CD8 T cell | 0.330473204 | 0.462869902 | 0.423185885 |
| B.cells.naive | -0.121951901 | -0.067241892 | -0.023975181 |
| Dendritic.cells.activated | 0.013421932 | 0.121922068 | 0.121564963 |
| Dendritic.cells.resting | 0.08841204 | 0.141583396 | 0.159293028 |
| Endothelial cells | -0.012823476 | 0.047251139 | 0.094063516 |
| Eosinophil | -0.008508992 | 0.068548336 | 0.106462603 |
| Fibroblasts | 0.389357336 | 0.483311701 | 0.531775639 |
| Gamma delta T cell | 0.402952774 | 0.445172878 | 0.412792704 |
| Immature dendritic cell | 0.372752076 | 0.41284891 | 0.406132071 |
| Macrophage | 0.042991516 | 0.145918728 | 0.097466645 |
| Mast cell | 0.102904233 | 0.206015788 | 0.182692814 |
| Mast.cells.resting | -0.115823172 | -0.049668976 | -0.015356502 |
| MDSC | 0.298811825 | 0.470519027 | 0.421577144 |
| Monocytes | -0.181567129 | -0.058113702 | -0.086145717 |
| Natural killer cell | 0.206895295 | 0.274840813 | 0.2611267 |
| Natural killer T cell | 0.108118635 | 0.163927975 | 0.124285516 |
| Neutrophil | -0.076018632 | 0.046960606 | -0.082268872 |
| NK.cells.resting | -0.147821584 | -0.123171813 | -0.128268376 |
| Plasma.cells | -0.033649432 | 0.009042902 | 0.013992741 |
| Plasmacytoid dendritic cell | 0.311288431 | 0.355101072 | 0.33609683 |
| Regulatory T cell | 0.183712726 | 0.327813082 | 0.258167004 |
| T follicular helper cell | 0.150291324 | 0.227737344 | 0.217753975 |
