# Supplementary material for: Immunogenomic characterization in gastric cancer identifies microenvironmental and immunotherapeutically relevant gene signatures
Source: Immun Inflamm Dis. 2021 Sep 28;10(1):43–59. doi: 10.1002/iid3.539 (PMC8669697; doi:10.1002/iid3.539)
Supplement: Supplementary file 8 — Supplementary information. [file IID3-10-43-s011.docx]

| **Pathway** | **IGPC1** | **IGPC3** | **pValue** |
| --- | --- | --- | --- |
| APOPTOSIS | -0.119501627 | -0.034548134 | 0.004316923 |
| B_CELL_RECEPTOR_SIGNALING_PATHWAY | -0.195262519 | 0.060461985 | 1.74E-14 |
| BASE_EXCISION_REPAIR | 0.09488235 | -0.268943219 | 2.95E-15 |
| CELL_ADHESION_MOLECULES_CAMS | -0.240144453 | 0.175359108 | 5.02E-27 |
| CELL_CYCLE | 0.053238037 | -0.250217457 | 4.59E-13 |
| CHEMOKINE_SIGNALING_PATHWAY | -0.2102914 | 0.069854404 | 6.42E-20 |
| COLORECTAL_CANCER | -0.08847282 | 0.047960267 | 4.08E-07 |
| ECM_RECEPTOR_INTERACTION | -0.176225767 | 0.130016918 | 1.16E-11 |
| FOCAL_ADHESION | -0.142781158 | 0.117429567 | 2.20E-13 |
| JAK_STAT_SIGNALING_PATHWAY | -0.164268149 | -0.004536502 | 3.52E-11 |
| MAPK_SIGNALING_PATHWAY | -0.087132121 | 0.046597606 | 1.24E-13 |
| MISMATCH_REPAIR | 0.074024804 | -0.292954313 | 1.50E-11 |
| MTOR_SIGNALING_PATHWAY | -0.077321715 | 0.057408414 | 1.87E-06 |
| NATURAL_KILLER_CELL_MEDIATED_CYTOTOXICITY | -0.191651572 | -0.030861109 | 3.84E-09 |
| PATHWAYS_IN_CANCER | -0.075761829 | 0.017333293 | 7.18E-08 |
| T_CELL_RECEPTOR_SIGNALING_PATHWAY | -0.162969803 | -0.001003178 | 4.22E-09 |
| TGF_BETA_SIGNALING_PATHWAY | -0.095225891 | 0.11337433 | 1.08E-11 |
| TOLL_LIKE_RECEPTOR_SIGNALING_PATHWAY | -0.186009571 | -0.041932667 | 6.25E-09 |
| VEGF_SIGNALING_PATHWAY | -0.059107208 | -0.007029272 | 0.012196108 |
| WNT_SIGNALING_PATHWAY | -0.010503184 | 0.047877387 | 0.00228809 |
| **Pathway** | **IGPC2** | **IGPC3** | **pValue** |
| ANTIGEN_PROCESSING_AND_PRESENTATION | 0.275612475 | -0.050377624 | 9.76E-14 |
| APOPTOSIS | 0.186749203 | -0.034548134 | 1.57E-12 |
| CHEMOKINE_SIGNALING_PATHWAY | 0.20319455 | 0.069854404 | 1.98E-06 |
| CYTOKINE_CYTOKINE_RECEPTOR_INTERACTION | 0.21517875 | 0.044068415 | 5.54E-09 |
| FC_EPSILON_RI_SIGNALING_PATHWAY | 0.128294258 | 0.046233075 | 0.000648575 |
| GRAFT_VERSUS_HOST_DISEASE | 0.392891615 | -0.007722082 | 2.23E-14 |
| JAK_STAT_SIGNALING_PATHWAY | 0.187648173 | -0.004536502 | 3.33E-13 |
| NATURAL_KILLER_CELL_MEDIATED_CYTOTOXICITY | 0.239608862 | -0.030861109 | 5.50E-16 |
| NOD_LIKE_RECEPTOR_SIGNALING_PATHWAY | 0.277588686 | -0.10209123 | 1.20E-22 |
| PPAR_SIGNALING_PATHWAY | -0.027267862 | 0.093451409 | 1.37E-05 |
| RIG_I_LIKE_RECEPTOR_SIGNALING_PATHWAY | 0.178551592 | -0.098516306 | 1.36E-18 |
| T_CELL_RECEPTOR_SIGNALING_PATHWAY | 0.192430284 | -0.001003178 | 6.78E-11 |
| TGF_BETA_SIGNALING_PATHWAY | 0.037493042 | 0.11337433 | 0.011734666 |
| TOLL_LIKE_RECEPTOR_SIGNALING_PATHWAY | 0.247719991 | -0.041932667 | 4.80E-20 |
| WNT_SIGNALING_PATHWAY | -0.021886361 | 0.047877387 | 0.001015969 |

**Table-S7.** The activiated biological pathways in distinct immunogenomic phenotypes revealed by GSVA enrichment analysis.
