# Supplementary material for: Immunogenomic characterization in gastric cancer identifies microenvironmental and immunotherapeutically relevant gene signatures
Source: Immun Inflamm Dis. 2021 Sep 28;10(1):43–59. doi: 10.1002/iid3.539 (PMC8669697; doi:10.1002/iid3.539)
Supplement: Supplementary file 9 — Supplementary information. [file IID3-10-43-s006.docx]

**Table-S8.** Spearman correlation between immune genes with high frequency mutation and TME infiltrating cells in gastric cancer.

| **From** | **To** | P value（<0.00001） | **Cor** |
| --- | --- | --- | --- |
| IRF2 | Activated.B.cell | 2.7492E-14 | 0.420555207 |
| IRF2 | Activated.CD4.T.cell | 1.1443E-17 | 0.467153502 |
| IRF2 | Activated.CD8.T.cell | 1.44125E-33 | 0.62240527 |
| IRF2 | B.cells.naive | 0.000120062 | 0.220235887 |
| IRF2 | Dendritic.cells.activated | 1.29149E-17 | 0.466481094 |
| IRF2 | Dendritic.cells.resting | 2.41289E-06 | 0.268351737 |
| IRF2 | Endothelial.cells | 0.011943779 | 0.144972073 |
| IRF2 | Eosinophil | 1.49948E-08 | 0.319595647 |
| IRF2 | Fibroblasts | 0.040552938 | 0.118323767 |
| IRF2 | Gamma.delta.T.cell | 6.02849E-09 | 0.327828731 |
| IRF2 | Immature.dendritic.cell | 6.94955E-08 | 0.305151307 |
| IRF2 | Macrophage | 8.56919E-16 | 0.442203326 |
| IRF2 | Mast.cell | 7.05154E-05 | 0.227405978 |
| IRF2 | Mast.cells.resting | 0.001214833 | 0.185939101 |
| IRF2 | MDSC | 1.12431E-27 | 0.573887972 |
| IRF2 | Monocytes | 2.90715E-10 | 0.353593438 |
| IRF2 | Natural.killer.cell | 1.64479E-21 | 0.512692924 |
| IRF2 | Natural.killer.T.cell | 2.04511E-12 | 0.391289492 |
| IRF2 | Neutrophil | 1.64008E-07 | 0.29671005 |
| IRF2 | NK.cells.resting | 1.02162E-17 | 0.467782328 |
| IRF2 | Plasma.cells | 4.49112E-06 | 0.261319977 |
| IRF2 | Plasmacytoid.dendritic.cell | 0.011578546 | 0.145595937 |
| IRF2 | Regulatory.T.cell | 3.1351E-12 | 0.388225187 |
| IRF2 | T.follicular.helper.cell | 1.18418E-14 | 0.42595637 |
| NLRC5 | Activated.B.cell | 1.56765E-13 | 0.409074893 |
| NLRC5 | Activated.CD4.T.cell | 7.00566E-31 | 0.601301056 |
| NLRC5 | Activated.CD8.T.cell | 8.59387E-46 | 0.70174205 |
| NLRC5 | B.cells.naive | 3.21097E-06 | 0.265142422 |
| NLRC5 | Dendritic.cells.activated | 1.51919E-16 | 0.45245071 |
| NLRC5 | Dendritic.cells.resting | 3.14037E-05 | 0.237887101 |
| NLRC5 | Endothelial.cells | 0.074623215 | 0.10308444 |
| NLRC5 | Eosinophil | 5.4814E-07 | 0.284369557 |
| NLRC5 | Fibroblasts | 0.01253775 | 0.143992541 |
| NLRC5 | Gamma.delta.T.cell | 1.15936E-07 | 0.300152545 |
| NLRC5 | Immature.dendritic.cell | 3.07024E-06 | 0.265648539 |
| NLRC5 | Macrophage | 3.34218E-13 | 0.403942015 |
| NLRC5 | Mast.cell | 0.001718016 | 0.18027189 |
| NLRC5 | Mast.cells.resting | 0.245089105 | 0.06731378 |
| NLRC5 | MDSC | 2.1841E-27 | 0.57128976 |
| NLRC5 | Monocytes | 1.568E-07 | 0.297158582 |
| NLRC5 | Natural.killer.cell | 1.79865E-23 | 0.533547272 |
| NLRC5 | Natural.killer.T.cell | 8.40776E-18 | 0.468859641 |
| NLRC5 | Neutrophil | 3.89396E-05 | 0.235146616 |
| NLRC5 | NK.cells.resting | 3.83476E-16 | 0.447009024 |
| NLRC5 | Plasma.cells | 0.000894673 | 0.190810644 |
| NLRC5 | Plasmacytoid.dendritic.cell | 0.000101194 | 0.222564558 |
| NLRC5 | Regulatory.T.cell | 6.58739E-15 | 0.42966111 |
| NLRC5 | T.follicular.helper.cell | 4.30569E-16 | 0.446321387 |
| TP53 | Activated.B.cell | 0.705261086 | 0.021925923 |
| TP53 | Activated.CD4.T.cell | 0.000816503 | 0.192244078 |
| TP53 | Activated.CD8.T.cell | 0.001146756 | 0.186866593 |
| TP53 | B.cells.naive | 0.348803684 | -0.054279366 |
| TP53 | Dendritic.cells.activated | 0.876973083 | 0.008975103 |
| TP53 | Dendritic.cells.resting | 0.889665348 | 0.008042893 |
| TP53 | Endothelial.cells | 0.021597267 | -0.132606438 |
| TP53 | Eosinophil | 0.260305298 | -0.065193579 |
| TP53 | Fibroblasts | 0.051211736 | -0.112677724 |
| TP53 | Gamma.delta.T.cell | 0.603003904 | 0.030146182 |
| TP53 | Immature.dendritic.cell | 0.005625536 | -0.159500557 |
| TP53 | Macrophage | 0.813085311 | -0.013707897 |
| TP53 | Mast.cell | 0.040286758 | -0.118480062 |
| TP53 | Mast.cells.resting | 0.001109051 | -0.187402316 |
| TP53 | MDSC | 0.609090005 | 0.02964138 |
| TP53 | Monocytes | 0.458030081 | -0.043004551 |
| TP53 | Natural.killer.cell | 0.491355873 | 0.039881265 |
| TP53 | Natural.killer.T.cell | 0.519646539 | 0.037317865 |
| TP53 | Neutrophil | 0.69027361 | -0.023099836 |
| TP53 | NK.cells.resting | 0.083600178 | 0.100058211 |
| TP53 | Plasma.cells | 0.879655711 | -0.008777887 |
| TP53 | Plasmacytoid.dendritic.cell | 0.001370967 | -0.183980336 |
| TP53 | Regulatory.T.cell | 0.653163289 | -0.02604885 |
| TP53 | T.follicular.helper.cell | 0.541813699 | -0.035359156 |
| HLA-B | Activated.B.cell | 2.02462E-06 | 0.270301885 |
| HLA-B | Activated.CD4.T.cell | 9.74856E-18 | 0.46804179 |
| HLA-B | Activated.CD8.T.cell | 7.53719E-19 | 0.481891239 |
| HLA-B | B.cells.naive | 0.003337351 | 0.168928075 |
| HLA-B | Dendritic.cells.activated | 1.95396E-08 | 0.317156509 |
| HLA-B | Dendritic.cells.resting | 5.28615E-08 | 0.307785577 |
| HLA-B | Endothelial.cells | 0.922452122 | 0.005643754 |
| HLA-B | Eosinophil | 0.003153743 | 0.169921603 |
| HLA-B | Fibroblasts | 0.886796946 | -0.008253379 |
| HLA-B | Gamma.delta.T.cell | 1.35693E-14 | 0.425089649 |
| HLA-B | Immature.dendritic.cell | 0.000502976 | 0.199676795 |
| HLA-B | Macrophage | 2.18559E-08 | 0.316117762 |
| HLA-B | Mast.cell | 0.077224836 | 0.102178354 |
| HLA-B | Mast.cells.resting | 0.750683728 | 0.018419949 |
| HLA-B | MDSC | 1.47512E-16 | 0.45262212 |
| HLA-B | Monocytes | 1.94208E-05 | 0.243896111 |
| HLA-B | Natural.killer.cell | 2.58288E-08 | 0.314561665 |
| HLA-B | Natural.killer.T.cell | 3.64494E-11 | 0.369982117 |
| HLA-B | Neutrophil | 3.12445E-09 | 0.333618316 |
| HLA-B | NK.cells.resting | 3.2154E-09 | 0.333368073 |
| HLA-B | Plasma.cells | 4.78698E-07 | 0.285784576 |
| HLA-B | Plasmacytoid.dendritic.cell | 0.01744907 | 0.137165612 |
| HLA-B | Regulatory.T.cell | 7.29957E-11 | 0.36460373 |
| HLA-B | T.follicular.helper.cell | 6.15122E-08 | 0.306329464 |
| TTK | Activated.B.cell | 1.70121E-10 | -0.357912786 |
| TTK | Activated.CD4.T.cell | 6.13275E-11 | 0.365962028 |
| TTK | Activated.CD8.T.cell | 0.036719159 | -0.120663887 |
| TTK | B.cells.naive | 1.56291E-10 | -0.358590337 |
| TTK | Dendritic.cells.activated | 9.62429E-15 | -0.427271354 |
| TTK | Dendritic.cells.resting | 0.000157986 | -0.216444927 |
| TTK | Endothelial.cells | 1.35872E-24 | -0.544857764 |
| TTK | Eosinophil | 4.08503E-17 | -0.46000685 |
| TTK | Fibroblasts | 1.16635E-19 | -0.491605009 |
| TTK | Gamma.delta.T.cell | 0.020343172 | 0.133898101 |
| TTK | Immature.dendritic.cell | 0.00011066 | -0.22134967 |
| TTK | Macrophage | 0.012467909 | -0.144105568 |
| TTK | Mast.cell | 1.0624E-11 | -0.379291457 |
| TTK | Mast.cells.resting | 4.11332E-18 | -0.472780718 |
| TTK | MDSC | 3.60706E-05 | -0.236125513 |
| TTK | Monocytes | 0.000570372 | -0.197773261 |
| TTK | Natural.killer.cell | 1.59315E-11 | -0.376264413 |
| TTK | Natural.killer.T.cell | 0.56444582 | -0.033399993 |
| TTK | Neutrophil | 0.112661239 | 0.091777377 |
| TTK | NK.cells.resting | 2.86616E-06 | -0.266423261 |
| TTK | Plasma.cells | 0.882670592 | 0.008556366 |
| TTK | Plasmacytoid.dendritic.cell | 1.03188E-07 | -0.301298594 |
| TTK | Regulatory.T.cell | 0.112269539 | -0.091877044 |
| TTK | T.follicular.helper.cell | 1.48199E-11 | -0.376807047 |
| MAGEC1 | Activated.B.cell | 0.041801812 | -0.117601802 |
| MAGEC1 | Activated.CD4.T.cell | 0.766167392 | 0.017240622 |
| MAGEC1 | Activated.CD8.T.cell | 0.041471461 | -0.117790988 |
| MAGEC1 | B.cells.naive | 0.559250043 | -0.03384634 |
| MAGEC1 | Dendritic.cells.activated | 0.000820109 | -0.192175206 |
| MAGEC1 | Dendritic.cells.resting | 0.011820599 | -0.145180574 |
| MAGEC1 | Endothelial.cells | 0.002343765 | -0.175049374 |
| MAGEC1 | Eosinophil | 0.000506817 | -0.199562106 |
| MAGEC1 | Fibroblasts | 0.081039674 | -0.100893466 |
| MAGEC1 | Gamma.delta.T.cell | 0.410627929 | -0.047675763 |
| MAGEC1 | Immature.dendritic.cell | 0.189515824 | -0.075956716 |
| MAGEC1 | Macrophage | 0.010357059 | -0.147816949 |
| MAGEC1 | Mast.cell | 0.050309523 | -0.113115367 |
| MAGEC1 | Mast.cells.resting | 0.279352382 | -0.062656511 |
| MAGEC1 | MDSC | 0.037699462 | -0.120046532 |
| MAGEC1 | Monocytes | 0.805658252 | 0.014263428 |
| MAGEC1 | Natural.killer.cell | 0.002961826 | -0.171017821 |
| MAGEC1 | Natural.killer.T.cell | 0.301531127 | -0.059845619 |
| MAGEC1 | Neutrophil | 0.033845464 | -0.122557863 |
| MAGEC1 | NK.cells.resting | 5.73875E-05 | -0.230121218 |
| MAGEC1 | Plasma.cells | 0.421157304 | -0.046612267 |
| MAGEC1 | Plasmacytoid.dendritic.cell | 0.044138355 | -0.116298492 |
| MAGEC1 | Regulatory.T.cell | 0.437244328 | -0.045016932 |
| MAGEC1 | T.follicular.helper.cell | 0.010959381 | -0.146694397 |
| CDH1 | Activated.B.cell | 0.063278204 | -0.107365392 |
| CDH1 | Activated.CD4.T.cell | 0.009171338 | 0.150207055 |
| CDH1 | Activated.CD8.T.cell | 0.311603986 | -0.058614341 |
| CDH1 | B.cells.naive | 0.020065154 | -0.134193776 |
| CDH1 | Dendritic.cells.activated | 0.000315933 | -0.206569312 |
| CDH1 | Dendritic.cells.resting | 0.33536007 | 0.055810027 |
| CDH1 | Endothelial.cells | 2.33024E-09 | -0.336163508 |
| CDH1 | Eosinophil | 0.001063081 | -0.188078623 |
| CDH1 | Fibroblasts | 1.30961E-13 | -0.410281431 |
| CDH1 | Gamma.delta.T.cell | 0.00284166 | 0.171737515 |
| CDH1 | Immature.dendritic.cell | 0.000625604 | -0.196362876 |
| CDH1 | Macrophage | 0.014039022 | -0.141687784 |
| CDH1 | Mast.cell | 1.85419E-05 | -0.244467081 |
| CDH1 | Mast.cells.resting | 1.60284E-08 | -0.318983549 |
| CDH1 | MDSC | 0.016134786 | -0.138808359 |
| CDH1 | Monocytes | 0.019391573 | -0.134925163 |
| CDH1 | Natural.killer.cell | 9.12955E-07 | -0.278969295 |
| CDH1 | Natural.killer.T.cell | 0.049547356 | -0.113490207 |
| CDH1 | Neutrophil | 0.164733084 | 0.080418633 |
| CDH1 | NK.cells.resting | 0.571813839 | 0.032770393 |
| CDH1 | Plasma.cells | 0.000500117 | 0.199762679 |
| CDH1 | Plasmacytoid.dendritic.cell | 1.0512E-06 | -0.277456314 |
| CDH1 | Regulatory.T.cell | 0.130072611 | -0.087597791 |
| CDH1 | T.follicular.helper.cell | 2.88184E-05 | -0.238972743 |
| NOTCH1 | Activated.B.cell | 0.00569606 | -0.159269572 |
| NOTCH1 | Activated.CD4.T.cell | 0.122216884 | 0.089425972 |
| NOTCH1 | Activated.CD8.T.cell | 0.657286117 | -0.025718033 |
| NOTCH1 | B.cells.naive | 0.053588735 | -0.111554686 |
| NOTCH1 | Dendritic.cells.activated | 0.003532652 | -0.167924222 |
| NOTCH1 | Dendritic.cells.resting | 0.027055138 | -0.127646389 |
| NOTCH1 | Endothelial.cells | 0.625574699 | -0.028285111 |
| NOTCH1 | Eosinophil | 0.318773473 | -0.057753801 |
| NOTCH1 | Fibroblasts | 0.430131842 | 0.045718012 |
| NOTCH1 | Gamma.delta.T.cell | 0.493946007 | -0.039643395 |
| NOTCH1 | Immature.dendritic.cell | 0.000983722 | -0.189312179 |
| NOTCH1 | Macrophage | 0.811956287 | 0.013792264 |
| NOTCH1 | Mast.cell | 0.948143677 | 0.003770704 |
| NOTCH1 | Mast.cells.resting | 0.000312675 | -0.206720392 |
| NOTCH1 | MDSC | 0.904528121 | 0.006953872 |
| NOTCH1 | Monocytes | 0.89893178 | -0.007363618 |
| NOTCH1 | Natural.killer.cell | 0.173682274 | 0.07875331 |
| NOTCH1 | Natural.killer.T.cell | 0.020064299 | 0.13419469 |
| NOTCH1 | Neutrophil | 0.359589008 | 0.053078148 |
| NOTCH1 | NK.cells.resting | 0.427897592 | -0.045939621 |
| NOTCH1 | Plasma.cells | 0.003693738 | -0.167133365 |
| NOTCH1 | Plasmacytoid.dendritic.cell | 0.077387729 | 0.10212245 |
| NOTCH1 | Regulatory.T.cell | 0.081480815 | 0.100748059 |
| NOTCH1 | T.follicular.helper.cell | 0.364049892 | 0.052587915 |
| PIK3CG | Activated.B.cell | 6.11226E-53 | 0.738490648 |
| PIK3CG | Activated.CD4.T.cell | 3.79718E-14 | 0.418458293 |
| PIK3CG | Activated.CD8.T.cell | 1.3179E-33 | 0.622699106 |
| PIK3CG | B.cells.naive | 1.0699E-34 | 0.630820298 |
| PIK3CG | Dendritic.cells.activated | 6.8561E-28 | 0.575808372 |
| PIK3CG | Dendritic.cells.resting | 4.83864E-20 | 0.496080116 |
| PIK3CG | Endothelial.cells | 5.14825E-19 | 0.483900904 |
| PIK3CG | Eosinophil | 2.64753E-27 | 0.570532535 |
| PIK3CG | Fibroblasts | 5.24844E-13 | 0.40083962 |
| PIK3CG | Gamma.delta.T.cell | 3.48552E-13 | 0.403654692 |
| PIK3CG | Immature.dendritic.cell | 3.18846E-25 | 0.55102266 |
| PIK3CG | Macrophage | 1.30599E-24 | 0.545027801 |
| PIK3CG | Mast.cell | 1.63853E-18 | 0.477755922 |
| PIK3CG | Mast.cells.resting | 1.00365E-22 | 0.525776495 |
| PIK3CG | MDSC | 7.24193E-44 | 0.690771542 |
| PIK3CG | Monocytes | 2.61662E-32 | 0.61271304 |
| PIK3CG | Natural.killer.cell | 1.1265E-41 | 0.677658404 |
| PIK3CG | Natural.killer.T.cell | 1.29517E-14 | 0.425386434 |
| PIK3CG | Neutrophil | 3.41417E-05 | 0.236825887 |
| PIK3CG | NK.cells.resting | 1.59673E-10 | 0.35841941 |
| PIK3CG | Plasma.cells | 5.17081E-16 | 0.44523129 |
| PIK3CG | Plasmacytoid.dendritic.cell | 1.97583E-15 | 0.437128598 |
| PIK3CG | Regulatory.T.cell | 1.36314E-26 | 0.564003352 |
| PIK3CG | T.follicular.helper.cell | 1.01031E-44 | 0.69570604 |
| TLR4 | Activated.B.cell | 6.48736E-11 | 0.365524343 |
| TLR4 | Activated.CD4.T.cell | 2.63346E-29 | 0.588156105 |
| TLR4 | Activated.CD8.T.cell | 1.68775E-29 | 0.589801411 |
| TLR4 | B.cells.naive | 6.11927E-10 | 0.347483321 |
| TLR4 | Dendritic.cells.activated | 7.01403E-39 | 0.659891837 |
| TLR4 | Dendritic.cells.resting | 2.88236E-15 | 0.434806542 |
| TLR4 | Endothelial.cells | 1.1608E-11 | 0.378632375 |
| TLR4 | Eosinophil | 7.56876E-22 | 0.516381211 |
| TLR4 | Fibroblasts | 5.60123E-15 | 0.430677574 |
| TLR4 | Gamma.delta.T.cell | 1.21737E-32 | 0.615304231 |
| TLR4 | Immature.dendritic.cell | 1.4917E-32 | 0.614618389 |
| TLR4 | Macrophage | 5.16901E-82 | 0.842408063 |
| TLR4 | Mast.cell | 4.08334E-42 | 0.680350912 |
| TLR4 | Mast.cells.resting | 1.07611E-13 | 0.411593355 |
| TLR4 | MDSC | 6.81911E-66 | 0.792253722 |
| TLR4 | Monocytes | 4.36463E-37 | 0.647810224 |
| TLR4 | Natural.killer.cell | 2.13597E-44 | 0.693842135 |
| TLR4 | Natural.killer.T.cell | 1.63513E-37 | 0.650732221 |
| TLR4 | Neutrophil | 3.02363E-31 | 0.604262057 |
| TLR4 | NK.cells.resting | 5.04603E-05 | 0.231800435 |
| TLR4 | Plasma.cells | 0.004407975 | 0.163964454 |
| TLR4 | Plasmacytoid.dendritic.cell | 9.35338E-33 | 0.616191135 |
| TLR4 | Regulatory.T.cell | 1.01076E-79 | 0.836182619 |
| TLR4 | T.follicular.helper.cell | 3.79871E-49 | 0.719723915 |
| TPTE | Activated.B.cell | 0.009346362 | -0.149837594 |
| TPTE | Activated.CD4.T.cell | 0.188452919 | -0.076138943 |
| TPTE | Activated.CD8.T.cell | 0.000803546 | -0.192493831 |
| TPTE | B.cells.naive | 0.732834115 | 0.019788994 |
| TPTE | Dendritic.cells.activated | 0.000317142 | -0.206513613 |
| TPTE | Dendritic.cells.resting | 0.000694655 | -0.194753477 |
| TPTE | Endothelial.cells | 0.055802139 | -0.110545561 |
| TPTE | Eosinophil | 0.001516196 | -0.182334243 |
| TPTE | Fibroblasts | 0.136899218 | -0.086077144 |
| TPTE | Gamma.delta.T.cell | 0.316289288 | -0.058050527 |
| TPTE | Immature.dendritic.cell | 0.382907256 | -0.050555651 |
| TPTE | Macrophage | 0.00959804 | -0.149316945 |
| TPTE | Mast.cell | 0.054195327 | -0.111274741 |
| TPTE | Mast.cells.resting | 0.647623157 | -0.026494728 |
| TPTE | MDSC | 0.004269289 | -0.164541553 |
| TPTE | Monocytes | 0.518417996 | 0.037427651 |
| TPTE | Natural.killer.cell | 0.002364155 | -0.174901681 |
| TPTE | Natural.killer.T.cell | 0.01311697 | -0.143075946 |
| TPTE | Neutrophil | 0.03357111 | -0.122745751 |
| TPTE | NK.cells.resting | 1.79749E-06 | -0.271615956 |
| TPTE | Plasma.cells | 0.048339429 | -0.114094253 |
| TPTE | Plasmacytoid.dendritic.cell | 0.042943766 | -0.116957376 |
| TPTE | Regulatory.T.cell | 0.155295074 | -0.082250073 |
| TPTE | T.follicular.helper.cell | 0.004533531 | -0.163455941 |
| IGF2R | Activated.B.cell | 0.012469425 | -0.144103109 |
| IGF2R | Activated.CD4.T.cell | 0.031284854 | -0.124364501 |
| IGF2R | Activated.CD8.T.cell | 6.49984E-05 | -0.228483662 |
| IGF2R | B.cells.naive | 0.295358995 | -0.060613584 |
| IGF2R | Dendritic.cells.activated | 0.010239813 | -0.148042158 |
| IGF2R | Dendritic.cells.resting | 0.663234625 | -0.025242184 |
| IGF2R | Endothelial.cells | 0.246256013 | 0.067147998 |
| IGF2R | Eosinophil | 0.293875643 | -0.060799738 |
| IGF2R | Fibroblasts | 0.042365228 | -0.117282029 |
| IGF2R | Gamma.delta.T.cell | 0.267312108 | 0.064245972 |
| IGF2R | Immature.dendritic.cell | 0.492200554 | 0.039803619 |
| IGF2R | Macrophage | 0.894817216 | 0.007665106 |
| IGF2R | Mast.cell | 0.597994552 | -0.030563387 |
| IGF2R | Mast.cells.resting | 0.443725251 | -0.044383773 |
| IGF2R | MDSC | 0.284822545 | -0.061949708 |
| IGF2R | Monocytes | 0.180091553 | 0.077599463 |
| IGF2R | Natural.killer.cell | 0.12739165 | -0.088211845 |
| IGF2R | Natural.killer.T.cell | 0.513810112 | 0.03784062 |
| IGF2R | Neutrophil | 0.003264059 | 0.169318575 |
| IGF2R | NK.cells.resting | 0.174054806 | -0.078685387 |
| IGF2R | Plasma.cells | 0.489722476 | -0.040031617 |
| IGF2R | Plasmacytoid.dendritic.cell | 0.000351262 | 0.205017807 |
| IGF2R | Regulatory.T.cell | 0.511169602 | -0.038078133 |
| IGF2R | T.follicular.helper.cell | 0.980524935 | -0.001415266 |
| MRC1 | Activated.B.cell | 4.9511E-14 | 0.416724383 |
| MRC1 | Activated.CD4.T.cell | 7.62165E-17 | 0.456442251 |
| MRC1 | Activated.CD8.T.cell | 1.7234E-24 | 0.543834363 |
| MRC1 | B.cells.naive | 3.22794E-14 | 0.41951467 |
| MRC1 | Dendritic.cells.activated | 4.31544E-35 | 0.633698563 |
| MRC1 | Dendritic.cells.resting | 2.22395E-14 | 0.421923976 |
| MRC1 | Endothelial.cells | 4.78419E-17 | 0.459107792 |
| MRC1 | Eosinophil | 6.67336E-15 | 0.429579691 |
| MRC1 | Fibroblasts | 6.2449E-27 | 0.56713158 |
| MRC1 | Gamma.delta.T.cell | 4.86234E-26 | 0.55883486 |
| MRC1 | Immature.dendritic.cell | 9.13903E-37 | 0.645589453 |
| MRC1 | Macrophage | 8.78672E-51 | 0.728010039 |
| MRC1 | Mast.cell | 1.67202E-39 | 0.663958788 |
| MRC1 | Mast.cells.resting | 7.85944E-22 | 0.516203165 |
| MRC1 | MDSC | 5.52264E-59 | 0.765382953 |
| MRC1 | Monocytes | 2.39298E-35 | 0.635551676 |
| MRC1 | Natural.killer.cell | 2.62247E-48 | 0.715354173 |
| MRC1 | Natural.killer.T.cell | 1.44583E-37 | 0.651096147 |
| MRC1 | Neutrophil | 1.15909E-11 | 0.378643389 |
| MRC1 | NK.cells.resting | 0.000599898 | 0.197004306 |
| MRC1 | Plasma.cells | 0.004391411 | 0.164032509 |
| MRC1 | Plasmacytoid.dendritic.cell | 2.57454E-32 | 0.612768181 |
| MRC1 | Regulatory.T.cell | 1.79552E-74 | 0.820866778 |
| MRC1 | T.follicular.helper.cell | 1.50531E-56 | 0.754959816 |
| C3 | Activated.B.cell | 1.88423E-06 | 0.271096322 |
| C3 | Activated.CD4.T.cell | 0.036898982 | 0.120549604 |
| C3 | Activated.CD8.T.cell | 3.60326E-08 | 0.311432808 |
| C3 | B.cells.naive | 1.38351E-06 | 0.274482658 |
| C3 | Dendritic.cells.activated | 5.79785E-10 | 0.347930712 |
| C3 | Dendritic.cells.resting | 0.038853729 | 0.119336806 |
| C3 | Endothelial.cells | 3.5759E-09 | 0.332439289 |
| C3 | Eosinophil | 0.000111261 | 0.221275843 |
| C3 | Fibroblasts | 6.18182E-12 | 0.383288777 |
| C3 | Gamma.delta.T.cell | 0.118523098 | 0.0903173 |
| C3 | Immature.dendritic.cell | 7.56213E-07 | 0.280976529 |
| C3 | Macrophage | 1.25622E-08 | 0.321214437 |
| C3 | Mast.cell | 1.27323E-07 | 0.299227195 |
| C3 | Mast.cells.resting | 1.26016E-05 | 0.249176014 |
| C3 | MDSC | 1.98976E-14 | 0.422639856 |
| C3 | Monocytes | 3.13722E-10 | 0.35297415 |
| C3 | Natural.killer.cell | 3.46764E-23 | 0.530603744 |
| C3 | Natural.killer.T.cell | 9.49023E-11 | 0.362544894 |
| C3 | Neutrophil | 0.134408119 | 0.086625135 |
| C3 | NK.cells.resting | 0.104390439 | 0.093942456 |
| C3 | Plasma.cells | 0.555081345 | 0.034205905 |
| C3 | Plasmacytoid.dendritic.cell | 6.31454E-10 | 0.347222533 |
| C3 | Regulatory.T.cell | 1.17042E-10 | 0.360889312 |
| C3 | T.follicular.helper.cell | 2.06387E-15 | 0.436861411 |
| JAK1 | Activated.B.cell | 8.61732E-09 | 0.324629933 |
| JAK1 | Activated.CD4.T.cell | 1.61047E-10 | 0.358350981 |
| JAK1 | Activated.CD8.T.cell | 4.76695E-11 | 0.367915557 |
| JAK1 | B.cells.naive | 2.66538E-08 | 0.31426771 |
| JAK1 | Dendritic.cells.activated | 2.10309E-13 | 0.407093127 |
| JAK1 | Dendritic.cells.resting | 6.66107E-09 | 0.32693907 |
| JAK1 | Endothelial.cells | 3.98077E-10 | 0.351029118 |
| JAK1 | Eosinophil | 8.12303E-11 | 0.363766992 |
| JAK1 | Fibroblasts | 0.000374843 | 0.204061203 |
| JAK1 | Gamma.delta.T.cell | 8.98314E-08 | 0.302656019 |
| JAK1 | Immature.dendritic.cell | 1.1425E-09 | 0.342254148 |
| JAK1 | Macrophage | 1.98741E-15 | 0.437092787 |
| JAK1 | Mast.cell | 6.03519E-09 | 0.327818844 |
| JAK1 | Mast.cells.resting | 0.00036124 | 0.204605956 |
| JAK1 | MDSC | 5.35146E-19 | 0.48369741 |
| JAK1 | Monocytes | 4.87577E-15 | 0.431544275 |
| JAK1 | Natural.killer.cell | 4.11537E-14 | 0.417933527 |
| JAK1 | Natural.killer.T.cell | 8.36572E-09 | 0.324896648 |
| JAK1 | Neutrophil | 7.66823E-11 | 0.364218334 |
| JAK1 | NK.cells.resting | 1.46699E-05 | 0.247334152 |
| JAK1 | Plasma.cells | 0.000541049 | 0.198574365 |
| JAK1 | Plasmacytoid.dendritic.cell | 1.2602E-16 | 0.453537463 |
| JAK1 | Regulatory.T.cell | 1.36811E-15 | 0.439371797 |
| JAK1 | T.follicular.helper.cell | 4.76595E-17 | 0.459129554 |
| ABCB1 | Activated.B.cell | 2.44319E-17 | 0.462913805 |
| ABCB1 | Activated.CD4.T.cell | 0.131129305 | 0.087358448 |
| ABCB1 | Activated.CD8.T.cell | 2.41628E-09 | 0.335850116 |
| ABCB1 | B.cells.naive | 2.56966E-26 | 0.561438056 |
| ABCB1 | Dendritic.cells.activated | 1.45638E-09 | 0.340194688 |
| ABCB1 | Dendritic.cells.resting | 2.44208E-09 | 0.335758255 |
| ABCB1 | Endothelial.cells | 7.44613E-08 | 0.304482635 |
| ABCB1 | Eosinophil | 1.52177E-19 | 0.490239056 |
| ABCB1 | Fibroblasts | 2.12037E-12 | 0.391031506 |
| ABCB1 | Gamma.delta.T.cell | 1.29299E-05 | 0.24886535 |
| ABCB1 | Immature.dendritic.cell | 5.13496E-08 | 0.308063482 |
| ABCB1 | Macrophage | 9.16144E-07 | 0.27893199 |
| ABCB1 | Mast.cell | 4.1578E-08 | 0.310076339 |
| ABCB1 | Mast.cells.resting | 3.55846E-10 | 0.351946756 |
| ABCB1 | MDSC | 3.0828E-10 | 0.353116547 |
| ABCB1 | Monocytes | 2.92572E-10 | 0.353541698 |
| ABCB1 | Natural.killer.cell | 2.5527E-10 | 0.354647522 |
| ABCB1 | Natural.killer.T.cell | 0.096239585 | 0.096213115 |
| ABCB1 | Neutrophil | 0.789194154 | 0.015499653 |
| ABCB1 | NK.cells.resting | 1.02198E-05 | 0.251691853 |
| ABCB1 | Plasma.cells | 0.000219795 | 0.211796037 |
| ABCB1 | Plasmacytoid.dendritic.cell | 3.64443E-05 | 0.235993913 |
| ABCB1 | Regulatory.T.cell | 1.13633E-07 | 0.300350323 |
| ABCB1 | T.follicular.helper.cell | 1.74094E-15 | 0.437902888 |
| CREBBP | Activated.B.cell | 0.597946103 | -0.03056743 |
| CREBBP | Activated.CD4.T.cell | 1.85398E-05 | -0.244468535 |
| CREBBP | Activated.CD8.T.cell | 0.011031846 | -0.146563043 |
| CREBBP | B.cells.naive | 0.932605303 | -0.004902941 |
| CREBBP | Dendritic.cells.activated | 0.026798363 | -0.127859474 |
| CREBBP | Dendritic.cells.resting | 0.982981469 | -0.001236719 |
| CREBBP | Endothelial.cells | 0.19920082 | 0.074329792 |
| CREBBP | Eosinophil | 0.61904748 | -0.028820251 |
| CREBBP | Fibroblasts | 0.912363094 | 0.006380795 |
| CREBBP | Gamma.delta.T.cell | 3.86026E-06 | -0.263051734 |
| CREBBP | Immature.dendritic.cell | 0.002294817 | -0.175408778 |
| CREBBP | Macrophage | 0.015572882 | -0.139546642 |
| CREBBP | Mast.cell | 0.229643646 | -0.069562134 |
| CREBBP | Mast.cells.resting | 0.046989906 | -0.114784214 |
| CREBBP | MDSC | 0.1584467 | -0.081629406 |
| CREBBP | Monocytes | 0.761672297 | -0.017582243 |
| CREBBP | Natural.killer.cell | 0.933697219 | -0.00482332 |
| CREBBP | Natural.killer.T.cell | 0.263139525 | -0.064808192 |
| CREBBP | Neutrophil | 0.000108772 | -0.221583976 |
| CREBBP | NK.cells.resting | 0.248392869 | 0.066845831 |
| CREBBP | Plasma.cells | 0.013771251 | -0.142082454 |
| CREBBP | Plasmacytoid.dendritic.cell | 0.701493917 | -0.022220132 |
| CREBBP | Regulatory.T.cell | 0.008774421 | -0.151068687 |
| CREBBP | T.follicular.helper.cell | 0.850635349 | 0.010917209 |
| LRRN3 | Activated.B.cell | 0.018027633 | 0.136476486 |
| LRRN3 | Activated.CD4.T.cell | 0.127578331 | -0.088168765 |
| LRRN3 | Activated.CD8.T.cell | 0.002535151 | 0.173706767 |
| LRRN3 | B.cells.naive | 6.24354E-06 | 0.25750796 |
| LRRN3 | Dendritic.cells.activated | 0.00030583 | 0.207042746 |
| LRRN3 | Dendritic.cells.resting | 0.711486865 | -0.021440926 |
| LRRN3 | Endothelial.cells | 7.19693E-05 | 0.227135204 |
| LRRN3 | Eosinophil | 1.54827E-05 | 0.246677218 |
| LRRN3 | Fibroblasts | 1.2586E-08 | 0.321197191 |
| LRRN3 | Gamma.delta.T.cell | 0.286343684 | -0.061754788 |
| LRRN3 | Immature.dendritic.cell | 0.102886257 | 0.094350649 |
| LRRN3 | Macrophage | 0.078904066 | 0.101606579 |
| LRRN3 | Mast.cell | 4.43882E-05 | 0.233461904 |
| LRRN3 | Mast.cells.resting | 6.78346E-07 | 0.282127353 |
| LRRN3 | MDSC | 0.013341098 | 0.142730809 |
| LRRN3 | Monocytes | 0.014961223 | 0.140377296 |
| LRRN3 | Natural.killer.cell | 5.85512E-06 | 0.258255868 |
| LRRN3 | Natural.killer.T.cell | 0.608736971 | 0.0296706 |
| LRRN3 | Neutrophil | 0.19230278 | -0.075482444 |
| LRRN3 | NK.cells.resting | 0.052814662 | 0.111915774 |
| LRRN3 | Plasma.cells | 0.022597405 | -0.131622115 |
| LRRN3 | Plasmacytoid.dendritic.cell | 0.002439257 | 0.174367487 |
| LRRN3 | Regulatory.T.cell | 0.013186148 | 0.142968869 |
| LRRN3 | T.follicular.helper.cell | 1.47551E-05 | 0.247263745 |
| GTF3C1 | Activated.B.cell | 0.000708396 | -0.194450981 |
| GTF3C1 | Activated.CD4.T.cell | 2.62059E-05 | -0.240167695 |
| GTF3C1 | Activated.CD8.T.cell | 2.17646E-05 | -0.242485322 |
| GTF3C1 | B.cells.naive | 0.050944441 | -0.112806708 |
| GTF3C1 | Dendritic.cells.activated | 7.05218E-07 | -0.281716505 |
| GTF3C1 | Dendritic.cells.resting | 3.10664E-06 | -0.265515514 |
| GTF3C1 | Endothelial.cells | 0.853057561 | -0.010738122 |
| GTF3C1 | Eosinophil | 0.000182724 | -0.214408969 |
| GTF3C1 | Fibroblasts | 0.063018183 | -0.107470658 |
| GTF3C1 | Gamma.delta.T.cell | 9.49277E-06 | -0.252571845 |
| GTF3C1 | Immature.dendritic.cell | 0.002175622 | -0.176314074 |
| GTF3C1 | Macrophage | 1.44301E-07 | -0.297985691 |
| GTF3C1 | Mast.cell | 0.003942501 | -0.165971305 |
| GTF3C1 | Mast.cells.resting | 0.168678251 | -0.079676372 |
| GTF3C1 | MDSC | 4.89823E-05 | -0.232186755 |
| GTF3C1 | Monocytes | 0.104796325 | -0.093833106 |
| GTF3C1 | Natural.killer.cell | 0.006778425 | -0.156012784 |
| GTF3C1 | Natural.killer.T.cell | 0.31235059 | -0.058524127 |
| GTF3C1 | Neutrophil | 8.0924E-05 | -0.225572911 |
| GTF3C1 | NK.cells.resting | 5.76603E-05 | -0.230059093 |
| GTF3C1 | Plasma.cells | 3.19023E-05 | -0.237687519 |
| GTF3C1 | Plasmacytoid.dendritic.cell | 0.767131696 | -0.017167414 |
| GTF3C1 | Regulatory.T.cell | 2.32863E-06 | -0.268748096 |
| GTF3C1 | T.follicular.helper.cell | 0.001721003 | -0.180243073 |
| CARD11 | Activated.B.cell | 0.020096174 | 0.134160609 |
| CARD11 | Activated.CD4.T.cell | 0.500957754 | -0.03900276 |
| CARD11 | Activated.CD8.T.cell | 0.257927179 | -0.065519182 |
| CARD11 | B.cells.naive | 0.007951279 | 0.152972075 |
| CARD11 | Dendritic.cells.activated | 0.517640725 | -0.037497179 |
| CARD11 | Dendritic.cells.resting | 0.174725283 | 0.078563411 |
| CARD11 | Endothelial.cells | 0.720556993 | 0.020737005 |
| CARD11 | Eosinophil | 0.991121922 | -0.000645125 |
| CARD11 | Fibroblasts | 0.013432142 | -0.142592063 |
| CARD11 | Gamma.delta.T.cell | 0.45966346 | 0.042848613 |
| CARD11 | Immature.dendritic.cell | 0.293539499 | 0.060842009 |
| CARD11 | Macrophage | 0.790278133 | -0.015418052 |
| CARD11 | Mast.cell | 0.643625056 | -0.026817471 |
| CARD11 | Mast.cells.resting | 0.087886247 | 0.09870507 |
| CARD11 | MDSC | 0.550646785 | -0.034589846 |
| CARD11 | Monocytes | 0.100279555 | 0.095069362 |
| CARD11 | Natural.killer.cell | 0.260508728 | -0.065165822 |
| CARD11 | Natural.killer.T.cell | 0.790160022 | -0.015426942 |
| CARD11 | Neutrophil | 0.676827049 | 0.024161126 |
| CARD11 | NK.cells.resting | 0.252416101 | 0.066281789 |
| CARD11 | Plasma.cells | 0.0005614 | 0.19801425 |
| CARD11 | Plasmacytoid.dendritic.cell | 0.610892041 | 0.029492343 |
| CARD11 | Regulatory.T.cell | 0.445875069 | -0.044174909 |
| CARD11 | T.follicular.helper.cell | 0.953744653 | -0.00336295 |
| FN1 | Activated.B.cell | 0.112332638 | -0.091860971 |
| FN1 | Activated.CD4.T.cell | 0.131550971 | 0.087263355 |
| FN1 | Activated.CD8.T.cell | 0.107641843 | 0.093075743 |
| FN1 | B.cells.naive | 0.44921906 | 0.043851156 |
| FN1 | Dendritic.cells.activated | 8.35103E-06 | 0.254092285 |
| FN1 | Dendritic.cells.resting | 0.151442245 | -0.083021895 |
| FN1 | Endothelial.cells | 5.71397E-10 | 0.348051428 |
| FN1 | Eosinophil | 0.947365419 | -0.003827376 |
| FN1 | Fibroblasts | 2.3453E-32 | 0.61308521 |
| FN1 | Gamma.delta.T.cell | 0.06791267 | 0.105546287 |
| FN1 | Immature.dendritic.cell | 1.38669E-09 | 0.340611923 |
| FN1 | Macrophage | 4.82322E-09 | 0.329807201 |
| FN1 | Mast.cell | 6.24348E-13 | 0.399637565 |
| FN1 | Mast.cells.resting | 2.10519E-06 | 0.269869443 |
| FN1 | MDSC | 1.28666E-06 | 0.275271765 |
| FN1 | Monocytes | 5.55589E-05 | 0.230545114 |
| FN1 | Natural.killer.cell | 2.95739E-16 | 0.448545697 |
| FN1 | Natural.killer.T.cell | 2.30209E-21 | 0.511081307 |
| FN1 | Neutrophil | 0.44757564 | 0.044010095 |
| FN1 | NK.cells.resting | 0.002038001 | -0.177417384 |
| FN1 | Plasma.cells | 6.71381E-07 | -0.282236373 |
| FN1 | Plasmacytoid.dendritic.cell | 4.19759E-19 | 0.484971761 |
| FN1 | Regulatory.T.cell | 9.39143E-17 | 0.455239445 |
| FN1 | T.follicular.helper.cell | 1.38065E-12 | 0.394079918 |
| ATM | Activated.B.cell | 2.62636E-20 | 0.499149834 |
| ATM | Activated.CD4.T.cell | 0.542656016 | 0.035285529 |
| ATM | Activated.CD8.T.cell | 6.62078E-07 | 0.282383713 |
| ATM | B.cells.naive | 1.61712E-17 | 0.465227666 |
| ATM | Dendritic.cells.activated | 3.98275E-14 | 0.418147243 |
| ATM | Dendritic.cells.resting | 0.00015001 | 0.217165419 |
| ATM | Endothelial.cells | 3.41869E-20 | 0.497829051 |
| ATM | Eosinophil | 9.83572E-13 | 0.396467531 |
| ATM | Fibroblasts | 4.32892E-14 | 0.417603153 |
| ATM | Gamma.delta.T.cell | 0.076494131 | 0.102430308 |
| ATM | Immature.dendritic.cell | 1.84414E-14 | 0.423127817 |
| ATM | Macrophage | 1.02594E-07 | 0.301355327 |
| ATM | Mast.cell | 7.45681E-11 | 0.364437095 |
| ATM | Mast.cells.resting | 8.74732E-21 | 0.504596464 |
| ATM | MDSC | 5.13718E-12 | 0.384642855 |
| ATM | Monocytes | 2.9195E-14 | 0.420166047 |
| ATM | Natural.killer.cell | 1.24704E-11 | 0.378098052 |
| ATM | Natural.killer.T.cell | 4.6557E-05 | 0.232845134 |
| ATM | Neutrophil | 0.141344106 | 0.085118077 |
| ATM | NK.cells.resting | 0.006881306 | 0.155727982 |
| ATM | Plasma.cells | 4.17316E-05 | 0.234257365 |
| ATM | Plasmacytoid.dendritic.cell | 3.16574E-16 | 0.448143761 |
| ATM | Regulatory.T.cell | 1.72033E-07 | 0.296232375 |
| ATM | T.follicular.helper.cell | 9.33489E-18 | 0.468281687 |
| LRP1 | Activated.B.cell | 0.351078576 | -0.054024075 |
| LRP1 | Activated.CD4.T.cell | 2.42326E-12 | -0.39007633 |
| LRP1 | Activated.CD8.T.cell | 0.000153549 | -0.216841357 |
| LRP1 | B.cells.naive | 0.761989675 | 0.017558103 |
| LRP1 | Dendritic.cells.activated | 0.391856848 | 0.049612561 |
| LRP1 | Dendritic.cells.resting | 0.271048954 | 0.063747538 |
| LRP1 | Endothelial.cells | 1.90394E-05 | 0.244140813 |
| LRP1 | Eosinophil | 0.149831108 | 0.083349062 |
| LRP1 | Fibroblasts | 0.002514719 | 0.173845614 |
| LRP1 | Gamma.delta.T.cell | 0.134259081 | -0.086658167 |
| LRP1 | Immature.dendritic.cell | 0.405954273 | 0.04815297 |
| LRP1 | Macrophage | 0.522427258 | 0.037069865 |
| LRP1 | Mast.cell | 0.027209691 | 0.127518969 |
| LRP1 | Mast.cells.resting | 0.00732044 | 0.154553585 |
| LRP1 | MDSC | 0.218477814 | -0.07125503 |
| LRP1 | Monocytes | 0.797195054 | 0.014898055 |
| LRP1 | Natural.killer.cell | 0.344945631 | 0.05471474 |
| LRP1 | Natural.killer.T.cell | 0.782484971 | 0.016005407 |
| LRP1 | Neutrophil | 0.353417893 | -0.053762639 |
| LRP1 | NK.cells.resting | 0.051198718 | 0.112683992 |
| LRP1 | Plasma.cells | 0.005146311 | -0.161142793 |
| LRP1 | Plasmacytoid.dendritic.cell | 0.000146217 | 0.217520662 |
| LRP1 | Regulatory.T.cell | 0.254176388 | -0.066036964 |
| LRP1 | T.follicular.helper.cell | 0.007306529 | 0.15458981 |
| NFATC2 | Activated.B.cell | 2.55285E-05 | 0.240495944 |
| NFATC2 | Activated.CD4.T.cell | 0.059802897 | -0.108802801 |
| NFATC2 | Activated.CD8.T.cell | 0.877626563 | 0.008927052 |
| NFATC2 | B.cells.naive | 2.94588E-06 | 0.266114538 |
| NFATC2 | Dendritic.cells.activated | 0.157286977 | 0.081856705 |
| NFATC2 | Dendritic.cells.resting | 0.050076957 | 0.11322924 |
| NFATC2 | Endothelial.cells | 7.3463E-06 | 0.255603705 |
| NFATC2 | Eosinophil | 0.01129884 | 0.146085569 |
| NFATC2 | Fibroblasts | 0.165847326 | 0.080207659 |
| NFATC2 | Gamma.delta.T.cell | 0.553179341 | -0.034370395 |
| NFATC2 | Immature.dendritic.cell | 0.303773377 | 0.059569211 |
| NFATC2 | Macrophage | 0.655078022 | -0.025895107 |
| NFATC2 | Mast.cell | 0.532612434 | 0.036167186 |
| NFATC2 | Mast.cells.resting | 0.002339981 | 0.175076918 |
| NFATC2 | MDSC | 0.716950072 | 0.021016563 |
| NFATC2 | Monocytes | 0.002014836 | 0.177609754 |
| NFATC2 | Natural.killer.cell | 0.020205645 | 0.13404392 |
| NFATC2 | Natural.killer.T.cell | 0.721521983 | -0.020662293 |
| NFATC2 | Neutrophil | 0.064585321 | -0.106841546 |
| NFATC2 | NK.cells.resting | 0.0037786 | 0.1667292 |
| NFATC2 | Plasma.cells | 0.01044616 | 0.147647308 |
| NFATC2 | Plasmacytoid.dendritic.cell | 0.020505531 | 0.13372705 |
| NFATC2 | Regulatory.T.cell | 0.433800452 | -0.04535557 |
| NFATC2 | T.follicular.helper.cell | 0.004337899 | 0.16425397 |
