# Supplementary material for: Immunogenomic characterization in gastric cancer identifies microenvironmental and immunotherapeutically relevant gene signatures
Source: Immun Inflamm Dis. 2021 Sep 28;10(1):43–59. doi: 10.1002/iid3.539 (PMC8669697; doi:10.1002/iid3.539)
Supplement: Supplementary file 10 — Supplementary information. [file IID3-10-43-s004.docx]

**Table-S9.** Survival analyses for immunogenomic phenotype-related differentially expressed genes.

| **id** | **HR** | **HR.95L** | **HR.95H** | **pvalue** |
| --- | --- | --- | --- | --- |
| FERMT2 | 1.54930214 | 1.352531639 | 1.774699424 | 2.66E-10 |
| ACTA2 | 1.902874434 | 1.547788137 | 2.339422964 | 1.03E-09 |
| TAGLN | 1.4285194 | 1.272852256 | 1.603224308 | 1.38E-09 |
| CRYAB | 1.382785283 | 1.244985431 | 1.535837361 | 1.44E-09 |
| CFL2 | 1.427118139 | 1.271300698 | 1.602033402 | 1.65E-09 |
| SLIT2 | 1.385918236 | 1.244222803 | 1.543750325 | 3.01E-09 |
| COX7A1 | 1.539612178 | 1.334733563 | 1.775939202 | 3.16E-09 |
| KCNMB1 | 1.887404378 | 1.527722385 | 2.331768729 | 3.90E-09 |
| CRY2 | 3.991492531 | 2.494960269 | 6.385677889 | 7.77E-09 |
| MYLK | 1.668034422 | 1.401108526 | 1.985812506 | 8.89E-09 |
| TCEAL7 | 1.376004324 | 1.232698535 | 1.535969944 | 1.28E-08 |
| FILIP1 | 1.416358578 | 1.256158804 | 1.596988864 | 1.32E-08 |
| MPDZ | 1.515372778 | 1.311380778 | 1.75109678 | 1.75E-08 |
| LHFP | 1.584786759 | 1.349991439 | 1.860418518 | 1.82E-08 |
| PBX3 | 1.619846985 | 1.365714512 | 1.921268486 | 3.03E-08 |
| MSRB3 | 1.506306752 | 1.302962193 | 1.74138593 | 3.08E-08 |
| S1PR3 | 2.085191355 | 1.606808162 | 2.706000062 | 3.26E-08 |
| MGP | 1.358061866 | 1.217974391 | 1.514261749 | 3.59E-08 |
| EFEMP2 | 1.643150927 | 1.375569986 | 1.962782698 | 4.35E-08 |
| LRCH2 | 1.504082461 | 1.299422041 | 1.740977124 | 4.51E-08 |
| BOC | 1.81566759 | 1.466117032 | 2.248557739 | 4.58E-08 |
| CNN1 | 1.222841376 | 1.13747146 | 1.314618506 | 5.08E-08 |
| SFRP1 | 1.823051583 | 1.466862112 | 2.265732441 | 6.15E-08 |
| LAMA2 | 1.894217002 | 1.500217081 | 2.391692574 | 7.92E-08 |
| DNAJB5 | 1.736785347 | 1.419096654 | 2.125594007 | 8.51E-08 |
| CALD1 | 1.822890707 | 1.462800053 | 2.271623196 | 8.92E-08 |
| FXYD6 | 1.494824987 | 1.289786546 | 1.73245856 | 9.26E-08 |
| AOC3 | 1.339908397 | 1.203024821 | 1.492366974 | 1.03E-07 |
| LOC728392 | 1.529113749 | 1.306741449 | 1.789327843 | 1.18E-07 |
| CC2D2A | 3.258575073 | 2.103274917 | 5.04846581 | 1.23E-07 |
| TMEM47 | 1.430246458 | 1.252366458 | 1.633391662 | 1.29E-07 |
| ASB2 | 1.594288657 | 1.34066172 | 1.895896841 | 1.32E-07 |
| PRELP | 1.748139945 | 1.41799739 | 2.155147314 | 1.69E-07 |
| LAYN | 2.591255842 | 1.812002643 | 3.705627507 | 1.82E-07 |
| LINC01140 | 1.943961089 | 1.511597989 | 2.499993214 | 2.23E-07 |
| PKD2 | 1.556158943 | 1.315799398 | 1.840425417 | 2.39E-07 |
| IGFBP7 | 1.88414661 | 1.481554743 | 2.39613721 | 2.40E-07 |
| DPYSL3 | 1.56490655 | 1.319623445 | 1.855781298 | 2.63E-07 |
| SUPT20H | 2.821815353 | 1.895061832 | 4.201784739 | 3.27E-07 |
| COL14A1 | 1.520801163 | 1.293346063 | 1.788257794 | 3.94E-07 |
| LTBP1 | 1.523909513 | 1.294059583 | 1.794585221 | 4.41E-07 |
| ADGRA2 | 1.659936202 | 1.363003472 | 2.021556256 | 4.66E-07 |
| CAV1 | 1.502504214 | 1.281786658 | 1.761228281 | 5.10E-07 |
| AP1S2 | 1.673785962 | 1.368822886 | 2.046692434 | 5.19E-07 |
| BEX4 | 1.393678863 | 1.222338632 | 1.589036558 | 7.06E-07 |
| MIR100HG | 1.518274311 | 1.287226231 | 1.790793901 | 7.14E-07 |
| SNURF | 1.689246966 | 1.372424479 | 2.07920753 | 7.52E-07 |
| FLNA | 1.479456432 | 1.266345554 | 1.728431332 | 7.99E-07 |
| ZEB1 | 1.518898337 | 1.286469018 | 1.793321197 | 8.11E-07 |
| RASGRP2 | 2.924142748 | 1.906435354 | 4.485130215 | 8.82E-07 |
| KCNMA1 | 1.496150266 | 1.273138837 | 1.758225853 | 9.97E-07 |
| KCNH2 | 1.778669014 | 1.412141459 | 2.240330415 | 1.00E-06 |
| KCNJ8 | 1.471484336 | 1.260357668 | 1.717977528 | 1.02E-06 |
| STON1 | 1.669203808 | 1.358823401 | 2.050480842 | 1.05E-06 |
| SPARCL1 | 1.449819544 | 1.247633669 | 1.684770748 | 1.25E-06 |
| NDN | 1.398410873 | 1.219968971 | 1.60295304 | 1.47E-06 |
| FBXL7 | 1.506011496 | 1.274396922 | 1.779720735 | 1.54E-06 |
| DDR2 | 1.583346381 | 1.312741008 | 1.909733715 | 1.54E-06 |
| SYNC | 1.273732578 | 1.153739091 | 1.406205869 | 1.64E-06 |
| REEP1 | 1.278044228 | 1.155531779 | 1.413545761 | 1.83E-06 |
| OMD | 1.273387223 | 1.152826301 | 1.406556232 | 1.91E-06 |
| THBS4 | 1.233057173 | 1.130381959 | 1.345058614 | 2.33E-06 |
| FSTL1 | 1.572571363 | 1.302061443 | 1.899281102 | 2.59E-06 |
| ADAMTS1 | 1.453945008 | 1.243482476 | 1.700028852 | 2.71E-06 |
| PLCL1 | 1.718038559 | 1.370167173 | 2.154230919 | 2.76E-06 |
| PPP3R1 | 0.445179898 | 0.316650655 | 0.625879462 | 3.23E-06 |
| PDE1A | 1.65967447 | 1.339761729 | 2.055977034 | 3.53E-06 |
| ATP2B4 | 1.926140419 | 1.459849078 | 2.541370179 | 3.57E-06 |
| CLDND1 | 3.262433371 | 1.97718911 | 5.383132776 | 3.70E-06 |
| HSPB2 | 1.864959605 | 1.430922164 | 2.430652355 | 4.01E-06 |
| AKT3 | 1.937000988 | 1.454574822 | 2.57942924 | 6.07E-06 |
| SYNE1 | 2.350084192 | 1.62198688 | 3.405018733 | 6.29E-06 |
| ZFPM2 | 1.26291401 | 1.140794963 | 1.398105573 | 6.84E-06 |
| MMP12 | 0.840452094 | 0.779145617 | 0.906582423 | 6.87E-06 |
| CSRP2 | 1.492547024 | 1.253376439 | 1.777356387 | 6.97E-06 |
| FBLN5 | 1.404283555 | 1.210831171 | 1.628643489 | 7.13E-06 |
| ZNF382 | 3.007204205 | 1.852713335 | 4.881098958 | 8.38E-06 |
| MRGPRF | 1.280196447 | 1.14776575 | 1.427907169 | 9.27E-06 |
| KCNE4 | 1.817699045 | 1.395664819 | 2.367351942 | 9.29E-06 |
| EFEMP1 | 1.417195068 | 1.212983838 | 1.655786169 | 1.12E-05 |
| EMCN | 1.453097915 | 1.228562751 | 1.718669679 | 1.28E-05 |
| EPT1 | 0.450745827 | 0.315037478 | 0.644913114 | 1.30E-05 |
| ATP8B2 | 1.803180107 | 1.381454883 | 2.353647982 | 1.44E-05 |
| PART1 | 1.746422864 | 1.357233288 | 2.24721339 | 1.46E-05 |
| COL4A5 | 1.610303614 | 1.29698687 | 1.99930916 | 1.59E-05 |
| A2M | 2.362290935 | 1.59765774 | 3.492874801 | 1.65E-05 |
| PPP1R1A | 1.479048172 | 1.237084561 | 1.768337886 | 1.75E-05 |
| ITGBL1 | 1.302885963 | 1.15343575 | 1.47170038 | 2.08E-05 |
| PREX2 | 4.172163729 | 2.161181589 | 8.054367235 | 2.08E-05 |
| ZNF25 | 1.682133877 | 1.323836264 | 2.1374051 | 2.09E-05 |
| EFS | 1.631404893 | 1.301476647 | 2.044970941 | 2.18E-05 |
| EPHA3 | 1.906473504 | 1.413063227 | 2.572171683 | 2.41E-05 |
| CASP1 | 0.734222857 | 0.636027215 | 0.847578832 | 2.47E-05 |
| ZDHHC12 | 0.418378791 | 0.27849746 | 0.628518526 | 2.71E-05 |
| LIX1L | 2.183766263 | 1.513403053 | 3.151067444 | 2.98E-05 |
| ANKRD35 | 1.551668525 | 1.261328043 | 1.908841419 | 3.23E-05 |
| MAGI2-AS3 | 1.625648665 | 1.292292257 | 2.044996841 | 3.33E-05 |
| C11orf96 | 1.796641597 | 1.360442774 | 2.372698866 | 3.64E-05 |
| OLFML1 | 1.440462846 | 1.211138014 | 1.713209548 | 3.70E-05 |
| LOC101929787 | 1.427024849 | 1.204389339 | 1.6908153 | 3.98E-05 |
| EBF1 | 1.527679973 | 1.248030694 | 1.869990948 | 3.99E-05 |
| HTR2A | 1.54694376 | 1.255449053 | 1.906118764 | 4.21E-05 |
| FILIP1L | 1.748496746 | 1.33787959 | 2.285139032 | 4.29E-05 |
| FAM129A | 1.304400993 | 1.1481989 | 1.481852971 | 4.44E-05 |
| MEF2C | 1.678090641 | 1.308539768 | 2.152008114 | 4.53E-05 |
| GPR155 | 1.341318537 | 1.164072403 | 1.545552847 | 4.89E-05 |
| CNRIP1 | 1.405064659 | 1.191931917 | 1.656308275 | 5.08E-05 |
| COL8A2 | 1.476247363 | 1.222726895 | 1.782332822 | 5.09E-05 |
| CKB | 1.312242815 | 1.150092478 | 1.49725456 | 5.39E-05 |
| CCDC80 | 1.256762133 | 1.124588695 | 1.404469978 | 5.55E-05 |
| ECM2 | 1.570104864 | 1.260056729 | 1.956443092 | 5.83E-05 |
| TSHZ3 | 1.427176728 | 1.199832708 | 1.69759784 | 5.87E-05 |
| GYPC | 1.582101553 | 1.264451522 | 1.979550248 | 6.02E-05 |
| SASH1 | 1.954658429 | 1.408445772 | 2.712699096 | 6.12E-05 |
| LTC4S | 2.50011896 | 1.594223178 | 3.920777783 | 6.57E-05 |
| LOC101930349 | 1.543592522 | 1.246156736 | 1.912021021 | 7.04E-05 |
| CYFIP2 | 1.430669097 | 1.197338301 | 1.709470134 | 8.06E-05 |
| THSD7A | 1.44929797 | 1.205005998 | 1.743115478 | 8.15E-05 |
| MEIS1 | 1.348305617 | 1.160703829 | 1.566229033 | 9.25E-05 |
| LOC100287852 | 0.448431634 | 0.299716059 | 0.670938125 | 9.57E-05 |
| DNM3OS | 1.372969605 | 1.170165408 | 1.610922289 | 0.000101494 |
| OLFML3 | 1.358222186 | 1.162245402 | 1.5872444 | 0.00011757 |
| JAM2 | 1.29321499 | 1.132933297 | 1.476172528 | 0.000139715 |
| ITPR1 | 1.818946168 | 1.329349355 | 2.488860547 | 0.000184442 |
| CXCL3 | 0.810396499 | 0.725712505 | 0.904962338 | 0.000188965 |
| CCDC69 | 1.324355515 | 1.141380974 | 1.536662665 | 0.000212971 |
| UBE2QL1 | 1.408578691 | 1.174338975 | 1.68954107 | 0.000222683 |
| BNC2 | 1.413312269 | 1.17579629 | 1.698807513 | 0.000228618 |
| PRMT3 | 0.596851497 | 0.451911776 | 0.788277111 | 0.000276733 |
| F13A1 | 1.241224057 | 1.104631904 | 1.394706374 | 0.000280258 |
| CTSS | 0.713925082 | 0.594101906 | 0.857915144 | 0.000324635 |
| CXCL12 | 1.26325761 | 1.111749562 | 1.435413014 | 0.000336917 |
| CRISPLD2 | 1.823448093 | 1.312182331 | 2.533918396 | 0.000345786 |
| CYP1B1 | 1.246765651 | 1.104640448 | 1.40717696 | 0.000354868 |
| TCF4 | 1.426650664 | 1.173706837 | 1.734106042 | 0.000359062 |
| NEXN | 1.2141756 | 1.090352851 | 1.352059919 | 0.000406022 |
| SERPINF1 | 1.292517534 | 1.120837089 | 1.490494552 | 0.000417447 |
| CARD16 | 0.639444863 | 0.49818468 | 0.820759346 | 0.000446706 |
| FAM91A1 | 0.29407499 | 0.148215261 | 0.58347635 | 0.000463356 |
| GUCY1A3 | 1.261880881 | 1.107603638 | 1.437647281 | 0.000472291 |
| FABP4 | 1.34010917 | 1.136377211 | 1.580366599 | 0.000502442 |
| TIMP3 | 1.348280125 | 1.137719274 | 1.597810054 | 0.000562163 |
| NHSL2 | 1.803926282 | 1.290049351 | 2.522500422 | 0.000563208 |
| DAPP1 | 0.574950239 | 0.419723022 | 0.787585526 | 0.000566491 |
| DCN | 1.438526131 | 1.169600562 | 1.769285598 | 0.000573984 |
| GBP4 | 0.739651014 | 0.622938332 | 0.878230789 | 0.000577657 |
| CILP | 1.250467402 | 1.100987162 | 1.420242468 | 0.000579373 |
| LTBP2 | 1.340446128 | 1.13404984 | 1.584406399 | 0.000593638 |
| LINC01279 | 1.153510936 | 1.063155919 | 1.251545006 | 0.000600235 |
| FNBP1 | 1.54316643 | 1.204323726 | 1.977344279 | 0.000604082 |
| PALLD | 1.462555923 | 1.173266755 | 1.823174329 | 0.000722234 |
| PMAIP1 | 0.770226316 | 0.660650858 | 0.897975944 | 0.000854912 |
| SLMAP | 1.521597686 | 1.188341324 | 1.948311879 | 0.00087438 |
| DTYMK | 0.408656709 | 0.241134945 | 0.692559537 | 0.000884592 |
| EMILIN1 | 1.394390533 | 1.145076218 | 1.697987371 | 0.000940088 |
| AURKA | 0.695754209 | 0.561141313 | 0.862659563 | 0.000944404 |
| PRKAR2B | 1.254299143 | 1.096345682 | 1.435009382 | 0.00096887 |
| FMO2 | 1.277486288 | 1.104518575 | 1.477540761 | 0.00096959 |
| SKA3 | 0.691195932 | 0.554293722 | 0.861910928 | 0.001039948 |
| HLA-DRB6 | 0.56218951 | 0.398434708 | 0.793246768 | 0.001043573 |
| USP30-AS1 | 0.576332631 | 0.414418943 | 0.801506077 | 0.001057095 |
| CLUH | 0.316488432 | 0.158616264 | 0.631492164 | 0.001097897 |
| RTEL1-TNFRSF6B | 0.596192529 | 0.436606264 | 0.814110013 | 0.001138536 |
| OVOL1 | 0.687690524 | 0.548568858 | 0.862094612 | 0.001167448 |
| FAM13B | 1.537752328 | 1.185673371 | 1.994379126 | 0.001179597 |
| PPIF | 0.681700205 | 0.540659071 | 0.859534585 | 0.001196117 |
| ECHDC2 | 1.730917007 | 1.241485147 | 2.413298051 | 0.001213772 |
| MOXD1 | 1.40376616 | 1.142702909 | 1.724472228 | 0.00123524 |
| NUDT15 | 0.62417912 | 0.468533752 | 0.831529366 | 0.001279165 |
| IGF1 | 1.356197973 | 1.125929612 | 1.633559435 | 0.001330642 |
| ATRNL1 | 2.529581145 | 1.432075451 | 4.468186898 | 0.001387922 |
| HCFC2 | 1.647179656 | 1.211391172 | 2.239739632 | 0.001457114 |
| MIR3658 | 0.567181013 | 0.399436777 | 0.805369761 | 0.001524761 |
| CD36 | 1.429813104 | 1.144391765 | 1.786421026 | 0.001648817 |
| HAUS6 | 0.697371664 | 0.55657767 | 0.87378144 | 0.001732525 |
| APITD1-CORT | 0.611885373 | 0.448973106 | 0.833911219 | 0.001871803 |
| CREBRF | 1.729732091 | 1.224152717 | 2.444117523 | 0.001892738 |
| SLC52A2 | 0.637826379 | 0.479969065 | 0.84760148 | 0.00193735 |
| ADRB2 | 1.304168646 | 1.102196688 | 1.543150943 | 0.001978693 |
| TTLL4 | 0.327554587 | 0.161181106 | 0.665661194 | 0.002036845 |
| RABL6 | 0.371985031 | 0.197348298 | 0.701160662 | 0.002230589 |
| FGF7P3 | 1.260896967 | 1.086544219 | 1.463227297 | 0.002264923 |
| ACAT2 | 0.696979572 | 0.552718758 | 0.878892777 | 0.00228088 |
| GAS7 | 1.660387051 | 1.198724514 | 2.299848821 | 0.002285291 |
| NLRC5 | 0.720924271 | 0.584122156 | 0.889765607 | 0.002304812 |
| MFAP5 | 1.171813206 | 1.058074306 | 1.297778598 | 0.002337661 |
| SLC25A39 | 0.594619561 | 0.425250279 | 0.83144548 | 0.002372506 |
| PSMA4 | 0.520179922 | 0.341147553 | 0.793167499 | 0.002393093 |
| PDGFC | 1.315135018 | 1.101602319 | 1.570058529 | 0.002442096 |
| UBA6 | 0.437711881 | 0.256230819 | 0.74773086 | 0.002494364 |
| TNFSF10 | 0.773705017 | 0.654676147 | 0.914374925 | 0.00261058 |
| KLRD1 | 0.721947427 | 0.583188603 | 0.89372132 | 0.002773922 |
| ACKR1 | 1.214027313 | 1.068046462 | 1.379960863 | 0.003006177 |
| GBP1P1 | 0.44315911 | 0.258279708 | 0.760377182 | 0.003132174 |
| TSC22D3 | 1.820045547 | 1.222763482 | 2.70908139 | 0.003167664 |
| EVL | 1.526653204 | 1.151868142 | 2.023382643 | 0.003243204 |
| SLC24A3 | 1.244830244 | 1.075795479 | 1.440424661 | 0.003269775 |
| RNLS | 1.692564446 | 1.19180992 | 2.403717536 | 0.003277534 |
| LDHA | 0.483000802 | 0.296973739 | 0.785556918 | 0.003361454 |
| MCC | 1.427415601 | 1.125195167 | 1.810810566 | 0.003370861 |
| CYYR1 | 1.328331534 | 1.098136902 | 1.60678023 | 0.003454617 |
| WBSCR22 | 0.389635865 | 0.206314981 | 0.735846261 | 0.003666525 |
| GBP1 | 0.750215594 | 0.617947452 | 0.910794982 | 0.003682373 |
| HACD4 | 1.673395462 | 1.182039491 | 2.369000692 | 0.003696916 |
| CXCL1 | 0.867880399 | 0.788638382 | 0.955084618 | 0.003723417 |
| DPT | 1.224630054 | 1.067520592 | 1.404861677 | 0.003819733 |
| RSPO3 | 1.141778521 | 1.042451989 | 1.250569047 | 0.004299498 |
| PJA2 | 1.539223652 | 1.14286268 | 2.073048227 | 0.0045255 |
| PHLDB2 | 1.705530934 | 1.179447021 | 2.466270814 | 0.004553738 |
| CENPF | 0.700764405 | 0.547424001 | 0.897057401 | 0.004769905 |
| CEP85L | 1.66257259 | 1.166997459 | 2.368597801 | 0.004875115 |
| TIAM1 | 1.589277972 | 1.150503378 | 2.195390749 | 0.00494671 |
| SHE | 1.600621862 | 1.151960652 | 2.224025917 | 0.005064401 |
| LOC101928361 | 1.513055077 | 1.131543625 | 2.023196999 | 0.005212138 |
| RECQL4 | 0.590783521 | 0.408328005 | 0.85476667 | 0.005228078 |
| ZNF521 | 1.224185662 | 1.061937159 | 1.41122337 | 0.005297417 |
| GMCL1 | 0.588405681 | 0.404937654 | 0.854998891 | 0.005408875 |
| LAG3 | 0.710715818 | 0.558542636 | 0.904348105 | 0.005472425 |
| ASPN | 1.153024423 | 1.040999808 | 1.27710429 | 0.006323746 |
| HOPX | 1.667761622 | 1.155241539 | 2.407659984 | 0.006327944 |
| BIRC3 | 0.79647815 | 0.676130693 | 0.938246775 | 0.006476727 |
| KLRC2 | 0.846490411 | 0.750666467 | 0.954546456 | 0.006550127 |
| ATAD3B | 0.390533145 | 0.198167204 | 0.769633594 | 0.006598704 |
| CDCP1 | 0.638193875 | 0.461447389 | 0.882638913 | 0.006637364 |
| ISCU | 1.914689632 | 1.197696457 | 3.060906095 | 0.006655423 |
| RRP9 | 0.539288773 | 0.34472499 | 0.84366492 | 0.006840238 |
| TMEM177 | 0.527827883 | 0.331449086 | 0.840558279 | 0.007111163 |
| GSS | 0.614059331 | 0.430442541 | 0.876002779 | 0.007138798 |
| ABCA6 | 1.285976678 | 1.070279812 | 1.545143613 | 0.007252324 |
| TUBB4B | 0.623068542 | 0.4406604 | 0.880983195 | 0.007428986 |
| POLR3A | 0.411590683 | 0.214399494 | 0.790145944 | 0.007634869 |
| TRDV3 | 0.68198233 | 0.513871519 | 0.905089855 | 0.00803669 |
| TRIM69 | 0.521784864 | 0.322409777 | 0.844451574 | 0.008090766 |
| MIR7112 | 0.607052981 | 0.419411043 | 0.878644775 | 0.008151647 |
| CCL19 | 1.134020087 | 1.032272413 | 1.245796692 | 0.008736639 |
| TMEM255A | 1.184205802 | 1.043489802 | 1.343897543 | 0.008804942 |
| STK17B | 0.615017196 | 0.427348996 | 0.885098959 | 0.008868381 |
| DSCC1 | 0.597083857 | 0.405652081 | 0.878854438 | 0.008930179 |
| PAIP2B | 1.27661929 | 1.062893008 | 1.533321604 | 0.0089899 |
| CD38 | 0.817506268 | 0.702401108 | 0.951474152 | 0.009257059 |
| ACACB | 1.821114863 | 1.156288839 | 2.86819282 | 0.009694141 |
| COL4A6 | 1.470074673 | 1.097499529 | 1.969130271 | 0.009770862 |
| SSNA1 | 0.553521627 | 0.353404187 | 0.866956881 | 0.009777636 |
| CLDN4 | 0.71015941 | 0.547124335 | 0.92177656 | 0.010109426 |
| BHLHE22 | 1.461694337 | 1.094523635 | 1.952036727 | 0.010113866 |
| DNMT1 | 0.671509867 | 0.495062556 | 0.910845502 | 0.010456589 |
| SH3BGRL | 1.312430615 | 1.065017543 | 1.617319949 | 0.010741527 |
| AIM2 | 0.861582008 | 0.76821644 | 0.966294808 | 0.010901325 |
| SLC25A10 | 0.651273612 | 0.467918365 | 0.906477175 | 0.011021475 |
| SAPCD2 | 0.75541431 | 0.608202719 | 0.938257529 | 0.011205345 |
| METTL13 | 0.493521367 | 0.285599863 | 0.852813223 | 0.011390809 |
| WDR43 | 0.66880899 | 0.489564422 | 0.913680497 | 0.011501089 |
| POLR3G | 0.609172058 | 0.414443487 | 0.895394928 | 0.011661989 |
| MITF | 1.438821963 | 1.083205972 | 1.91118651 | 0.012013586 |
| ASPA | 1.393176425 | 1.075270333 | 1.805072169 | 0.012103305 |
| ABI3BP | 1.31915783 | 1.061834405 | 1.638840645 | 0.012353494 |
| GAPDH | 0.581531169 | 0.379629234 | 0.89081259 | 0.012726416 |
| PPM1K | 1.498822471 | 1.089778191 | 2.061400034 | 0.012821657 |
| PTCH1 | 1.852293423 | 1.13755927 | 3.016098602 | 0.013208557 |
| GBP5 | 0.854123833 | 0.753728977 | 0.967891039 | 0.01345453 |
| SNORA5B | 0.660387405 | 0.474906184 | 0.918310898 | 0.013642103 |
| UBE2L6 | 0.785808505 | 0.648737365 | 0.951841284 | 0.013715299 |
| CXCL9 | 0.87877451 | 0.792896188 | 0.973954285 | 0.013779857 |
| P2RY12 | 1.334024303 | 1.059762687 | 1.679263539 | 0.014117259 |
| AVL9 | 0.39796227 | 0.190639079 | 0.830752904 | 0.014137101 |
| DCAF13 | 0.55514274 | 0.346352923 | 0.889796047 | 0.014482891 |
| OTUD4 | 0.467655324 | 0.254253533 | 0.860170948 | 0.014509148 |
| LAP3 | 0.719180695 | 0.552091154 | 0.936839628 | 0.014541364 |
| FAS | 0.811046065 | 0.684992928 | 0.960295637 | 0.015096508 |
| FBXL5 | 1.480926309 | 1.077844416 | 2.034748895 | 0.015417437 |
| IL12RB1 | 0.514032683 | 0.299895496 | 0.881072249 | 0.0154991 |
| PUSL1 | 0.656760794 | 0.466899657 | 0.923827494 | 0.015731791 |
| POLR1B | 0.560471471 | 0.34916841 | 0.899646878 | 0.016486499 |
| ADH5 | 1.593072344 | 1.086890107 | 2.334991804 | 0.016982799 |
| JAK2 | 0.788964122 | 0.649230096 | 0.958773153 | 0.017159742 |
| PRKCB | 1.480466802 | 1.071652159 | 2.045236351 | 0.017327853 |
| PROSER1 | 0.693765495 | 0.513050218 | 0.938135383 | 0.017560944 |
| GGTA1P | 1.189797142 | 1.029934361 | 1.374473261 | 0.018244577 |
| YME1L1 | 0.310508023 | 0.116952869 | 0.824393903 | 0.01889586 |
| ATAD3A | 0.52163358 | 0.302515665 | 0.899462814 | 0.019225335 |
| TACO1 | 0.426779073 | 0.208846609 | 0.872125137 | 0.019533099 |
| SP110 | 0.711587134 | 0.53373227 | 0.948708328 | 0.020406413 |
| PPP4C | 0.624115017 | 0.417797446 | 0.932316745 | 0.021322884 |
| SLC1A5 | 0.686680505 | 0.497526648 | 0.947748462 | 0.022230959 |
| KCNN3 | 1.479259538 | 1.057482617 | 2.069262175 | 0.022234991 |
| LOC101060363 | 0.424124331 | 0.202637557 | 0.887700436 | 0.022842273 |
| GGCT | 0.682277588 | 0.490851347 | 0.948357807 | 0.022872411 |
| AURKAIP1 | 0.406595427 | 0.187085559 | 0.88365902 | 0.023071152 |
| CFD | 1.164093015 | 1.020343104 | 1.328094975 | 0.023856179 |
| RCBTB2 | 1.692610482 | 1.069451406 | 2.678878375 | 0.024665425 |
| RCCD1 | 0.512918212 | 0.285628392 | 0.921074724 | 0.025403468 |
| BRMS1 | 0.618469952 | 0.404874496 | 0.944749758 | 0.02622311 |
| PTPN22 | 0.711717485 | 0.527072904 | 0.961046896 | 0.026469969 |
| CXCL5 | 0.905776784 | 0.829800613 | 0.988709298 | 0.026828939 |
| SAMD9L | 0.83679604 | 0.714664491 | 0.979799082 | 0.026863722 |
| ADGRL3 | 1.279309404 | 1.027879339 | 1.5922419 | 0.027365951 |
| SURF6 | 0.703889198 | 0.515147027 | 0.961783679 | 0.027481338 |
| RNF149 | 0.644667525 | 0.435571412 | 0.954140254 | 0.028189632 |
| POLR1A | 0.580462935 | 0.356570045 | 0.944939778 | 0.028687561 |
| RIPK2 | 0.736237066 | 0.558429553 | 0.970659619 | 0.029922632 |
| ZBP1 | 0.681284798 | 0.480874304 | 0.96521892 | 0.030840298 |
| IL2RB | 0.802964716 | 0.657902905 | 0.980011383 | 0.030883196 |
| CXCL8 | 0.919716802 | 0.851404523 | 0.993510103 | 0.033560459 |
| ECE2 | 0.514506676 | 0.278743452 | 0.949680138 | 0.033581208 |
| FASLG | 0.708346369 | 0.515010962 | 0.974259997 | 0.033979744 |
| HDGF | 0.685107812 | 0.482666145 | 0.972458332 | 0.034323881 |
| WRNIP1 | 0.445080983 | 0.209334241 | 0.946319533 | 0.03543733 |
| POLR2D | 0.677213834 | 0.470111393 | 0.975552994 | 0.036361334 |
| TXLNB | 1.651414691 | 1.030554109 | 2.646314694 | 0.037063649 |
| UMPS | 0.556956655 | 0.321039144 | 0.96623954 | 0.037329304 |
| SNX20 | 0.622120506 | 0.397800404 | 0.972934969 | 0.037505141 |
| TXNIP | 1.233230926 | 1.011675711 | 1.503306347 | 0.038000158 |
| CEBPB | 1.338218835 | 1.01619693 | 1.762286026 | 0.038045554 |
| IL21R | 0.718261576 | 0.524672766 | 0.983278958 | 0.038904259 |
| COASY | 0.67071531 | 0.458411424 | 0.981343405 | 0.039690797 |
| IFIT3 | 0.852608053 | 0.731585187 | 0.993651191 | 0.041200466 |
| WDR5 | 0.708650607 | 0.508176208 | 0.988211717 | 0.042371064 |
| FLJ32255 | 0.828796488 | 0.691310605 | 0.993625172 | 0.042451383 |
| ATP6V1B2 | 0.712334375 | 0.510689587 | 0.993598215 | 0.045739545 |
| VARS | 0.631469415 | 0.402047661 | 0.991806844 | 0.045968908 |
| FGFR1OP | 0.63185582 | 0.401691652 | 0.993901105 | 0.046985174 |
| TIGIT | 0.79904553 | 0.636959282 | 1.002377668 | 0.052448628 |
| CPSF3L | 0.625931051 | 0.389635303 | 1.005529214 | 0.052725133 |
| URB2 | 0.703356126 | 0.492215871 | 1.00506682 | 0.053333259 |
| CD74 | 0.733693918 | 0.535104526 | 1.005984325 | 0.054490862 |
| RTN1 | 1.15412679 | 0.996366016 | 1.336866799 | 0.05595083 |
| PDCD1LG2 | 0.646613988 | 0.412925066 | 1.01255575 | 0.056723626 |
| DARS2 | 0.769601698 | 0.587499957 | 1.008147774 | 0.057294514 |
| LILRB5 | 0.733751648 | 0.532597504 | 1.010878715 | 0.05825467 |
| TLR8 | 0.846772282 | 0.7127585 | 1.00598351 | 0.058477882 |
| GK | 0.800706632 | 0.63567884 | 1.008577084 | 0.059101444 |
| SGTB | 1.516967385 | 0.982673043 | 2.341765721 | 0.059963252 |
| NAPSB | 1.175018528 | 0.993076157 | 1.390294724 | 0.060242027 |
| IRF2 | 0.715989148 | 0.505307906 | 1.014511061 | 0.060252748 |
| CD83 | 1.252380761 | 0.99005835 | 1.584207205 | 0.060566575 |
| MMP3 | 0.935713121 | 0.872921497 | 1.003021518 | 0.060815025 |
| GNG7 | 1.490302961 | 0.981237427 | 2.263471464 | 0.061326338 |
| ZHX1-C8orf76 | 0.731187479 | 0.525894695 | 1.016620123 | 0.062612168 |
| PRPF19 | 0.720338397 | 0.509424166 | 1.018576346 | 0.063477274 |
| CHST11 | 1.21630661 | 0.987330484 | 1.498385589 | 0.065746861 |
| SLC31A2 | 0.805125891 | 0.639082469 | 1.01430994 | 0.065857715 |
| TLR7 | 1.260188336 | 0.98413197 | 1.613680574 | 0.066777012 |
| LDLRAD4 | 1.398514933 | 0.975256677 | 2.005465907 | 0.068192243 |
| GZMA | 0.884702687 | 0.775474203 | 1.009316417 | 0.068449043 |
| CLPB | 0.609953567 | 0.357024583 | 1.042066491 | 0.070424545 |
| EBP | 0.717046557 | 0.49930049 | 1.029752174 | 0.071671656 |
| HSPA6 | 1.192935105 | 0.984127103 | 1.446047121 | 0.072337946 |
| USP39 | 0.645539358 | 0.397392892 | 1.048637435 | 0.077043165 |
| UBE2C | 0.86650778 | 0.738668108 | 1.016472384 | 0.078517839 |
| SLC2A5 | 0.648892128 | 0.39874907 | 1.055954799 | 0.08171596 |
| GK3P | 0.853162989 | 0.712230992 | 1.021981763 | 0.084721708 |
| MYBL2 | 0.797180517 | 0.615613987 | 1.032297494 | 0.085629429 |
| ITM2A | 1.132672589 | 0.982576111 | 1.305697524 | 0.085866222 |
| FCAR | 0.562579292 | 0.291671072 | 1.0851109 | 0.086115496 |
| PSMG3 | 0.732956948 | 0.513567264 | 1.046067235 | 0.086932884 |
| STAT2 | 0.619815267 | 0.358175972 | 1.072576038 | 0.087347493 |
| ZNF593 | 0.755634544 | 0.546629337 | 1.044553458 | 0.089865838 |
| TRUB2 | 0.705697894 | 0.470888194 | 1.0575961 | 0.091282014 |
| BCL2 | 1.678815371 | 0.919688465 | 3.064538871 | 0.091544786 |
| ACTL6A | 0.766838842 | 0.563183418 | 1.044139068 | 0.09185278 |
| TEK | 1.409022188 | 0.945142261 | 2.1005764 | 0.092367686 |
| MRPS17 | 0.786617905 | 0.594511147 | 1.040800887 | 0.09294977 |
| SLC9A9 | 1.35627862 | 0.949743863 | 1.93682925 | 0.093673891 |
| DPH2 | 0.701573348 | 0.462580452 | 1.064042289 | 0.095344604 |
| CTSO | 1.18372306 | 0.970115224 | 1.4443648 | 0.096683987 |
| ZMYND19 | 0.783146551 | 0.586806752 | 1.045179726 | 0.096936533 |
| IFIT2 | 0.871633444 | 0.740415409 | 1.026106224 | 0.098864982 |
| CFH | 1.110678649 | 0.980143103 | 1.25859893 | 0.099856232 |
| FCGR3B | 0.916734858 | 0.82571055 | 1.017793462 | 0.103227374 |
| POLR1C | 0.790411266 | 0.594665591 | 1.050590415 | 0.105225156 |
| NKG7 | 0.871434383 | 0.737107405 | 1.030240475 | 0.107144224 |
| BTN2A2 | 0.638007584 | 0.369219441 | 1.102470864 | 0.10731254 |
| RITA1 | 0.678306086 | 0.422411154 | 1.089221111 | 0.108208926 |
| LILRB2 | 0.837872102 | 0.675019468 | 1.040013943 | 0.108678489 |
| MYO1G | 1.359890805 | 0.932372009 | 1.983438994 | 0.110413807 |
| SLFN5 | 0.794937506 | 0.598553172 | 1.055755223 | 0.112922448 |
| CLEC12A | 0.669432092 | 0.406991627 | 1.101102076 | 0.113961067 |
| PRF1 | 0.822346051 | 0.645049284 | 1.0483742 | 0.114410335 |
| POLD2 | 0.769660499 | 0.55592006 | 1.065579975 | 0.114730216 |
| CBFB | 0.744946485 | 0.516487664 | 1.074459864 | 0.115107519 |
| PPP5C | 0.575229428 | 0.288418777 | 1.147251573 | 0.116423473 |
| HCAR3 | 0.944460607 | 0.878806975 | 1.01501907 | 0.120081123 |
| PTGDS | 1.117037295 | 0.971312095 | 1.284625533 | 0.120698755 |
| SAC3D1 | 0.790211488 | 0.586710204 | 1.064297487 | 0.121190074 |
| NUP62 | 0.654011263 | 0.382203148 | 1.119118811 | 0.121300747 |
| LMTK2 | 0.574920978 | 0.28432393 | 1.162526598 | 0.12337278 |
| FAM26F | 0.863090522 | 0.715526516 | 1.041086854 | 0.12378769 |
| ST8SIA1 | 1.251835332 | 0.935101358 | 1.675852233 | 0.131266439 |
| GK6P | 0.84463405 | 0.676669315 | 1.054291458 | 0.13553828 |
| HLA-DRA | 0.893201853 | 0.769431538 | 1.036881789 | 0.137792482 |
| CD300LF | 0.868062754 | 0.71849764 | 1.048761892 | 0.142511668 |
| SLC2A3 | 1.11355827 | 0.96418193 | 1.286076809 | 0.143295757 |
| SLC5A6 | 0.808588819 | 0.607621685 | 1.076024595 | 0.145017009 |
| NOL10 | 0.676829918 | 0.398571797 | 1.14935061 | 0.148527492 |
| TNFSF13B | 0.900225387 | 0.779850441 | 1.039180981 | 0.151231844 |
| LILRB4 | 0.689435256 | 0.414585572 | 1.146496657 | 0.151823032 |
| POLDIP2 | 0.706184402 | 0.438621654 | 1.136962584 | 0.152230638 |
| SIGLEC10 | 0.822488564 | 0.628882478 | 1.075697705 | 0.153553317 |
| PPBP | 1.059995842 | 0.978076415 | 1.148776483 | 0.155668589 |
| RELT | 0.66382954 | 0.37669289 | 1.169838005 | 0.15638467 |
| NIN | 1.355400368 | 0.889959817 | 2.064261918 | 0.156537659 |
| RNF114 | 0.744566546 | 0.49510714 | 1.119715908 | 0.156539107 |
| OSM | 0.874698716 | 0.724523569 | 1.056001318 | 0.163621455 |
| SGO1 | 0.769149495 | 0.531543085 | 1.112968943 | 0.163851864 |
| ICOS | 0.848820247 | 0.673730436 | 1.069412591 | 0.164345804 |
| SLC2A14 | 1.110615428 | 0.955465427 | 1.290958933 | 0.171762008 |
| REXO4 | 0.732770945 | 0.46896152 | 1.144983618 | 0.172126565 |
| DPYD | 0.814822417 | 0.6066215 | 1.094480778 | 0.17374117 |
| VAV1 | 1.198929689 | 0.923106072 | 1.557169261 | 0.173786513 |
| P2RY13 | 0.896085992 | 0.764955907 | 1.049694626 | 0.174093378 |
| CCNI | 1.375059978 | 0.868090968 | 2.178101161 | 0.174723961 |
| ACP5 | 0.870024361 | 0.71084841 | 1.064843612 | 0.176841363 |
| CD300A | 0.806634602 | 0.590092382 | 1.102639859 | 0.177872186 |
| CYBB | 0.801343505 | 0.580282241 | 1.106619103 | 0.17869322 |
| FLAD1 | 0.777846983 | 0.539316889 | 1.121874618 | 0.178785605 |
| B2M | 0.728794133 | 0.456954586 | 1.162349399 | 0.184077677 |
| TARBP2 | 0.647517629 | 0.340665578 | 1.230764441 | 0.184735549 |
| CMKLR1 | 0.762282714 | 0.509990463 | 1.139383928 | 0.185619637 |
| GBP2 | 0.87552806 | 0.717643203 | 1.068148325 | 0.190136978 |
| TBX21 | 0.7959964 | 0.563894479 | 1.123632687 | 0.194556347 |
| C3AR1 | 1.123183148 | 0.94221482 | 1.338909511 | 0.194995003 |
| SLAMF8 | 0.88238439 | 0.728764076 | 1.068387201 | 0.199792484 |
| CAPN13 | 1.157560003 | 0.92529472 | 1.448127966 | 0.200379552 |
| PSMB8-AS1 | 0.759404299 | 0.498286357 | 1.157356371 | 0.200475719 |
| CSNK2B | 0.772233046 | 0.514619003 | 1.158806563 | 0.211961572 |
| LOC100507616 | 1.370494523 | 0.827888405 | 2.268729972 | 0.220376756 |
| TLR2 | 1.110840696 | 0.938681601 | 1.314574665 | 0.221156182 |
| CD163 | 1.130029857 | 0.928603787 | 1.375147824 | 0.222296953 |
| SDCCAG3 | 0.65223523 | 0.328098235 | 1.296595806 | 0.222830276 |
| DHRS13 | 0.83384167 | 0.621212927 | 1.119248974 | 0.226329475 |
| FCRL1 | 1.236504904 | 0.876544371 | 1.744286346 | 0.226535833 |
| VPREB3 | 1.126698694 | 0.925544719 | 1.371570624 | 0.234492849 |
| MRC1 | 1.087132524 | 0.946975434 | 1.248033563 | 0.235496801 |
| PIK3IP1 | 1.342661985 | 0.820750833 | 2.196453704 | 0.240654998 |
| STX11 | 0.83667257 | 0.619457276 | 1.130055319 | 0.244936754 |
| APOBEC3F | 0.79293178 | 0.534604486 | 1.17608592 | 0.248678302 |
| METTL26 | 0.669201821 | 0.337357405 | 1.327467755 | 0.250400284 |
| SET | 0.711106371 | 0.397339681 | 1.27264478 | 0.250936015 |
| CTSL | 1.126017353 | 0.917859048 | 1.381383211 | 0.255086227 |
| CYSLTR1 | 1.145405433 | 0.903972915 | 1.451319596 | 0.260986246 |
| FPR3 | 0.908396342 | 0.767708731 | 1.074865873 | 0.263119868 |
| SIGLEC7 | 0.712121745 | 0.391168133 | 1.296417926 | 0.266706662 |
| CXCL2 | 0.867623289 | 0.674814941 | 1.115520903 | 0.268121905 |
| PLEK | 0.9165252 | 0.78457027 | 1.070673304 | 0.271772688 |
| TRIM11 | 0.79240658 | 0.522577043 | 1.201560987 | 0.273311676 |
| NOC2L | 0.752986576 | 0.450423931 | 1.258789207 | 0.279198346 |
| CD226 | 0.807558199 | 0.545890179 | 1.194654659 | 0.284718309 |
| MTX1 | 0.819860761 | 0.569605211 | 1.180065867 | 0.285107609 |
| TLR4 | 0.899869832 | 0.74138832 | 1.092228855 | 0.285782821 |
| SEC61A2 | 1.420954824 | 0.742667257 | 2.71873116 | 0.288565903 |
| CLEC4A | 0.925865104 | 0.80284107 | 1.06774083 | 0.28964415 |
| MMRN1 | 1.070347708 | 0.94360669 | 1.214112011 | 0.290394821 |
| BRAT1 | 0.808187163 | 0.54296788 | 1.202956039 | 0.293988214 |
| LOC285957 | 0.686422627 | 0.339917668 | 1.386147492 | 0.29402574 |
| MIR6834 | 0.754890608 | 0.444461355 | 1.282135834 | 0.298156359 |
| CD14 | 1.094541831 | 0.921987329 | 1.299390764 | 0.302057206 |
| PILRA | 0.889744984 | 0.712463951 | 1.111138515 | 0.30281564 |
| CSTF1 | 0.818892193 | 0.559415233 | 1.198723927 | 0.30410263 |
| PUS7 | 0.879033949 | 0.687062875 | 1.124643335 | 0.305087602 |
| NCF1 | 0.890879026 | 0.7138362 | 1.111831312 | 0.306699926 |
| STAT4 | 1.118723821 | 0.898722771 | 1.392579589 | 0.315289556 |
| CASP4 | 0.871015318 | 0.665012891 | 1.140831546 | 0.315860984 |
| DOK2 | 0.825038979 | 0.562362059 | 1.210411166 | 0.325375759 |
| PGP | 0.791128759 | 0.493259036 | 1.268876325 | 0.331039455 |
| HLX | 1.177054399 | 0.84699262 | 1.63573687 | 0.331595877 |
| JMJD4 | 0.70812231 | 0.352661491 | 1.421865495 | 0.331857768 |
| ITPRIP | 1.191835966 | 0.831924793 | 1.707453582 | 0.338687385 |
| NCF1C | 0.907925844 | 0.743582611 | 1.108591468 | 0.343081942 |
| LOC101928927 | 0.868337257 | 0.647519184 | 1.164459079 | 0.34569494 |
| SMG7 | 0.666680468 | 0.286791106 | 1.549779045 | 0.346176889 |
| MRPS26 | 0.798052249 | 0.498775318 | 1.276902382 | 0.346875204 |
| FPR1 | 0.892617505 | 0.702750607 | 1.13378203 | 0.351871006 |
| TNF | 0.87336981 | 0.65537921 | 1.1638679 | 0.3553963 |
| EGFL6 | 0.944089134 | 0.834516472 | 1.068048772 | 0.360684961 |
| TLR1 | 0.93618596 | 0.811653362 | 1.07982569 | 0.365235807 |
| ARMC2-AS1 | 0.829640541 | 0.552374238 | 1.246081697 | 0.36817493 |
| SNRPA1 | 0.850158877 | 0.596904089 | 1.210864742 | 0.368323698 |
| LINC00849 | 1.109960865 | 0.881289031 | 1.397967158 | 0.375436442 |
| LSM14B | 0.774766427 | 0.439262133 | 1.366525754 | 0.378095115 |
| LOC101927673 | 0.730338302 | 0.36245062 | 1.471632289 | 0.379347881 |
| CFLAR | 0.82563949 | 0.538450769 | 1.266003517 | 0.379675202 |
| HAVCR2 | 0.830692429 | 0.543464677 | 1.269723573 | 0.391517676 |
| P2RY14 | 1.056625477 | 0.930652694 | 1.199649886 | 0.395113072 |
| PRKACB | 1.070870871 | 0.913414683 | 1.255469661 | 0.398755809 |
| TRAF1 | 1.293750913 | 0.710544046 | 2.355647668 | 0.39960593 |
| TPD52L2 | 1.150701644 | 0.828754971 | 1.59771503 | 0.40187665 |
| SELP | 1.072831777 | 0.909014779 | 1.266170856 | 0.405647416 |
| ALG3 | 0.839779896 | 0.552811441 | 1.27571577 | 0.41306296 |
| LOC101928173 | 0.903870486 | 0.706638578 | 1.156152356 | 0.420989073 |
| SPECC1L-ADORA2A | 1.129292996 | 0.838101768 | 1.521656104 | 0.424197697 |
| BYSL | 0.9083501 | 0.716794409 | 1.151097016 | 0.426333867 |
| MS4A1 | 1.047083278 | 0.933935542 | 1.173939037 | 0.430378837 |
| DRAM1 | 0.91943836 | 0.745820824 | 1.133471835 | 0.431504705 |
| TARP | 0.915281314 | 0.734055281 | 1.141249039 | 0.431668782 |
| PDRG1 | 1.138182497 | 0.821780965 | 1.576404724 | 0.436066267 |
| MSR1 | 1.100690278 | 0.859465706 | 1.409618883 | 0.447196971 |
| ISG20L2 | 0.818731216 | 0.488406892 | 1.37246385 | 0.447983434 |
| RHEBL1 | 0.886031957 | 0.647754673 | 1.211959806 | 0.448979773 |
| GZMH | 0.951668587 | 0.836098407 | 1.083213521 | 0.453298272 |
| ADAM28 | 1.08179005 | 0.880759371 | 1.328705378 | 0.453560104 |
| TPRA1 | 1.179783382 | 0.762420012 | 1.825619482 | 0.457957146 |
| TOMM34 | 0.890327595 | 0.652922634 | 1.214053834 | 0.462860801 |
| FCGR1CP | 0.950518166 | 0.829960385 | 1.088587841 | 0.463342677 |
| IL1A | 0.931744721 | 0.771309917 | 1.125550452 | 0.463402174 |
| KAT2B | 1.084616901 | 0.872750115 | 1.347915974 | 0.463848362 |
| FGR | 0.92389392 | 0.744332659 | 1.146772165 | 0.472813041 |
| TOX | 0.936340953 | 0.781375637 | 1.122039564 | 0.476122034 |
| XPO5 | 0.909539052 | 0.70049099 | 1.180973488 | 0.476713309 |
| TMEM154 | 0.935144135 | 0.776587972 | 1.126072751 | 0.479330507 |
| PSMA7 | 0.876846997 | 0.608732664 | 1.26305142 | 0.480312809 |
| ITGAX | 0.823927508 | 0.479895601 | 1.414592126 | 0.482505228 |
| DSE | 1.296584682 | 0.623747153 | 2.695213643 | 0.486621526 |
| OLR1 | 1.038475116 | 0.933220346 | 1.155601217 | 0.488684027 |
| TAGAP | 0.919217614 | 0.724162175 | 1.166811869 | 0.488818201 |
| CSF2RB | 0.951307723 | 0.82560652 | 1.096147332 | 0.48996986 |
| TYROBP | 1.058363064 | 0.900346343 | 1.244112763 | 0.49173774 |
| FCER1G | 0.952406 | 0.826430329 | 1.097584584 | 0.500530325 |
| MNDA | 0.961144017 | 0.855044376 | 1.080409212 | 0.506653644 |
| CLEC7A | 0.917846357 | 0.712597189 | 1.182213385 | 0.506814365 |
| RGL4 | 0.894273258 | 0.642751002 | 1.244221567 | 0.507222703 |
| LCP1 | 0.941231922 | 0.786627666 | 1.126222188 | 0.508253652 |
| TFEC | 0.939455844 | 0.780416889 | 1.130904898 | 0.509265594 |
| UBAP2L | 0.856873152 | 0.537378584 | 1.366320916 | 0.516433141 |
| KCTD12 | 1.054413386 | 0.896559655 | 1.240059802 | 0.521948725 |
| SELL | 0.958791158 | 0.839876061 | 1.094543025 | 0.53337287 |
| IGSF6 | 0.947181214 | 0.797749747 | 1.124603618 | 0.535618485 |
| SAMHD1 | 1.089027984 | 0.83081472 | 1.427492701 | 0.536807316 |
| CD84 | 0.876756396 | 0.577503995 | 1.33107612 | 0.536949797 |
| MTG2 | 0.804634181 | 0.402296823 | 1.609349436 | 0.538825273 |
| LILRB3 | 0.88304656 | 0.591494015 | 1.318307889 | 0.542966544 |
| MTBP | 0.793570933 | 0.376335452 | 1.673386923 | 0.543576765 |
| CEMIP | 0.946671457 | 0.792659998 | 1.130606881 | 0.545217068 |
| NCF2 | 0.957698713 | 0.831461858 | 1.103101503 | 0.548954388 |
| EFNA4 | 0.930676785 | 0.735607637 | 1.177474558 | 0.549410321 |
| CCKBR | 1.136965248 | 0.743570809 | 1.738489408 | 0.553549372 |
| SH2B3 | 1.083735894 | 0.826088342 | 1.421740787 | 0.561522984 |
| SCYL1 | 0.781231269 | 0.339183686 | 1.799385764 | 0.56193661 |
| THEMIS2 | 1.059255897 | 0.868903833 | 1.291308673 | 0.568959868 |
| CPA3 | 1.02715395 | 0.936262671 | 1.126868847 | 0.570876464 |
| RNASE2 | 0.939893199 | 0.757765773 | 1.165794571 | 0.572706039 |
| ENPP2 | 1.040497615 | 0.90439146 | 1.19708703 | 0.578884097 |
| BIN2 | 0.93785608 | 0.747374849 | 1.176884701 | 0.579655812 |
| PPP3CC | 1.169059972 | 0.671446246 | 2.035458871 | 0.580885857 |
| IGFLR1 | 0.914331472 | 0.66186898 | 1.263092947 | 0.586956486 |
| LILRB1 | 0.913034642 | 0.657154432 | 1.268548482 | 0.58764865 |
| FCGR1B | 0.965727807 | 0.84928621 | 1.098134158 | 0.594748121 |
| WISP1 | 1.053471722 | 0.864402983 | 1.283895002 | 0.605755096 |
| HS3ST3B1 | 1.120283901 | 0.725851218 | 1.729054095 | 0.607985462 |
| PIK3R5 | 1.147841041 | 0.673314363 | 1.956796302 | 0.612419893 |
| GAPT | 1.042984208 | 0.885946525 | 1.227857469 | 0.61321959 |
| GM2A | 0.91444303 | 0.640059444 | 1.306450617 | 0.623162554 |
| STK10 | 1.113630866 | 0.722372519 | 1.716806321 | 0.626013671 |
| CSE1L | 1.080409488 | 0.788975138 | 1.47949486 | 0.629665728 |
| CD53 | 1.085029479 | 0.773877522 | 1.521285911 | 0.636007788 |
| KMO | 0.948998113 | 0.763002327 | 1.180333777 | 0.638117194 |
| MDC1 | 0.917708936 | 0.640940298 | 1.313990855 | 0.639134968 |
| METTL1 | 0.912319383 | 0.614153885 | 1.355241214 | 0.649486329 |
| TNFSF4 | 1.038282794 | 0.881325457 | 1.223193034 | 0.653243411 |
| FCGR2A | 1.066231157 | 0.803256263 | 1.415300363 | 0.65717836 |
| GPR65 | 0.965967673 | 0.828199091 | 1.126653669 | 0.659194599 |
| WIPF1 | 1.051192685 | 0.841130118 | 1.313715961 | 0.660714066 |
| IL18RAP | 0.952952606 | 0.766755793 | 1.184364927 | 0.66395224 |
| WDR46 | 0.916842765 | 0.618665513 | 1.358732041 | 0.665323338 |
| C5AR1 | 1.037181533 | 0.873548529 | 1.231466252 | 0.676874187 |
| NLRC4 | 0.904031842 | 0.561632274 | 1.455175581 | 0.677842097 |
| TRIB3 | 1.044292785 | 0.850933848 | 1.281588955 | 0.678253058 |
| CYTH4 | 0.84270079 | 0.373232936 | 1.902684767 | 0.680431105 |
| PRR11 | 0.923411859 | 0.626708728 | 1.36058335 | 0.68700589 |
| ADRM1 | 0.934335445 | 0.668670562 | 1.305549807 | 0.690692414 |
| SPNS1 | 1.082330089 | 0.732539988 | 1.599146043 | 0.691188845 |
| SAMSN1 | 0.963109947 | 0.799764933 | 1.159816756 | 0.69181111 |
| LAT2 | 1.09373007 | 0.698303288 | 1.713074372 | 0.695532239 |
| YDJC | 0.93579824 | 0.66666963 | 1.313571679 | 0.701333149 |
| SEC14L1 | 1.075350323 | 0.741504161 | 1.559503478 | 0.70168912 |
| SCAMP3 | 0.919365008 | 0.590695466 | 1.430909948 | 0.709536306 |
| ST8SIA4 | 0.956257357 | 0.755263292 | 1.210740865 | 0.710245343 |
| KLRB1 | 1.028236871 | 0.887280436 | 1.191586131 | 0.711263287 |
| SLA2 | 0.912212561 | 0.558239058 | 1.490636933 | 0.713835594 |
| RAE1 | 1.073828375 | 0.718176463 | 1.605604525 | 0.728552378 |
| RAB8B | 1.04333101 | 0.819169848 | 1.328832596 | 0.73106023 |
| DHCR7 | 0.960185282 | 0.760515479 | 1.212277462 | 0.732670472 |
| PFDN2 | 1.057968408 | 0.758167065 | 1.476319936 | 0.740293047 |
| NCF4 | 0.964772917 | 0.779596936 | 1.193933352 | 0.741537998 |
| LINC01094 | 1.027122378 | 0.875862607 | 1.204504418 | 0.741971999 |
| NSDHL | 0.946819351 | 0.677308267 | 1.323572923 | 0.7491677 |
| CDK5RAP1 | 0.942089201 | 0.641356396 | 1.38383599 | 0.761068823 |
| RPP40 | 0.961469613 | 0.745501447 | 1.240002713 | 0.762109909 |
| CPSF4 | 0.937403933 | 0.61188978 | 1.436085653 | 0.766457418 |
| PDE4B | 0.973022975 | 0.81177136 | 1.16630588 | 0.767363506 |
| DAP3 | 0.87363777 | 0.35260984 | 2.164553755 | 0.770423341 |
| ETS1 | 1.044626321 | 0.772652346 | 1.412335258 | 0.776612918 |
| PTPRC | 0.970481935 | 0.786611273 | 1.197332429 | 0.779809908 |
| LINC01093 | 0.930917728 | 0.558802271 | 1.55083088 | 0.783392089 |
| FYB | 0.972163254 | 0.787295823 | 1.200439993 | 0.793058903 |
| GLUL | 1.032562854 | 0.812612328 | 1.312047591 | 0.793179429 |
| IFI16 | 0.978107278 | 0.820592808 | 1.165856973 | 0.804845177 |
| ALOX5AP | 0.982682648 | 0.854287988 | 1.130374299 | 0.806819582 |
| RPRD1B | 0.91592748 | 0.450058861 | 1.864029844 | 0.808600176 |
| MRPL9 | 1.054011879 | 0.688056443 | 1.614607424 | 0.808977713 |
| IL24 | 1.012876726 | 0.910176033 | 1.127165763 | 0.814553285 |
| MIEF1 | 0.944873606 | 0.586657011 | 1.521819588 | 0.815618536 |
| FCGR2C | 0.963212563 | 0.701809405 | 1.321980634 | 0.816519164 |
| SLAMF9 | 0.947866222 | 0.600067564 | 1.497248691 | 0.818447293 |
| COL7A1 | 0.974353793 | 0.779811186 | 1.217429721 | 0.81915525 |
| GTF2H4 | 0.95600038 | 0.646718653 | 1.413190608 | 0.821477985 |
| JAK3 | 0.917883882 | 0.420485567 | 2.003661685 | 0.82967119 |
| RILPL2 | 1.031341151 | 0.773633034 | 1.374895492 | 0.833380315 |
| SETDB1 | 0.946995218 | 0.551627816 | 1.625733724 | 0.843423226 |
| MIR155 | 0.985634502 | 0.845264424 | 1.149315343 | 0.853548754 |
| HCK | 0.98407484 | 0.823278628 | 1.176276485 | 0.860010948 |
| FCN1 | 0.973567332 | 0.719780797 | 1.316836116 | 0.861988907 |
| ITGB2 | 0.982110883 | 0.786509692 | 1.226357152 | 0.873435951 |
| TGIF2-C20orf24 | 0.972613526 | 0.686983543 | 1.377001067 | 0.875607542 |
| FGD3 | 0.961084009 | 0.554652722 | 1.665334787 | 0.887457119 |
| ITGAM | 1.021984955 | 0.73466794 | 1.42166711 | 0.897256672 |
| CCDC71L | 1.030366994 | 0.649564528 | 1.634412126 | 0.898874088 |
| RAC2 | 0.985433136 | 0.771256696 | 1.259085944 | 0.9065739 |
| SLA | 1.012104935 | 0.826593048 | 1.239251168 | 0.907278022 |
| NPL | 0.981995495 | 0.710994207 | 1.356291152 | 0.912192638 |
| PSMD4 | 0.965266514 | 0.512477577 | 1.818107728 | 0.912859625 |
| FAM58A | 1.021388621 | 0.675793515 | 1.543718152 | 0.920006373 |
| CST7 | 1.009270203 | 0.835085537 | 1.219786834 | 0.923946107 |
| UBL4A | 0.984440737 | 0.686546609 | 1.411591802 | 0.932037793 |
| IL1RAP | 0.989326555 | 0.764320953 | 1.28057072 | 0.935038094 |
| MRGBP | 0.989994456 | 0.775794038 | 1.263336625 | 0.935570864 |
| LAPTM5 | 1.007439651 | 0.835109522 | 1.215331191 | 0.938276144 |
| CLEC10A | 0.992156637 | 0.807134939 | 1.219591352 | 0.940391837 |
| LCP2 | 1.008570113 | 0.80369642 | 1.265669036 | 0.941281734 |
| SF3B4 | 1.014626416 | 0.679879415 | 1.514190223 | 0.94333009 |
| MIR3945HG | 1.009323631 | 0.780260369 | 1.305633649 | 0.943665682 |
| SRGN | 1.007155311 | 0.819761143 | 1.237387048 | 0.945882636 |
| CSF2RA | 1.008122204 | 0.784430229 | 1.295603279 | 0.949610851 |
| LAIR1 | 1.008694378 | 0.731588063 | 1.390761277 | 0.957871548 |
| CD69 | 0.997007557 | 0.884259721 | 1.124131343 | 0.960962424 |
| CCL13 | 1.006692135 | 0.721155608 | 1.405284856 | 0.968738823 |
| DOK3 | 0.993499476 | 0.700298338 | 1.409458163 | 0.970844133 |
| SLC1A3 | 0.996455278 | 0.773214796 | 1.284149146 | 0.978109464 |
| LST1 | 1.002651872 | 0.823627052 | 1.220589796 | 0.978945642 |
| CCR1 | 1.000741017 | 0.831199116 | 1.204864832 | 0.993759625 |
| SLC15A3 | 1.000888574 | 0.768349422 | 1.303805156 | 0.994746772 |
| EIF4E3 | 1.000460456 | 0.786743517 | 1.272233075 | 0.997004305 |
| MYO1F | 1.000038192 | 0.780676874 | 1.281037545 | 0.999758815 |
