# Supplementary material for: Immunogenomic characterization in gastric cancer identifies microenvironmental and immunotherapeutically relevant gene signatures
Source: Immun Inflamm Dis. 2021 Sep 28;10(1):43–59. doi: 10.1002/iid3.539 (PMC8669697; doi:10.1002/iid3.539)
Supplement: Supplementary file 11 — Supplementary information. [file IID3-10-43-s009.docx]

**Table-S10.** Gene Ontology (GO) enrichment analyses for immunogenomic phenotype-related differentially expressed genes.

| **ONTOLOGY** | **Description** | **p.adjust** | **Count** |
| --- | --- | --- | --- |
| BP | regulation of innate immune response | 3.97E-12 | 50 |
| BP | regulation of leukocyte activation | 3.97E-12 | 53 |
| BP | negative regulation of immune system process | 7.24E-11 | 48 |
| BP | positive regulation of innate immune response | 7.97E-11 | 43 |
| BP | negative regulation of cell activation | 5.08E-10 | 29 |
| BP | negative regulation of leukocyte activation | 5.46E-10 | 27 |
| BP | regulation of lymphocyte activation | 2.43E-09 | 42 |
| BP | response to virus | 3.05E-09 | 37 |
| BP | negative regulation of lymphocyte activation | 5.94E-09 | 23 |
| BP | positive regulation of cell activation | 6.87E-09 | 36 |
| BP | defense response to other organism | 1.11E-08 | 45 |
| BP | activation of innate immune response | 2.05E-08 | 35 |
| BP | cytokine secretion | 3.11E-08 | 29 |
| BP | neutrophil degranulation | 3.71E-08 | 44 |
| BP | neutrophil activation involved in immune response | 4.29E-08 | 44 |
| BP | regulation of leukocyte cell-cell adhesion | 1.23E-07 | 32 |
| BP | cellular response to molecule of bacterial origin | 1.28E-06 | 23 |
| BP | positive regulation of cytokine production involved in immune response | 0.012877539 | 7 |
| CC | collagen-containing extracellular matrix | 1.48E-08 | 40 |
| CC | secretory granule membrane | 1.50E-08 | 33 |
| CC | external side of plasma membrane | 2.05E-07 | 25 |
| CC | side of membrane | 4.85E-06 | 32 |
| CC | extracellular matrix component | 0.01164882 | 7 |
| MF | extracellular matrix structural constituent | 4.63E-06 | 22 |
| MF | glycosaminoglycan binding | 0.000588788 | 21 |
| MF | signaling pattern recognition receptor activity | 0.001601059 | 6 |
| MF | MHC protein complex binding | 0.001601059 | 7 |
| MF | heparin binding | 0.002434045 | 16 |
