# Supplementary material for: Immunogenomic characterization in gastric cancer identifies microenvironmental and immunotherapeutically relevant gene signatures
Source: Immun Inflamm Dis. 2021 Sep 28;10(1):43–59. doi: 10.1002/iid3.539 (PMC8669697; doi:10.1002/iid3.539)
Supplement: Supplementary file 12 — Supplementary information. [file IID3-10-43-s005.docx]

**Table-S11.** LASSO coefficient of the representative immune-related genes.

| **Gene** | **Coef** | **Gene** | **Coef** |
| --- | --- | --- | --- |
| AKT3 | 0.028389283 | IGF1R | 0.053672035 |
| APOE | 0.055845228 | IL1RAP | 0.051531926 |
| BCL6 | 0.015278954 | ITGB1 | 0.321687965 |
| C2 | -0.006740931 | KLRD1 | -0.111853565 |
| C7 | 0.040908853 | LAIR2 | -0.012971248 |
| C8G | -0.069526993 | LAMP3 | -0.014534425 |
| CARD11 | 0.207292662 | LY86 | 0.060637166 |
| CASP10 | -0.031136204 | MASP2 | -0.29179668 |
| CASP8 | -0.011016649 | OAS3 | -0.012484705 |
| CCL20 | -0.013400388 | PDCD1 | -0.053532329 |
| CCL25 | -0.018997364 | RORA | 0.001342243 |
| CD1B | -0.087456627 | RRAD | 0.00066744 |
| CD276 | 0.074736079 | SLAMF7 | -0.090423006 |
| CD38 | -0.008676152 | SMPD3 | -0.056451513 |
| CD9 | 0.123123489 | SYT17 | 0.023758584 |
| CTSW | -0.067290707 | TGFB1 | 0.020800251 |
| DEFB1 | 0.075586041 | TLR7 | 0.194351108 |
| FAS | -0.067068099 | TNFRSF10B | -0.202527947 |
| IFIH1 | -0.044698043 | TNFRSF11A | -0.021270735 |
| IFNL1 | -0.006213016 | TNFRSF12A | 0.282849927 |
| TREM2 | 0.049201988 | TRAF3 | -0.040722394 |
