# Supplementary material for: Immunogenomic characterization in gastric cancer identifies microenvironmental and immunotherapeutically relevant gene signatures
Source: Immun Inflamm Dis. 2021 Sep 28;10(1):43–59. doi: 10.1002/iid3.539 (PMC8669697; doi:10.1002/iid3.539)
Supplement: Supplementary file 13 — Supplementary information. [file IID3-10-43-s003.docx]

**Table-S12.** The changes of ACRG subtypes, cluster, histology and IGCS.

| **id** | **Subtype** | **Cluster** | **Histology** | **IGCS** |
| --- | --- | --- | --- | --- |
| GSM1523984 | EMT | IGPC1 | Diffuse | Low |
| GSM1523798 | EMT | IGPC2 | Diffuse | Low |
| GSM1523906 | EMT | IGPC2 | Diffuse | Low |
| GSM1523848 | EMT | IGPC2 | Diffuse | Low |
| GSM1524058 | EMT | IGPC2 | Intestinal | High |
| GSM1523971 | EMT | IGPC3 | Diffuse | High |
| GSM1524026 | EMT | IGPC3 | Diffuse | High |
| GSM1524029 | EMT | IGPC3 | Diffuse | High |
| GSM1524059 | EMT | IGPC2 | Diffuse | High |
| GSM1523965 | EMT | IGPC2 | Diffuse | High |
| GSM1524010 | EMT | IGPC3 | Diffuse | High |
| GSM1523774 | EMT | IGPC3 | Intestinal | High |
| GSM1523948 | EMT | IGPC3 | Diffuse | High |
| GSM1524035 | EMT | IGPC3 | Mixed | High |
| GSM1523935 | EMT | IGPC2 | Diffuse | High |
| GSM1523945 | EMT | IGPC2 | Diffuse | High |
| GSM1523846 | EMT | IGPC2 | Diffuse | High |
| GSM1524030 | EMT | IGPC3 | Diffuse | High |
| GSM1523866 | EMT | IGPC2 | Intestinal | High |
| GSM1523981 | EMT | IGPC2 | Diffuse | High |
| GSM1523886 | EMT | IGPC2 | Diffuse | High |
| GSM1523850 | EMT | IGPC3 | Diffuse | High |
| GSM1524039 | EMT | IGPC2 | Diffuse | High |
| GSM1523975 | EMT | IGPC3 | Diffuse | High |
| GSM1523788 | EMT | IGPC3 | Diffuse | High |
| GSM1523796 | EMT | IGPC2 | Intestinal | High |
| GSM1523918 | EMT | IGPC3 | Diffuse | High |
| GSM1523939 | EMT | IGPC3 | Diffuse | High |
| GSM1523849 | EMT | IGPC2 | Intestinal | High |
| GSM1523858 | EMT | IGPC3 | Diffuse | High |
| GSM1523962 | EMT | IGPC3 | Diffuse | High |
| GSM1523835 | EMT | IGPC1 | Intestinal | High |
| GSM1524062 | EMT | IGPC3 | Diffuse | High |
| GSM1523949 | EMT | IGPC3 | Diffuse | High |
| GSM1523772 | EMT | IGPC3 | Diffuse | High |
| GSM1523773 | EMT | IGPC3 | Diffuse | High |
| GSM1523988 | EMT | IGPC3 | Diffuse | High |
| GSM1523982 | EMT | IGPC2 | Diffuse | High |
| GSM1523862 | EMT | IGPC3 | Diffuse | High |
| GSM1524057 | EMT | IGPC3 | Intestinal | High |
| GSM1524006 | EMT | IGPC3 | Diffuse | High |
| GSM1523968 | EMT | IGPC3 | Diffuse | High |
| GSM1523844 | EMT | IGPC1 | Intestinal | High |
| GSM1523938 | EMT | IGPC3 | Diffuse | High |
| GSM1523990 | EMT | IGPC1 | Diffuse | High |
| GSM1523771 | EMT | IGPC3 | Diffuse | High |
| GSM1523728 | MSI | IGPC2 | Intestinal | Low |
| GSM1523947 | MSI | IGPC1 | Intestinal | Low |
| GSM1523863 | MSI | IGPC2 | Mixed | Low |
| GSM1523830 | MSI | IGPC2 | Intestinal | Low |
| GSM1523776 | MSI | IGPC3 | Diffuse | Low |
| GSM1524068 | MSI | IGPC1 | Intestinal | Low |
| GSM1523821 | MSI | IGPC2 | Intestinal | Low |
| GSM1523888 | MSI | IGPC2 | Intestinal | Low |
| GSM1523910 | MSI | IGPC2 | Diffuse | Low |
| GSM1524002 | MSI | IGPC2 | Mixed | Low |
| GSM1523860 | MSI | IGPC2 | Mixed | Low |
| GSM1523864 | MSI | IGPC1 | Intestinal | Low |
| GSM1523801 | MSI | IGPC2 | Intestinal | Low |
| GSM1524069 | MSI | IGPC1 | Intestinal | Low |
| GSM1523880 | MSI | IGPC2 | Diffuse | Low |
| GSM1523770 | MSI | IGPC2 | Intestinal | Low |
| GSM1523979 | MSI | IGPC2 | Intestinal | Low |
| GSM1523851 | MSI | IGPC2 | Intestinal | Low |
| GSM1523779 | MSI | IGPC3 | Diffuse | Low |
| GSM1523921 | MSI | IGPC2 | Diffuse | Low |
| GSM1523885 | MSI | IGPC2 | Diffuse | Low |
| GSM1523929 | MSI | IGPC2 | Intestinal | Low |
| GSM1523964 | MSI | IGPC1 | Intestinal | Low |
| GSM1523876 | MSI | IGPC2 | Intestinal | Low |
| GSM1523790 | MSI | IGPC2 | Intestinal | Low |
| GSM1523811 | MSI | IGPC2 | Intestinal | Low |
| GSM1523901 | MSI | IGPC2 | Intestinal | Low |
| GSM1523933 | MSI | IGPC2 | Diffuse | Low |
| GSM1523775 | MSI | IGPC1 | Diffuse | Low |
| GSM1523909 | MSI | IGPC2 | Diffuse | Low |
| GSM1523810 | MSI | IGPC2 | Intestinal | Low |
| GSM1523838 | MSI | IGPC2 | Intestinal | Low |
| GSM1524000 | MSI | IGPC3 | Diffuse | Low |
| GSM1523867 | MSI | IGPC2 | Intestinal | Low |
| GSM1523889 | MSI | IGPC1 | Intestinal | Low |
| GSM1523870 | MSI | IGPC2 | Intestinal | Low |
| GSM1524054 | MSI | IGPC1 | Intestinal | Low |
| GSM1524041 | MSI | IGPC2 | Intestinal | Low |
| GSM1523727 | MSI | IGPC1 | Intestinal | Low |
| GSM1523791 | MSI | IGPC2 | Intestinal | Low |
| GSM1523871 | MSI | IGPC2 | Intestinal | Low |
| GSM1523800 | MSI | IGPC2 | Intestinal | Low |
| GSM1524061 | MSI | IGPC1 | Intestinal | Low |
| GSM1523744 | MSI | IGPC1 | Diffuse | Low |
| GSM1523823 | MSI | IGPC2 | Intestinal | Low |
| GSM1524032 | MSI | IGPC2 | Intestinal | Low |
| GSM1523847 | MSI | IGPC2 | Intestinal | Low |
| GSM1523952 | MSI | IGPC2 | Diffuse | High |
| GSM1523903 | MSI | IGPC1 | Intestinal | High |
| GSM1523780 | MSI | IGPC2 | Intestinal | High |
| GSM1523797 | MSI | IGPC1 | Intestinal | High |
| GSM1523785 | MSI | IGPC2 | Intestinal | High |
| GSM1523856 | MSI | IGPC2 | Diffuse | High |
| GSM1524048 | MSI | IGPC2 | Intestinal | High |
| GSM1523813 | MSI | IGPC1 | Diffuse | High |
| GSM1523983 | MSI | IGPC2 | Diffuse | High |
| GSM1523822 | MSI | IGPC2 | Intestinal | High |
| GSM1523961 | MSI | IGPC1 | Intestinal | High |
| GSM1524008 | MSI | IGPC1 | Diffuse | High |
| GSM1523911 | MSI | IGPC1 | Intestinal | High |
| GSM1523868 | MSI | IGPC2 | Mixed | High |
| GSM1523825 | MSI | IGPC1 | Intestinal | High |
| GSM1523833 | MSI | IGPC1 | Diffuse | High |
| GSM1523808 | MSI | IGPC2 | Diffuse | High |
| GSM1523805 | MSI | IGPC2 | Mixed | High |
| GSM1524025 | MSI | IGPC2 | Diffuse | High |
| GSM1523828 | MSI | IGPC2 | Intestinal | High |
| GSM1523794 | MSI | IGPC2 | Diffuse | High |
| GSM1523954 | MSS/TP53- | IGPC1 | Diffuse | Low |
| GSM1523887 | MSS/TP53- | IGPC2 | Diffuse | Low |
| GSM1523815 | MSS/TP53- | IGPC3 | Mixed | Low |
| GSM1523817 | MSS/TP53- | IGPC1 | Diffuse | Low |
| GSM1524003 | MSS/TP53- | IGPC1 | Intestinal | Low |
| GSM1524013 | MSS/TP53- | IGPC2 | Intestinal | Low |
| GSM1523950 | MSS/TP53- | IGPC1 | Intestinal | Low |
| GSM1523852 | MSS/TP53- | IGPC3 | Intestinal | Low |
| GSM1523994 | MSS/TP53- | IGPC3 | Intestinal | Low |
| GSM1523930 | MSS/TP53- | IGPC1 | Intestinal | Low |
| GSM1523826 | MSS/TP53- | IGPC2 | Intestinal | Low |
| GSM1523942 | MSS/TP53- | IGPC1 | Intestinal | Low |
| GSM1524051 | MSS/TP53- | IGPC2 | Diffuse | Low |
| GSM1523976 | MSS/TP53- | IGPC1 | Mixed | Low |
| GSM1524017 | MSS/TP53- | IGPC3 | Intestinal | Low |
| GSM1524005 | MSS/TP53- | IGPC1 | Intestinal | High |
| GSM1523837 | MSS/TP53- | IGPC1 | Intestinal | High |
| GSM1523879 | MSS/TP53- | IGPC1 | Intestinal | High |
| GSM1524019 | MSS/TP53- | IGPC3 | Diffuse | High |
| GSM1524001 | MSS/TP53- | IGPC1 | Intestinal | High |
| GSM1523905 | MSS/TP53- | IGPC1 | Diffuse | High |
| GSM1523997 | MSS/TP53- | IGPC1 | Diffuse | High |
| GSM1523999 | MSS/TP53- | IGPC1 | Intestinal | High |
| GSM1523746 | MSS/TP53- | IGPC1 | Mixed | High |
| GSM1523987 | MSS/TP53- | IGPC3 | Diffuse | High |
| GSM1523920 | MSS/TP53- | IGPC3 | Intestinal | High |
| GSM1524023 | MSS/TP53- | IGPC1 | Intestinal | High |
| GSM1523993 | MSS/TP53- | IGPC2 | Intestinal | High |
| GSM1523777 | MSS/TP53- | IGPC1 | Intestinal | High |
| GSM1523895 | MSS/TP53- | IGPC1 | Intestinal | High |
| GSM1523842 | MSS/TP53- | IGPC3 | Diffuse | High |
| GSM1523827 | MSS/TP53- | IGPC3 | Diffuse | High |
| GSM1523974 | MSS/TP53- | IGPC1 | Intestinal | High |
| GSM1523853 | MSS/TP53- | IGPC1 | Intestinal | High |
| GSM1523953 | MSS/TP53- | IGPC1 | Diffuse | High |
| GSM1524040 | MSS/TP53- | IGPC2 | Diffuse | High |
| GSM1524044 | MSS/TP53- | IGPC3 | Intestinal | High |
| GSM1523745 | MSS/TP53- | IGPC1 | Diffuse | High |
| GSM1523928 | MSS/TP53- | IGPC1 | Intestinal | High |
| GSM1524004 | MSS/TP53- | IGPC3 | Diffuse | High |
| GSM1523943 | MSS/TP53- | IGPC1 | Diffuse | High |
| GSM1524015 | MSS/TP53- | IGPC1 | Intestinal | High |
| GSM1524011 | MSS/TP53- | IGPC1 | Intestinal | High |
| GSM1523959 | MSS/TP53- | IGPC1 | Intestinal | High |
| GSM1523824 | MSS/TP53- | IGPC2 | Intestinal | High |
| GSM1523865 | MSS/TP53- | IGPC1 | Intestinal | High |
| GSM1523778 | MSS/TP53- | IGPC2 | Diffuse | High |
| GSM1523792 | MSS/TP53- | IGPC1 | Intestinal | High |
| GSM1523877 | MSS/TP53- | IGPC1 | Intestinal | High |
| GSM1523924 | MSS/TP53- | IGPC3 | Diffuse | High |
| GSM1523784 | MSS/TP53- | IGPC3 | Diffuse | High |
| GSM1523991 | MSS/TP53- | IGPC1 | Intestinal | High |
| GSM1523932 | MSS/TP53- | IGPC1 | Intestinal | High |
| GSM1523897 | MSS/TP53- | IGPC1 | Intestinal | High |
| GSM1523843 | MSS/TP53- | IGPC3 | Diffuse | High |
| GSM1524027 | MSS/TP53- | IGPC1 | Diffuse | High |
| GSM1523995 | MSS/TP53- | IGPC1 | Mixed | High |
| GSM1523904 | MSS/TP53- | IGPC3 | Intestinal | High |
| GSM1523922 | MSS/TP53- | IGPC2 | Intestinal | High |
| GSM1523769 | MSS/TP53- | IGPC2 | Diffuse | High |
| GSM1523956 | MSS/TP53- | IGPC1 | Mixed | High |
| GSM1523892 | MSS/TP53- | IGPC3 | Intestinal | High |
| GSM1523890 | MSS/TP53- | IGPC3 | Diffuse | High |
| GSM1523963 | MSS/TP53- | IGPC3 | Intestinal | High |
| GSM1523969 | MSS/TP53- | IGPC3 | Diffuse | High |
| GSM1523807 | MSS/TP53- | IGPC2 | Intestinal | High |
| GSM1523966 | MSS/TP53- | IGPC3 | Diffuse | High |
| GSM1523893 | MSS/TP53- | IGPC1 | Intestinal | High |
| GSM1523854 | MSS/TP53- | IGPC2 | Diffuse | High |
| GSM1524012 | MSS/TP53- | IGPC2 | Diffuse | High |
| GSM1524042 | MSS/TP53- | IGPC2 | Diffuse | High |
| GSM1523781 | MSS/TP53- | IGPC1 | Intestinal | High |
| GSM1523973 | MSS/TP53- | IGPC1 | Diffuse | High |
| GSM1523806 | MSS/TP53- | IGPC3 | Intestinal | High |
| GSM1524046 | MSS/TP53- | IGPC3 | Diffuse | High |
| GSM1523908 | MSS/TP53- | IGPC2 | Diffuse | High |
| GSM1523839 | MSS/TP53- | IGPC2 | Diffuse | High |
| GSM1523765 | MSS/TP53- | IGPC1 | Diffuse | High |
| GSM1523941 | MSS/TP53- | IGPC2 | Diffuse | High |
| GSM1524072 | MSS/TP53- | IGPC3 | Diffuse | High |
| GSM1523989 | MSS/TP53- | IGPC3 | Intestinal | High |
| GSM1523891 | MSS/TP53- | IGPC2 | Intestinal | High |
| GSM1523816 | MSS/TP53- | IGPC1 | Intestinal | High |
| GSM1524014 | MSS/TP53- | IGPC1 | Intestinal | High |
| GSM1523855 | MSS/TP53- | IGPC1 | Intestinal | High |
| GSM1523829 | MSS/TP53- | IGPC1 | Intestinal | High |
| GSM1523934 | MSS/TP53- | IGPC1 | Intestinal | High |
| GSM1524007 | MSS/TP53- | IGPC3 | Diffuse | High |
| GSM1524056 | MSS/TP53- | IGPC1 | Intestinal | High |
| GSM1524036 | MSS/TP53- | IGPC1 | Intestinal | High |
| GSM1523802 | MSS/TP53- | IGPC1 | Intestinal | High |
| GSM1523998 | MSS/TP53- | IGPC1 | Intestinal | High |
| GSM1523874 | MSS/TP53- | IGPC2 | Intestinal | High |
| GSM1523768 | MSS/TP53- | IGPC2 | Intestinal | High |
| GSM1523960 | MSS/TP53- | IGPC1 | Intestinal | High |
| GSM1524009 | MSS/TP53- | IGPC3 | Diffuse | High |
| GSM1524037 | MSS/TP53- | IGPC3 | Intestinal | High |
| GSM1523872 | MSS/TP53- | IGPC2 | Diffuse | High |
| GSM1523789 | MSS/TP53- | IGPC1 | Diffuse | High |
| GSM1523970 | MSS/TP53- | IGPC1 | Mixed | High |
| GSM1523940 | MSS/TP53- | IGPC1 | Intestinal | High |
| GSM1523977 | MSS/TP53- | IGPC1 | Diffuse | High |
| GSM1523925 | MSS/TP53- | IGPC1 | Diffuse | High |
| GSM1524022 | MSS/TP53- | IGPC3 | Diffuse | High |
| GSM1524052 | MSS/TP53- | IGPC1 | Diffuse | High |
| GSM1523857 | MSS/TP53- | IGPC3 | Intestinal | High |
| GSM1523972 | MSS/TP53- | IGPC1 | Mixed | High |
| GSM1524033 | MSS/TP53+ | IGPC2 | Intestinal | Low |
| GSM1523926 | MSS/TP53+ | IGPC1 | Intestinal | Low |
| GSM1523944 | MSS/TP53+ | IGPC2 | Diffuse | Low |
| GSM1523931 | MSS/TP53+ | IGPC2 | Diffuse | Low |
| GSM1523986 | MSS/TP53+ | IGPC3 | Diffuse | Low |
| GSM1523955 | MSS/TP53+ | IGPC3 | Mixed | Low |
| GSM1523992 | MSS/TP53+ | IGPC1 | Diffuse | Low |
| GSM1523898 | MSS/TP53+ | IGPC1 | Intestinal | Low |
| GSM1523958 | MSS/TP53+ | IGPC2 | Diffuse | Low |
| GSM1523836 | MSS/TP53+ | IGPC2 | Intestinal | Low |
| GSM1523818 | MSS/TP53+ | IGPC1 | Diffuse | Low |
| GSM1523927 | MSS/TP53+ | IGPC3 | Diffuse | Low |
| GSM1523861 | MSS/TP53+ | IGPC2 | Intestinal | Low |
| GSM1523967 | MSS/TP53+ | IGPC3 | Diffuse | Low |
| GSM1523793 | MSS/TP53+ | IGPC2 | Diffuse | Low |
| GSM1524043 | MSS/TP53+ | IGPC2 | Diffuse | Low |
| GSM1523809 | MSS/TP53+ | IGPC2 | Intestinal | Low |
| GSM1523729 | MSS/TP53+ | IGPC1 | Diffuse | Low |
| GSM1524047 | MSS/TP53+ | IGPC1 | Diffuse | Low |
| GSM1523923 | MSS/TP53+ | IGPC1 | Diffuse | Low |
| GSM1523951 | MSS/TP53+ | IGPC2 | Diffuse | Low |
| GSM1523873 | MSS/TP53+ | IGPC2 | Diffuse | Low |
| GSM1524016 | MSS/TP53+ | IGPC3 | Diffuse | Low |
| GSM1523946 | MSS/TP53+ | IGPC1 | Intestinal | Low |
| GSM1523894 | MSS/TP53+ | IGPC1 | Intestinal | Low |
| GSM1523985 | MSS/TP53+ | IGPC2 | Intestinal | Low |
| GSM1524018 | MSS/TP53+ | IGPC1 | Mixed | High |
| GSM1524028 | MSS/TP53+ | IGPC1 | Intestinal | High |
| GSM1523996 | MSS/TP53+ | IGPC1 | Diffuse | High |
| GSM1523883 | MSS/TP53+ | IGPC2 | Diffuse | High |
| GSM1524020 | MSS/TP53+ | IGPC3 | Intestinal | High |
| GSM1523937 | MSS/TP53+ | IGPC2 | Intestinal | High |
| GSM1523957 | MSS/TP53+ | IGPC1 | Intestinal | High |
| GSM1524038 | MSS/TP53+ | IGPC2 | Diffuse | High |
| GSM1524031 | MSS/TP53+ | IGPC1 | Intestinal | High |
| GSM1523919 | MSS/TP53+ | IGPC3 | Intestinal | High |
| GSM1524021 | MSS/TP53+ | IGPC2 | Diffuse | High |
| GSM1523803 | MSS/TP53+ | IGPC1 | Intestinal | High |
| GSM1523899 | MSS/TP53+ | IGPC1 | Intestinal | High |
| GSM1524034 | MSS/TP53+ | IGPC3 | Intestinal | High |
| GSM1523978 | MSS/TP53+ | IGPC1 | Diffuse | High |
| GSM1523787 | MSS/TP53+ | IGPC1 | Intestinal | High |
| GSM1523831 | MSS/TP53+ | IGPC1 | Diffuse | High |
| GSM1524050 | MSS/TP53+ | IGPC1 | Intestinal | High |
| GSM1523875 | MSS/TP53+ | IGPC1 | Intestinal | High |
| GSM1524024 | MSS/TP53+ | IGPC1 | Diffuse | High |
| GSM1524071 | MSS/TP53+ | IGPC1 | Intestinal | High |
| GSM1523747 | MSS/TP53+ | IGPC1 | Mixed | High |
| GSM1523882 | MSS/TP53+ | IGPC2 | Diffuse | High |
| GSM1523782 | MSS/TP53+ | IGPC1 | Intestinal | High |
| GSM1523834 | MSS/TP53+ | IGPC2 | Diffuse | High |
| GSM1523783 | MSS/TP53+ | IGPC3 | Diffuse | High |
| GSM1523812 | MSS/TP53+ | IGPC2 | Diffuse | High |
| GSM1523841 | MSS/TP53+ | IGPC3 | Mixed | High |
| GSM1523804 | MSS/TP53+ | IGPC3 | Diffuse | High |
| GSM1524070 | MSS/TP53+ | IGPC1 | Intestinal | High |
| GSM1524053 | MSS/TP53+ | IGPC1 | Intestinal | High |
| GSM1523878 | MSS/TP53+ | IGPC2 | Intestinal | High |
| GSM1523832 | MSS/TP53+ | IGPC3 | Intestinal | High |
| GSM1523814 | MSS/TP53+ | IGPC1 | Intestinal | High |
| GSM1523795 | MSS/TP53+ | IGPC1 | Intestinal | High |
| GSM1523869 | MSS/TP53+ | IGPC3 | Diffuse | High |
| GSM1524045 | MSS/TP53+ | IGPC1 | Intestinal | High |
| GSM1523786 | MSS/TP53+ | IGPC3 | Diffuse | High |
| GSM1523884 | MSS/TP53+ | IGPC3 | Diffuse | High |
| GSM1523936 | MSS/TP53+ | IGPC3 | Diffuse | High |
| GSM1523748 | MSS/TP53+ | IGPC3 | Diffuse | High |
| GSM1523896 | MSS/TP53+ | IGPC2 | Intestinal | High |
| GSM1524049 | MSS/TP53+ | IGPC3 | Intestinal | High |
| GSM1523881 | MSS/TP53+ | IGPC1 | Intestinal | High |
| GSM1523840 | MSS/TP53+ | IGPC1 | Diffuse | High |
| GSM1523980 | MSS/TP53+ | IGPC3 | Diffuse | High |
| GSM1523859 | MSS/TP53+ | IGPC1 | Intestinal | High |
| GSM1523820 | MSS/TP53+ | IGPC1 | Intestinal | High |
| GSM1523845 | MSS/TP53+ | IGPC2 | Diffuse | High |
| GSM1523819 | MSS/TP53+ | IGPC1 | Intestinal | High |
| GSM1524055 | MSS/TP53+ | IGPC1 | Intestinal | High |
| GSM1523799 | MSS/TP53+ | IGPC2 | Intestinal | High |
| GSM1524060 | MSS/TP53+ | IGPC1 | Intestinal | High |
