# Supplementary material for: Immunogenomic characterization in gastric cancer identifies microenvironmental and immunotherapeutically relevant gene signatures
Source: Immun Inflamm Dis. 2021 Sep 28;10(1):43–59. doi: 10.1002/iid3.539 (PMC8669697; doi:10.1002/iid3.539)
Supplement: Supplementary file 14 — Supplementary information. [file IID3-10-43-s007.docx]

**Table-S13.** Spearman correlation between immune checkpoint molecules and IGCS.

| **From** | **To** | **Correlation** |
| --- | --- | --- |
| CTLA-4 | IGCS | -0.244415761 |
| CTLA-4 | PD-1 | 0.26877932 |
| CTLA-4 | PD-L2 | 0.481609703 |
| PD-1 | IGCS | -0.268469589 |
| PD-1 | PD-L2 | 0.159608552 |
| PD-L1 | CTLA-4 | 0.478732053 |
| PD-L1 | IGCS | -0.294016408 |
| PD-L1 | PD-1 | 0.246865901 |
| PD-L1 | PD-L2 | 0.564509816 |
| PD-L2 | IGCS | -0.08544684 |
