# Supplementary material for: Immunogenomic characterization in gastric cancer identifies microenvironmental and immunotherapeutically relevant gene signatures
Source: Immun Inflamm Dis. 2021 Sep 28;10(1):43–59. doi: 10.1002/iid3.539 (PMC8669697; doi:10.1002/iid3.539)
Supplement: Supplementary file 15 — Supplementary information. [file IID3-10-43-s014.docx]

**Table-S14.** The statistical differences among the IGPC groups.

| **Figure 2B** | **IGPC1** | **IGPC2** | **IGPC3** | **1 vs 2** | **1 vs 3** | **2 vs 3** |
| --- | --- | --- | --- | --- | --- | --- |
| ABCB1 | 4.756338256 | 5.067625972 | 5.552206026 | 1.78E-06 | 3.59E-16 | 7.41E-07 |
| ATM | 5.131605265 | 5.240586217 | 5.372559195 | 0.001426458 | 1.40E-13 | 8.35E-05 |
| C3 | 9.961956855 | 10.83824902 | 10.95852164 | 4.57E-06 | 8.83E-07 | 0.494453853 |
| CARD11 | 5.737506299 | 5.641746198 | 5.687666312 | 0.138651503 | 0.918812683 | 0.201351451 |
| CDH1 | 8.101885897 | 7.860811981 | 7.49584939 | 0.001385972 | 2.01E-08 | 0.001736262 |
| CREBBP | 6.218292299 | 6.153599528 | 6.250128091 | 0.004169831 | 0.298245605 | 2.12E-05 |
| FN1 | 7.947960137 | 8.532313075 | 8.210479286 | 1.29E-07 | 0.02235597 | 0.002675732 |
| GTF3C1 | 7.548709974 | 7.351977443 | 7.407402442 | 3.93E-06 | 0.003750061 | 0.144151004 |
| HLA-B | 11.90093399 | 12.31782899 | 12.04554194 | 1.98E-10 | 0.067716436 | 8.69E-06 |
| IGF2R | 7.643123752 | 7.514816434 | 7.420035234 | 0.012015189 | 8.04E-06 | 0.049891562 |
| IRF2 | 7.946374419 | 8.432389962 | 8.239229987 | 7.07E-18 | 3.34E-08 | 1.01E-05 |
| JAK1 | 6.821271427 | 7.062227679 | 7.011660468 | 2.49E-10 | 6.95E-06 | 0.176370045 |
| LRP1 | 4.871906179 | 4.807824358 | 4.894113299 | 0.002869269 | 0.32575298 | 0.000612616 |
| LRRN3 | 4.353850205 | 4.575674387 | 4.874914013 | 0.000438851 | 2.10E-10 | 0.005163283 |
| MAGEC1 | 3.807229803 | 3.628217425 | 3.477978416 | 0.008327879 | 4.77E-05 | 0.096597101 |
| MRC1 | 8.00402494 | 9.541244491 | 9.088427909 | 1.22E-24 | 2.49E-15 | 0.000156723 |
| NFATC2 | 5.840376197 | 5.786769623 | 5.954410922 | 0.244051088 | 0.012561519 | 0.00014814 |
| NLRC5 | 7.271693991 | 8.27815783 | 7.796873545 | 2.20E-21 | 3.39E-08 | 6.66E-08 |
| NOTCH1 | 5.85733188 | 5.874584972 | 5.707836026 | 0.505338018 | 0.002115179 | 1.38E-05 |
| PIK3CG | 4.611635111 | 5.345107075 | 5.414981299 | 8.10E-24 | 7.43E-25 | 0.393157759 |
| TLR4 | 4.970807658 | 6.220866047 | 5.585827026 | 4.96E-29 | 5.56E-13 | 4.33E-13 |
| TP53 | 4.51558488 | 4.56462983 | 4.442369312 | 0.401683588 | 0.262192602 | 0.032282387 |
| TPTE | 2.880793598 | 2.553431217 | 2.605186935 | 0.083588202 | 0.178305322 | 0.203681632 |
| TTK | 7.715663103 | 7.571829132 | 6.307165104 | 0.174397635 | 6.21E-14 | 2.65E-14 |
|  |  |  |  |  |  |  |
| **Figure 3B** | **IGPC1** | **IGPC2** | **IGPC3** | **1 vs 2** | **1 vs 3** | **2 vs 3** |
| Activated B cell | -0.102901 | 0.038167104 | 0.09658626 | 3.41E-18 | 3.97E-25 | 0.000339836 |
| Activated CD4 T cell | 0.375097523 | 0.499574263 | 0.399773109 | 4.37E-33 | 0.002862883 | 6.74E-17 |
| Activated CD8 T cell | 0.330473203 | 0.462869902 | 0.423185885 | 1.83E-30 | 7.28E-21 | 1.82E-06 |
| B.cells.naive | -0.121951901 | -0.067241892 | -0.023975181 | 2.31E-09 | 2.75E-18 | 0.000161773 |
| Dendritic.cells.activated | 0.013421932 | 0.121922068 | 0.121564963 | 3.86E-26 | 1.33E-21 | 0.540547179 |
| Dendritic.cells.resting | 0.08841204 | 0.141583396 | 0.159293027 | 1.86E-09 | 1.50E-10 | 0.014766752 |
| Endothelial cells | -0.012823476 | 0.047251139 | 0.094063516 | 6.84E-06 | 7.27E-17 | 7.44E-05 |
| Eosinophil | -0.008508992 | 0.068548336 | 0.106462603 | 9.95E-14 | 3.68E-19 | 3.56E-05 |
| Fibroblasts | 0.389357336 | 0.483311701 | 0.531775639 | 2.78E-12 | 1.04E-17 | 0.000648575 |
| Gamma delta T cell | 0.402952774 | 0.445172878 | 0.412792704 | 3.43E-17 | 0.00945138 | 8.53E-07 |
| Immature dendritic cell | 0.372752076 | 0.41284891 | 0.406132071 | 2.13E-19 | 1.37E-14 | 0.080897505 |
| Macrophage | 0.042991516 | 0.145918728 | 0.097466645 | 7.16E-30 | 9.24E-15 | 1.51E-08 |
| Mast cell | 0.102904233 | 0.206015788 | 0.182692815 | 5.45E-19 | 1.64E-13 | 0.30953068 |
| Mast.cells.resting | -0.115823172 | -0.049668976 | -0.015356502 | 1.01E-06 | 1.67E-15 | 0.004246689 |
| MDSC | 0.298811825 | 0.470519027 | 0.421577144 | 6.77E-37 | 6.31E-25 | 3.63E-08 |
| Monocytes | -0.181567129 | -0.058113703 | -0.086145717 | 2.28E-18 | 1.39E-14 | 0.108055985 |
| Natural killer cell | 0.206895295 | 0.274840813 | 0.261126699 | 4.84E-33 | 1.45E-23 | 0.002298936 |
| Natural killer T cell | 0.108118635 | 0.163927974 | 0.124285515 | 4.04E-25 | 0.000679678 | 1.54E-14 |
| Neutrophil | -0.076018632 | 0.046960606 | -0.082268872 | 1.58E-14 | 0.952066247 | 5.40E-11 |
| NK.cells.resting | -0.147821584 | -0.123171813 | -0.128268376 | 3.35E-06 | 0.000313212 | 0.312230012 |
| Plasma.cells | -0.033649432 | 0.009042902 | 0.013992741 | 5.73E-06 | 7.08E-08 | 0.234582357 |
| Plasmacytoid dendritic cell | 0.311288431 | 0.355101073 | 0.33609683 | 1.18E-14 | 1.09E-08 | 0.002454989 |
| Regulatory T cell | 0.183712726 | 0.327813082 | 0.258167004 | 2.90E-32 | 7.14E-14 | 1.29E-14 |
| T follicular helper cell | 0.150291324 | 0.227737344 | 0.217753975 | 3.42E-28 | 3.10E-24 | 0.120355092 |
|  |  |  |  |  |  |  |
| **Figure 4A** | **IGPC1** | **IGPC2** | **IGPC3** | **1 vs 2** | **1 vs 3** | **2 vs 3** |
| EMT1 | 0.291540766 | 0.32808288 | 0.312604331 | 2.99E-05 | 0.01293659 | 0.173492316 |
| EMT2 | 0.136745283 | 0.22206023 | 0.234758632 | 5.07E-13 | 1.29E-13 | 0.302849996 |
| EMT3 | 0.239599739 | 0.309357773 | 0.356229337 | 1.93E-10 | 2.81E-18 | 0.000847039 |
| Pan-F-TBRS | 0.338189615 | 0.371024983 | 0.407141765 | 2.25E-06 | 7.43E-14 | 8.78E-05 |
| AngioIDsis | -0.146676658 | -0.056942064 | -0.035813804 | 2.09E-10 | 1.23E-14 | 0.039739955 |
| CD8 T effector | 0.220469351 | 0.441839971 | 0.335111768 | 1.05E-29 | 9.67E-13 | 1.57E-12 |
| Immune checkpoint | -0.08245493 | 0.091892745 | -0.024833825 | 1.19E-34 | 6.58E-10 | 1.54E-18 |
|  |  |  |  |  |  |  |
| **Figure 4F** | **IGPC1** | **IGPC2** | **IGPC3** | **1 vs 2** | **1 vs 3** | **2 vs 3** |
| ACTA2 | 5.782054521 | 5.992510604 | 6.713005848 | 7.49E-05 | 3.63E-08 | 7.88E-11 |
| COL4A1 | 9.662665179 | 10.20777339 | 9.992097288 | 2.79E-06 | 0.487740289 | 0.100229911 |
| PDGFRA | 5.101550658 | 5.182531906 | 5.413264833 | 0.122786572 | 0.00059133 | 1.04E-05 |
| SMAD9 | 5.177795675 | 5.027787991 | 6.013300182 | 0.122786572 | 1.95E-09 | 1.23E-13 |
| TGFB2 | 4.171500436 | 4.217342925 | 4.338858182 | 0.066631346 | 0.078809232 | 0.062688021 |
| TGFBR2 | 7.439437265 | 7.490270113 | 7.759872955 | 0.437591108 | 0.006285465 | 1.28E-05 |
| TWIST1 | 5.286566932 | 6.209804255 | 6.13005953 | 1.17E-07 | 0.609381665 | 0.531236641 |
| VIM | 7.533260983 | 8.284062472 | 8.376744379 | 2.05E-15 | 0.150212942 | 0.068911898 |
| ZEB1 | 5.549389214 | 6.205525613 | 6.802043682 | 8.25E-15 | 0.001347978 | 1.91E-05 |
|  |  |  |  |  |  |  |
| **Figure 4G** | **IGPC1** | **IGPC2** | **IGPC3** | **1 vs 2** | **1 vs 3** | **2 vs 3** |
| CD8A | 6.048565333 | 7.776209887 | 7.549148286 | 6.70E-24 | 2.57E-24 | 0.079433543 |
| CXCL10 | 8.629768915 | 10.69940375 | 9.225398299 | 1.87E-24 | 0.002078419 | 5.15E-12 |
| CXCL9 | 8.296293735 | 10.5050969 | 9.413622468 | 2.74E-24 | 6.35E-09 | 9.97E-09 |
| GZMA | 7.012440479 | 8.760795594 | 8.230309961 | 3.12E-24 | 7.61E-16 | 7.01E-05 |
| GZMB | 6.954089299 | 9.134656632 | 7.288545442 | 4.20E-31 | 0.047581169 | 1.16E-21 |
| IFNG | 4.250558624 | 5.954017255 | 4.445524195 | 9.76E-26 | 0.131211821 | 1.87E-17 |
| PRF1 | 6.26494841 | 7.257921698 | 6.656325649 | 1.97E-25 | 8.40E-09 | 3.62E-11 |
| TBX2 | 5.124584427 | 5.128059868 | 5.145464636 | 0.287745184 | 0.564416748 | 0.147422475 |
| TNF | 4.534399897 | 4.912475528 | 4.225647312 | 2.71E-07 | 3.36E-05 | 8.76E-17 |
|  |  |  |  |  |  |  |
| **Figure 4H** | **IGPC1** | **IGPC2** | **IGPC3** | **1 vs 2** | **1 vs 3** | **2 vs 3** |
| PD-L1 | 5.829735991 | 7.158884481 | 6.053622753 | 1.84E-34 | 0.001317758 | 1.98E-24 |
| CTLA-4 | 4.878709658 | 5.529534708 | 5.077183714 | 3.11E-28 | 3.82E-08 | 5.40E-15 |
| HAVCR2 | 5.064418803 | 5.680493075 | 5.339294065 | 4.14E-30 | 1.55E-11 | 9.89E-12 |
| IDO1 | 7.479011983 | 10.30741569 | 8.019152961 | 3.14E-27 | 0.002190493 | 6.21E-18 |
| LAG3 | 4.700364949 | 5.656093632 | 4.968184117 | 1.30E-20 | 3.28E-05 | 4.62E-10 |
| PD-1 | 6.175973325 | 6.185509792 | 6.164799519 | 0.954421807 | 0.338785057 | 0.209454766 |
| PD-L2 | 3.783110974 | 4.34730217 | 3.899034714 | 9.39E-28 | 0.000666808 | 2.62E-15 |
| CD80 | 3.904953701 | 4.521936849 | 3.921287857 | 1.71E-28 | 0.660603094 | 9.86E-22 |
| CD86 | 5.968257855 | 7.494518792 | 6.784013273 | 1.35E-34 | 5.83E-17 | 1.35E-14 |
| TIGIT | 4.877839974 | 5.973951575 | 5.61056574 | 4.93E-27 | 3.08E-18 | 0.000134816 |
| TNFRSF9 | 4.349335795 | 4.811979443 | 4.381688753 | 8.22E-23 | 0.088860856 | 3.35E-17 |
|  |  |  |  |  |  |  |
| **Figure S3E** | **IGPC1** | **IGPC2** | **IGPC3** | **1 vs 2** | **1 vs 3** | **2 vs 3** |
| CD40 | 5.819745205 | 6.340943085 | 6.044599403 | 1.89E-14 | 4.61E-06 | 1.30E-05 |
| CD40LG | 5.176962504 | 5.23846833 | 5.318380597 | 0.060408783 | 8.56E-05 | 0.013597234 |
| CD80 | 3.904953701 | 4.521936849 | 3.921287857 | 1.71E-28 | 0.660603094 | 9.86E-22 |
| CD86 | 5.968257855 | 7.494518792 | 6.784013273 | 1.35E-34 | 5.83E-17 | 1.35E-14 |
| HLA-A | 12.82270328 | 13.04473359 | 12.87571455 | 1.15E-05 | 0.518568813 | 8.28E-05 |
| HLA-B | 12.16764681 | 12.59379569 | 12.31546465 | 1.98E-10 | 0.067716436 | 1.16E-05 |
| HLA-C | 12.01890373 | 12.38520138 | 12.15984612 | 1.16E-10 | 0.069303692 | 6.57E-06 |
| HLA-DMA | 8.998009299 | 10.30197733 | 9.953417377 | 7.32E-16 | 9.30E-10 | 0.000821557 |
| HLA-DMB | 9.004166769 | 10.30496548 | 10.02244929 | 3.98E-23 | 5.70E-17 | 0.001395348 |
| HLA-DOA | 5.346984624 | 5.762785481 | 5.595490416 | 1.49E-18 | 1.40E-13 | 0.003421688 |
| HLA-DOB | 4.578394308 | 4.877938208 | 4.890041623 | 7.29E-11 | 1.89E-11 | 0.456367367 |
| HLA-DPA1 | 9.114693111 | 10.58727026 | 10.20417504 | 8.30E-21 | 8.00E-13 | 0.001180021 |
| HLA-DPB1 | 7.852825094 | 8.828036255 | 8.723409494 | 1.10E-22 | 6.79E-19 | 0.14274191 |
| HLA-DPB2 | 3.681450744 | 4.260691255 | 4.221329922 | 3.14E-09 | 1.71E-10 | 0.603967265 |
| HLA-DQA1 | 5.654592521 | 6.555905528 | 6.306444013 | 1.31E-07 | 0.000367615 | 0.077991195 |
| HLA-DQB1 | 6.393251581 | 7.205876245 | 6.905062143 | 1.51E-07 | 0.000332664 | 0.049627601 |
| HLA-DQB2 | 4.954282274 | 5.237208321 | 5.274916987 | 1.22E-05 | 5.70E-06 | 0.580520524 |
| HLA-DRA | 11.19002496 | 12.58676675 | 12.24037261 | 7.66E-26 | 2.06E-15 | 6.14E-06 |
| HLA-DRB4 | 4.882740017 | 5.258258123 | 5.259745104 | 0.000371999 | 0.000894214 | 0.250529867 |
| HLA-DRB6 | 5.848798658 | 6.362391104 | 6.203195039 | 8.67E-16 | 3.76E-08 | 0.000374498 |
| HLA-E | 10.91732518 | 11.4165735 | 11.23272531 | 1.69E-13 | 3.82E-06 | 6.01E-05 |
| HLA-F | 9.455261009 | 9.972006557 | 9.616302468 | 4.80E-11 | 0.068110419 | 3.98E-07 |
| HLA-G | 10.54583238 | 10.94053328 | 10.66015297 | 1.07E-10 | 0.104025439 | 8.45E-06 |
| HLA-J | 9.248476043 | 9.641380387 | 9.337892429 | 6.48E-09 | 0.257769782 | 1.19E-05 |
| ICAM1 | 6.463295205 | 7.500418425 | 6.369326442 | 4.07E-23 | 0.687323003 | 8.65E-26 |
| ICAM2 | 7.392386974 | 8.125844566 | 8.109044701 | 8.91E-13 | 1.23E-14 | 0.987595803 |
| ICAM3 | 8.213108974 | 8.785987764 | 8.609464857 | 3.32E-12 | 1.60E-06 | 0.015716397 |
|  |  |  |  |  |  |  |
| **Figure S3B** | **pValue** |  |  |  |  |  |
| diffuse vs indeterminate | 0.17 |  |  |  |  |  |
| diffuse vs intestinal | 0.013 |  |  |  |  |  |
| diffuse vs mixed | 0.19 |  |  |  |  |  |
| indeterminate vs intestinal | 0.29 |  |  |  |  |  |
| indeterminate vs mixed | 0.49 |  |  |  |  |  |
| intestinal vs mixed | 0.78 |  |  |  |  |  |
| Stage I vs Stage II | 0.014 |  |  |  |  |  |
| Stage I vs Stage III | 0.00013 |  |  |  |  |  |
| Stage I vs Stage IV | 5.00E-06 |  |  |  |  |  |
| Stage II vs Stage III | 0.036 |  |  |  |  |  |
| Stage II vs Stage IV | 0.00039 |  |  |  |  |  |
| Stage III vs Stage IV | 0.095 |  |  |  |  |  |
